# Drugging the intrinsically disordered transactivation domain of androgen receptor

**DOI:** 10.1038/s41392-026-02642-3

**Published:** 2026-04-28

**Authors:** Jon K. Obst, Carmen A. Banuelos, Kunzhong Jian, Amy H. Tien, Oleksandr A. Shkrabak, Jun Wang, Nasrin R. Mawji, Teresa Tam, Marija Vuckovic, David E. Williams, Jason C. Rogalski, Xiaojing Yuan, Natalie C. J. Strynadka, Raymond J. Andersen, Marianne D. Sadar

**Affiliations:** 1 Canada’s Michael Smith Genome Sciences Centre at BC Cancer, Vancouver, BC Canada; 2 Chemistry and Earth, Ocean and Atmospheric Sciences, University of British Columbia, Vancouver, BC Canada; 3 Dept. of Biochemistry and Molecular Biology and the Center for Blood Research, Life Sciences Centre, University of British Columbia, Vancouver, BC Canada; 4 Proteomics and Metabolomics Core Facility, Life Sciences Institute, University of British Columbia, Vancouver, BC Canada

**Keywords:** Molecular biology, Biophysics, Biochemistry, Chemical biology, Drug discovery

## Abstract

Androgen receptor (AR) is a therapeutic target for prostate cancer. Despite effectively targeting its folded ligand-binding domain (LBD), resistance ultimately develops by mechanisms involving reactivation of AR signaling. These mechanisms include expression of constitutively active AR that lacks LBD and fueled the discovery of inhibitors that bind to AR’s N-terminal intrinsically disordered transactivation domain (TAD). AR-TAD inhibitors (ARTADIs) are unique due to the paucity of small molecule inhibitors that bind directly to intrinsically disordered TADs, which have historically been considered undruggable. Leveraging our library of ARTADIs using cultured prostate cancer cells and multiple xenograft models, we reveal that small alterations in the chemical scaffold impact selectivity and potency within the AR-transcriptome; impacting signal transduction pathways involved in protumorigenic mechanisms. Mechanistically, these compounds differentially disrupt interactions between full-length AR or splice-variant AR-V7, and co-regulators, as revealed by rapid immunoprecipitation mass spectrometry of endogenous protein and the proximity ligation assay. Biophysically, several ARTADIs displayed exceptionally strong binding affinities that were better than, or were comparable to the LBD-inhibitor enzalutamide, with dissociation constants in the picomolar to low-nanomolar range as determined by surface plasmon resonance and microscale thermophoresis. MS/MS analysis revealed covalent binding to cysteine 129. In vivo, ARTADIs outperformed enzalutamide against prostate cancer xenografts in the presence of androgens, underscoring the therapeutic potential of targeting alternative AR domains. These findings support the feasibility - but also highlight the complexity - of developing drugs against an intrinsically disordered TAD impacted by multivalent binding interactions that may not occur in a stepwise fashion.

## Introduction

Transactivation domains (TADs) of transcription factors are enriched in intrinsically disordered regions (IDRs) that lack a stable three-dimensional structure. The plasticity of an IDR permits for dynamic and multiple alterations of its conformation, an ensemble, thereby extending its repertoire of interacting molecules to regulate cellular and biological functions. The ensemble properties of an IDR depend upon both its primary sequence and its cellular milieu which are influenced by the concentration of interacting molecules and post-translational modifications.^1^ The sensitivity of IDR ensembles to changes in the cellular milieu enables fine tuning in a cell-specific manner and for a transcription factor, in a gene-specific manner. Recently long IDRs have been suggested to impart specificity and decrease the search time of a transcription factor for binding to DNA sequence motifs.^2^ While progress has been made in understanding how protein disorder regulates and tunes cellular function, the lack of small molecules that bind to IDRs has limited advances in drug development against these important therapeutic targets.

The first small molecule that directly binds to an IDR to reach clinical trials was ralaniten acetate (NCT02606123). This molecule binds to the N-terminal TAD of androgen receptor (AR) within activation function-1 (AF-1).^3–5^ AR-TAD comprises approximately 556 residues and is predominantly disordered. Several scaffolds and unique structural classes of AR-TAD inhibitors (ARTADIs) have been discovered that include sintokamides and multiple ralaniten-like molecules with different structural motifs.^3,6–12^ Interest in the development of ARTADIs is driven by the fact that they are not affected by common resistance mechanisms that evolve following treatment with therapeutics targeting the folded ligand-binding domain (LBD) of AR. These resistance mechanisms include alterations to AR-LBD, such as gain-of-function mutations and loss of LBD to yield constitutively active splice variants such as AR-V7 (*AR3*).^13,14^

The advancement of a second ARTADI into clinical trials for the treatment of prostate cancer (NCT04421222; NCT05075577) provided proof-of-concept that drugs targeting an IDR are feasible. Termination of the clinical trial for first-generation ralaniten-acetate was due to pill burden which emphasized that its drug-like properties needed to be optimized.^15^ We have analysed more than 560 ARTADIs to optimize potency (see supplemental references^8–25^). This library of compounds provides an opportunity to study the impact of specific chemical structures of ARTADIs against AR activities in terms of affinity, potency, specificity, gene expression, in vivo efficacy and impact on cellular function and pathways; providing a roadmap for developing next-generation ARTADI compounds. Here, we explore the feasibility of ARTADIs as therapeutics in the presence of androgens as well as in cells and xenografts driven by AR-V7.

## Results

### Halogens on the phenyl rings of ralaniten improve potency

**Fig. 1:**
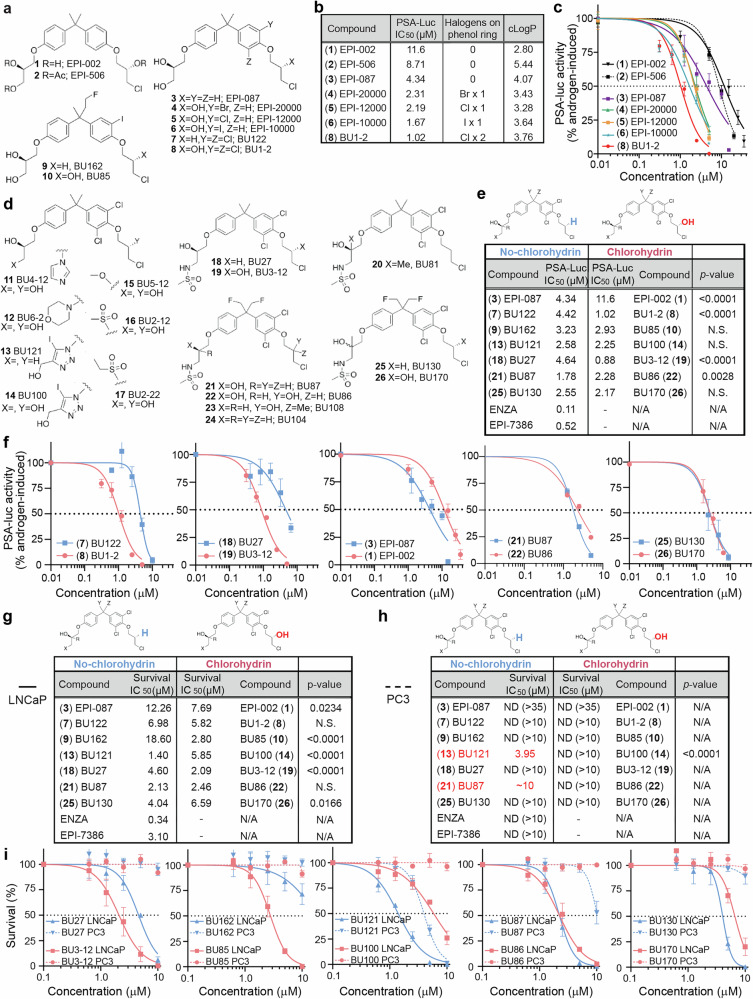
Improving the potency of compounds. **a** Structures of compounds (**1–10**). **b** CLogPs and IC_50_s for blocking androgen-induced PSA-luciferase activities in LNCaP cells for compounds (**1–8**). **c** Dose response curves of inhibition of androgen-induced PSA-luciferase activity. **d** Chemical structures showing the route to BU130/BU170. **e**, **f** IC_50_s for pairs of compounds which differed only by the presence (right, red) or absence (left, blue) of the chlorohydrin group using the PSA-luciferase reporter in androgen-induced LNCaP cells. **g** Table showing the IC_50_s derived from the colony formation assays in LNCaP cells or (**h**) PC3 cells. **i** Dose response curves from the colony formation assay using LNCaP (solid lines) or PC3 (dashed lines) cells. Blue lines: compounds lacking the chlorohydrin functional group. Red lines: matched compounds with the chlorohydrin. n.s.: not significant. ND: not detected. N/A: not available. Error bars: mean ± SEM. See Supplementary Fig. 1

Inspiration to enhance potency while still maintaining specificity came from the imaging agent, (**6**) iodoralaniten/EPI-10000, that was ≈10-fold more potent compared to (**1**) ralaniten/EPI-002 at blocking full-length AR and AR-V7 driven transcriptional activities and cellular proliferation.^16^ Consistent with those data, replacement of the iodine substituent with a single bromine or chlorine atom in (**4**) EPI-20000 and (**5**) EPI-12,000, respectively, was ≈5-fold more potent than (**1**). Adding a second chlorine atom gave (**8**) BU1-2 that resulted in ≈10-fold better potency than (**1**), (Fig. 1a–c). Thus, subsequent analogs had dichlorinated bisphenol rings.

### Removing primary and secondary alcohols

We next endeavored to resolve the metabolic liabilities of (**1**) ralaniten, which resulted in its failure in clinical trials. To this end we modified the primary alcohol on C1 which was identified in the Phase I clinical trial as a major source of oxidation.^15^ A series of compounds were generated (**11**-**19**) with various functional groups replacing the primary alcohol; imidazole [(**11)**, BU4-12], morpholine [(**12)**, BU6-2], triazole [(**13)**, BU121; (**14)**, BU100], methylether [(**15)**, BU5-12], methyl sulfone [(**16)**, BU2-12], ethyl sulfone (**17**, BU2-22) and methyl sulfonamide [(**18)**, BU27; (**19)**, BU3-12; Fig. 1d]. Supplementary Fig. 1a shows structures of control compounds enzalutamide and masofaniten (EPI-7386). While all of these compounds were quite potent with IC_50_s between 1–4 µM, the CLogP generally became less favorable (Supplementary Fig. 1b,c). Compounds (**16**-**19)** which had methyl sulfone, ethyl sulfone or methyl sulfonamide groups were superior. Of these, (**19**) BU3-12 showed the greatest potency with the best pharmacological profile with limited evidence of off-target effects and a favorable CLogP. (**19**) BU3-12, aka EPI-7170, has shown therapeutic potential in vivo in multiple prostate cancer models.^8,9,17,18^

The secondary alcohols on C3 and C20 of ralaniten are potential sites of metabolism, with the latter shown to be a target of glucuronidation.^15^ Removal of these secondary alcohols was investigated with compounds (**3**) EPI-087, (**18**) BU27 and (**20**) BU81 which lacked the chlorohydrin alcohol and had the other secondary alcohol converted to a tertiary alcohol to potentially block oxidation and provide steric hindrance to glucuronidation and sulfation. These compounds showed reasonable potency (Supplementary Fig. 1b,c). Unfortunately, the loss of the secondary alcohols or addition of methyl groups to create tertiary alcohols increased their CLogPs [CLogPs: (**3)**, 4.07; (**18)**, 5.15; (**20)**, 5.55]. ClogPs over a value of 5 become challenging to evaluate in vitro or administer to animals due to poor solubility.

Calculations showed that adding a single fluorine atom to each of the bridging methyl groups of (**18**) BU27 and (**20**) BU81 should reduce their CLogP values. Therefore, the difluorinated compound (**21)** (BU87, CLogP = 3.70); (**22)** (BU86, CLogP = 2.43); (**23)** (BU108, CLogP = 3.99); (**24)** (BU104, CLogP = 4.87); (**25)** (BU130, CLogP = 4.10); and (**26)** (BU170, CLogP = 2.83) were prepared and evaluated (Supplementary Fig. 1c, d). No obvious trend was observed between potency and ClogP values.

In vivo testing using castrated hosts bearing LNCaP xenografts was employed to test several of the more potent ARTADIs designed to be metabolically stable. (**17**) BU2-22 and (**19**) BU3-12 were the most efficacious when compared to equal molarity doses of (**8**) BU1-2 and (**2**) ralaniten-acetate (EPI-506)(Supplementary Fig. 1e). At equal doses of 30 mg/kg body weight, (**19**) BU3-12 was better than (**8**) BU1-2; (**14**) BU100; and (**18**) BU27 (Supplementary Fig. 1f). Compounds (**8**) BU1-2 dosed daily at 20 and 30 mg/kg body weight and (**18**) BU27 at 30 mg/kg body weight had no significant impact on tumor growth. From these studies, (**19**) BU3-12 was selected as a compound of interest for further investigation.

### Loss of the chlorohydrin group

The chlorohydrin group of ralaniten and other ARTADIs may be essential for their binding mechanism to AR-TAD.^4,5,12^ Here we investigated the potency and specificity of novel compounds lacking this chemical group compared to its matching chlorohydrin-containing analogue; a difference of a single atom. We expected that destroying the chlorohydrin would reduce the ability of the compound to bind to the AR-TAD and inhibit activity. Initial testing using the PSA-Luc reporter was inconclusive. In two comparisons [(**7**), BU122 vs (**8**), BU1-2; and (**18**), BU27 vs (**19**), BU3-12] highly significant differences in IC50 were observed (Fig. 1e, f). Additionally, adding a methyl group to the chlorohydrin as seen in (**23**) BU108 reduced potency ~4-fold supporting this hypothesis (Supplementary Fig. 1c). Conversely, the comparisons of (**3**) EPI-087 vs (**1**) EPI-002; and (**21**) BU87 vs (**22**) BU86 indicated that analogs lacking the chlorohydrin were more potent. The remaining comparisons were not significant (Fig. 1e, f and Supplementary Fig. 1c, g).

A stronger link was seen when measuring growth and survival of AR-dependent LNCaP cells. In this case, loss of the chlorohydrin tended to impact potency (e.g., BU3-12 vs BU27; Fig. 1g). In the case of (**8**) BU1-2, while the difference was not significant there was a trend of improved potency associated with the chlorohydrin (Supplementary Fig. 1h). The least potent compounds against LNCaP growth and survival were (**3**) EPI-087 and (**9**) BU162 that lacked a chlorohydrin (Fig. 1g and Supplementary Fig. 1h).

AR-deficient PC3 cells are independent of AR for growth and survival making this a model for off-target effects. The only compounds which had any measurable effect on PC3 survivability were (**13**) BU121 and (**21**) BU87 analogs which lack the chlorohydrin functional group (Fig. 1h, i). Taken together, these results imply that the chlorohydrin is generally important for on-target activity and potency. An interesting exception was seen where (**25**) BU130 (no chlorohydrin) showed similar or slightly better potency compared to (**26**) BU170 (chlorohydrin) with minimal effect on PC3 growth. Enzalutamide has outstanding potency and specificity against the growth and survival of LNCaP cells as expected (Fig. 1g, h and Supplementary Fig. 1h). Masofaniten (EPI-7386) is tricyclic ARTADI that lacks a chlorohydrin and recently failed clinical trials (NCT04421222; NCT05075577). Several compounds showed improved potency compared to EPI-7386 in their ability to inhibit LNCaP survival while maintaining a therapeutic window estimated to be greater than 10-fold over PC3 survival; namely (**10**) BU85, (**19**) BU3-12, and (**22**) BU86. Of these, compound (**19**) was superior (BU3-12; 2.09 µM vs. EPI-7386; 3.10 µM; *p* = 0.0048, Fig. 1g–i and Supplementary Fig. 1h).

**Fig. 2:**
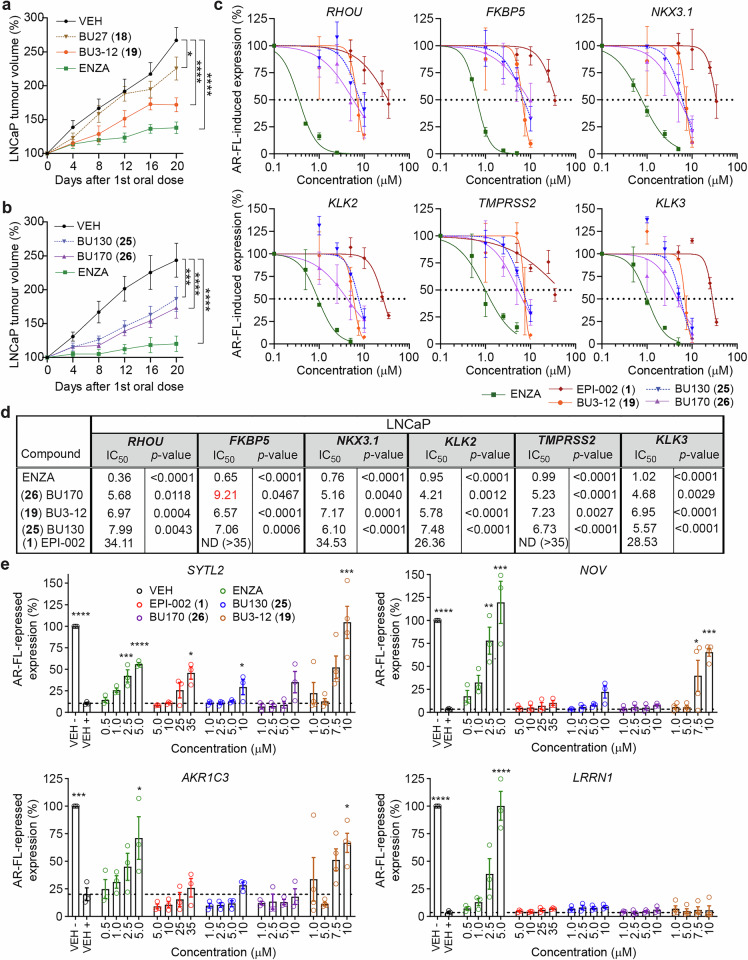
Efficacy and IC50s to block endogenous expression of genes transcriptionally regulated by full-length AR. **a** LNCaP xenografts grown in castrated mice dosed by gavage daily with vehicle (VEH), BU27 (30 mg/kg), its matching analogue BU3-12 (30 mg/kg), and enzalutamide (10 mg/kg). **b** LNCaP xenografts grown in castrated mice dosed by gavage daily with vehicle (VEH), BU130 (30 mg/kg), its matching analogue BU170 (30 mg/kg), and enzalutamide (10 mg/kg). **c** mRNA levels of androgen-induced genes normalized to housekeeping gene *SDHA* from LNCaP cells. Data presented as mean ± SEM and normalized to DMSO vehicle control in presence of R1881 (*n* = 3 independent experiments). **d** Table showing IC50 values for each inhibitor for genes shown in **c**. The sum-of-squares F test was performed to compare IC50’s for each inhibitor against EPI-002 (ralaniten). **e** Transcript levels of androgen-repressed genes normalized to levels of housekeeping gene *SDHA* from LNCaP cells treated as in **c**. Data presented as mean ± SEM, and analyzed by one-way ANOVA with Dunnet’s correction (*n* = 3 independent experiments). ND: not detected. *p < 0.05; **p < 0.01; ***p < 0.001; ****p < 0.0001

Comparison of the in vivo efficacy of analogs with and without a chlorohydrin against the growth of LNCaP xenografts revealed that the chlorohydrin analog was significantly better when comparing (**18**) BU27 versus (**19**) BU3-12 (Fig. 2a). As before, there was only a slight/negligible improvement with the chlorohydrin when comparing the efficacy of (**25**) BU130 versus (**26**) BU170 (Fig. 2b). Therefore, (**19**) BU3-12, (**25**) BU130 and (**26**) BU170 were selected for further analysis.

### IC_50_s to block the endogenous expression of genes transcriptionally regulated by full-length AR

To better characterize the next generation of ARTADIs, we examined the effect of BU3-12, BU130 and BU170 on canonical full-length AR-regulated genes compared to enzalutamide and ralaniten. Our aims were to demonstrate superiority of second-generation ARTADIs and identify potential differences between BU170 with a chlorohydrin vs BU130 that lacks a chlorohydrin. All three second-generation ARTADIs were substantially more potent than ralaniten. However, enzalutamide was the most effective to block androgen-induced expression of this set of genes (Fig. 2c, d).

Gene-specific sensitivities were observed depending upon the inhibitor; i.e., androgen-induced expression of *RHOU* was sensitive to enzalutamide, with a 3-fold decrease in IC_50_ compared to *KLK3*. Conversely the ARTADIs inhibited gene expression all to a similar degree with IC50s in a much tighter range (~6–7 µM for BU130 and BU3-12, and 4–5 µM for BU170) (Fig. 2d). BU170 was the exception with androgen-induced expression of *FKBP5* considerably less-sensitive to inhibition compared to the other genes (1.5- to 2-fold difference, Fig. 2c, d). BU170 was generally superior to BU130 at inhibiting androgen-induced gene expression.

Approximately one-half of the androgen-regulated transcriptome in prostate cells is repressed by androgen by a multitude of proposed mechanisms involving various AR domains.^19,20^ Therefore we looked at several well-characterized androgen-repressed genes and investigated the ability of ARTADIs to mediate their derepression. As with androgen-induced genes, there were gene-specific differences in sensitivities to the various inhibitors. While none of the ARTADIs were capable of derepressing *LRRN1* expression, BU3-12 was capable of significantly derepressing the remainder of genes tested in a dose-dependent manner. Conversely BU130 and BU170 were fairly similar to each other and ralaniten (Fig. 2e). Together these data imply that small changes in the chemistry of ARTADIs have consequences on their ability to impact specific cellular signaling pathways.

### Activity of ARTADIs against constitutively active AR-V7 splice variant

**Fig. 3:**
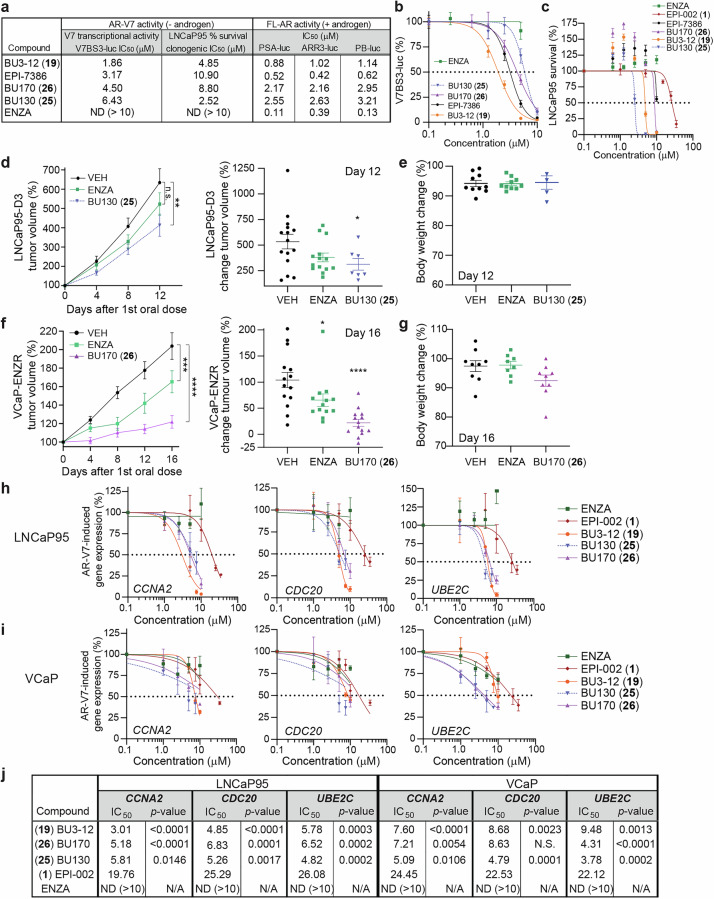
Activity of ARTADIs against constitutively active AR-V7 splice variant. **a** Table showing IC_50_ values for the V7BS3-luciferase reporter activities and clonogenic assay of LNCaP95 cells that are both driven by AR-V7 compared to androgen-induced full-length AR driven reporters. **b** Dose response curves of AR-V7 driven V7BS3-luciferase reporter activities in LNCaP cells transiently transfected to express AR-V7 protein. **c** Survival curves using the colony formation assay with LNCaP95 cells exposed to compounds. Data represents mean ± SEM, *n* ≥ 3. **d** Left panel - temporal tumor growth of subcutaneous LNCaP95-D3 xenografts in castrated NSG mice receiving vehicle (VEH), enzalutamide (ENZA, 10 mg/kg), or BU130 (30 mg/kg) daily by oral gavage. Right panel shows the final tumor volumes. **e** Body weight changes for each mouse represented in Fig. 3d. **f** Left panel - temporal tumor growth of subcutaneous VCaP-ENZR xenografts in castrated NSG mice receiving vehicle (VEH), enzalutamide (ENZA, 10 mg/kg), or BU170 (30 mg/kg) daily by oral gavage. Right panel shows the final tumor volumes. **g** Body weight changes for each mouse represented in Fig. 3f. **h** Dose-response curves of transcript levels of *AR3*/AR-V7 regulated genes from LNCaP95 cells, and **i** VCaP cells treated with enzalutamide, EPI-002 (ralaniten), BU3-12, BU130 or BU170 for 48 h in steroid-depleted media. DMSO was used as reference for normalization. Data presented as mean ± SEM (*n* = 4 independent experiments). **j** Table showing IC_50_ values to block endogenous expression of the genes shown in **h**, **i**. The sum-of-squares F test was performed to compare IC50’s for each inhibitor against EPI-002. n.s., not significant; *p < 0.05; **p < 0.01; ***p < 0.001; ****p < 0.0001. ND: not detected. N/A: not available

A mechanism of resistance that develops in prostate cancer in response to androgen ablation and antiandrogens including enzalutamide is the expression of constitutively active AR splice variant, AR-V7.^14,21–23^ AR-V7 can dimerize with the full-length AR to interact on regulatory regions of some androgen-induced genes such as *KLK3*.^24,25^ However, the *UBE2C* gene has regulatory regions that are specific for AR-V7 with full-length AR unable to interact to alter its transcription.^24,25^ This discovery was exploited to create a luciferase reporter with three repeat regions of the AR-V7 binding site (V7BS3-luc) that is induced by approximately 5 to 10-fold with ectopic expression of AR-V7 in LNCaP cells. Using this reporter, BU3-12 was the most potent compound with an IC_50_ of 1.86 μM (Fig. 3a, b) which was in the same range as for reporters driven by full-length AR (PSA-luc, ARR3-luc, and PB-luc). Masofaniten (EPI-7386) had an IC_50_ of 3.17 μM to block V7-driven transcriptional activity which was approximately 6-fold less potent than its IC_50_ to inhibit androgen-induced reporters (PSA-luc, ARR3-luc, and PB-luc). As expected, enzalutamide had no impact on AR-V7 transcriptional activity.

Growth and survival of LNCaP95 cells are dependent on functional AR-V7.^23,26^ The clonogenic assay in this cell line revealed that BU130 was the most potent of the compounds tested with an IC_50_ of 2.52 μM (Fig. 3a,c). This was unexpected since the IC_50_ for the survival of LNCaP cells for BU130 was 4.04 μM and it had an IC_50_ of 6.43 μM to block AR-V7 transcriptional activity as measured with the V7BS3-luc reporter. Masofaniten and BU170 had IC_50_s of 10.9 μM and 8.8 μM to block the growth and survival of LNCaP95 cells. BU3-12 was substantially better with an IC_50_ of 4.85 μM. Enzalutamide had no effect on these cells as reported previously.^23,26^

Since BU130 had the lowest IC_50_ against the growth and survival of LNCaP95 cells, we tested it in vivo using AR-V7 driven LNCaP95-D3 xenografts in castrated hosts.^27^ Although BU130 showed significant inhibition of this quickly growing tumor, the results seemed modest (Fig. 3d) compared to those reported at longer durations for BU3-12 (aka EPI-7170).^8,17,18^ Body weight did not change with daily dosing of BU130 over the course of the experiment (Fig. 3e).

Using the VCaP-ENZR xenograft model that is resistant to enzalutamide by a mechanism dependent upon elevated levels of expression of AR-V7,^9^ we tested the efficacy of oral delivery of BU170 (30 mg/kg body weight) to castrated hosts. BU170 was effective in blocking tumor growth (Tumor Growth Inhibition, TGI% = 79%), with some tumors becoming static while others had regression (Fig. 3f) compared to BU3-12 with a TGI% = 46%.^9^ Animals receiving BU170 maintained their body weights over the course of the experiment (Fig. 3g). Enzalutamide had modest impact on tumor growth as expected.

### Endogenous gene expression reveals gene- and cell-specificity of ARTADIs

The disparity in potencies using the LNCaP95 clonogenic assay compared to the V7BS3-luc reporter gene assay for BU130 and BU170 that differ by the loss of the chlorohydrin was unexpected. Thus, the IC_50_s of these analogs were analyzed measuring mRNA levels of *CCNA2*, *CDC20*, and *UBE2C*. Expression of these genes is regulated by AR-V7 in prostate cancer cell lines that express AR-V7.^9,20,23,28^ Additionally, BU3-12 was included with enzalutamide and ralaniten as controls. All ARTADIs blocked expression of AR-V7 regulated genes in both LNCaP95 and VCaP cells while enzalutamide had no effect as expected (Fig. 3h–j). BU3-12, BU130 and BU170 were all superior to ralaniten.

Cell-specific differences were revealed in blocking expression of these genes; i.e., the IC_50_ for BU3-12 to inhibit the expression of *CCNA2* was 3.01 μM in LNCaP95 and 7.6 μM in VCaP cells. Interestingly, in LNCaP95 cells, *CCNA2* gene expression was substantially more sensitive compared to *CDC20* and *UBE2C* to inhibition with BU3-12. Whereas, VCaP cells were most sensitive to BU130 with inhibition of *UBE2C* expression having an IC_50_ of 3.78 μM compared to an IC_50_ of 9.48 μM for BU3-12. A possible explanation is that VCaP cells have amplified *AR* and express high levels of AR relative to LNCaP and LNCaP95 cells.^9,29,30^ These data suggest that minor modifications in the chemical scaffold of ARTADIs have biological impact that may be gene- and cell-specific.

### ARTADIs are unique compared to enzalutamide in blocking the cell cycle and DNA-damage repair pathways in AR-V7 dependent cells

To date, the most potent and specific ARTADI against AR including AR-V7 is BU3-12 based upon IC_50_s for clonogenic assays, PSA- and V7BS3-luciferase reporter activities, and antitumor activity. To expand the analyses of impact of these inhibitors on gene expression in an AR-V7-dependent cell line, we employed RNA-sequencing in LNCaP95 cells in the absence of androgen (Supplementary Fig. 2a). Full-length AR is not active in LNCaP95 cells in the absence of androgen and not required for the regulation of most AR-V7 regulated genes.^20^

Principal component analyses (PCAs) revealed that enzalutamide clustered with vehicle control suggesting little impact on gene expression in this cell line (Supplementary Fig. 2b). Interestingly, BU3-12 and ralaniten did not cluster together. Gene expression which was positively (VEH_UP) or negatively (VEH_DWN) associated with vehicle treatment was used to define a baseline gene set (Supplementary Fig. 2c), and the abilities of ralaniten, BU3-12 or enzalutamide to inhibit or derepress expression of these genes were investigated.

**Fig. 4:**
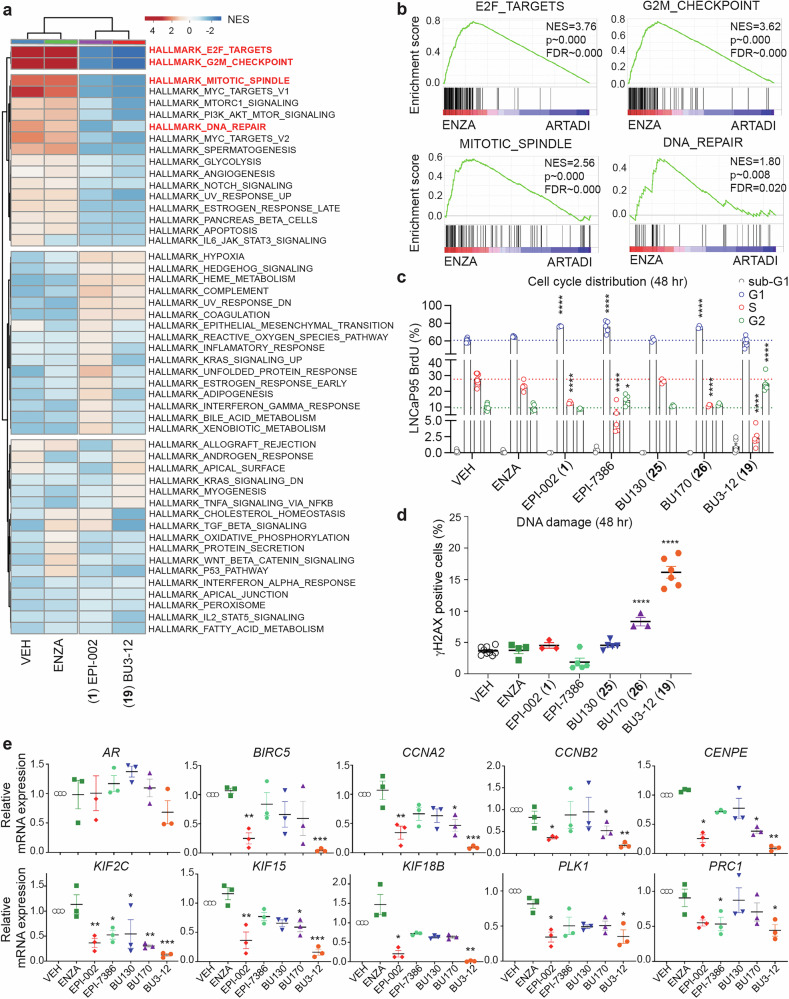
ARTADIs are unique compared to enzalutamide in blocking the cell cycle and DNA-damage repair pathways in AR-V7-dependent cells. **a** Heatmap displaying normalized enrichment scores (NES) using gene set enrichment analysis (GSEA) on RNA-seq data from androgen-deprived LNCaP95 cells treated with enzalutamide (ENZA, 5 µM), ralaniten (EPI-002, 35 µM), BU3-12 (5 µM), or DMSO (VEH) for 48 h. Gene sets were restricted to MSigDB set H (hallmark gene sets). Gene sets with nominal *p* < 0.05 and false discovery rate (FDR) *q* < 0.05 were considered significant. **b** Enrichment plots from selected gene sets showing enriched genes in ENZA treated samples compared to ARTADI (EPI-002, BU3-12). **c** Cell cycle distribution of LNCaP95 cells labeled with BrdU-FITC and 7-AAD after treatment with DMSO (VEH), enzalutamide (ENZA, 5 µM), ralaniten (EPI-002, 35 µM), masofaniten (EPI-7386, 5 µM), BU130 (5 µM), BU170 (10 µM), or BU3-12 (5 µM) determined by flow cytometry. **d** Percentage of γH2AX positive LNCaP95 cells after same treatment as in part **c**. For (**c**, **d**), bars represent the mean ± SEM of 3 independent experiments analyzed with two-way ANOVA and Tukey correction. **e** Transcript levels of *AR* and cell cycle related genes normalized to housekeeping gene *SDHA*. LNCaP95 cells were treated with DMSO (VEH), enzalutamide (ENZA, 5 µM), ralaniten (EPI-002, 35 µM), masofaniten (EPI-7386, 5 µM), BU130 (5 µM), BU170 (10 µM), or BU3-12 (5 µM) for 48 h in media supplemented with 1.5% CSS. Data presented as mean ± SEM and normalized to DMSO vehicle and were analyzed by one-way ANOVA with Dunnett’s correction (*n* = 3 independent experiments). *p < 0.05, ** *p* < 0.01, ***p < 0.001, ****p < 0.0001. See also Supplementary Figs. 2 and 3

As expected, enzalutamide did not significantly affect gene expression for the majority of these genes (only 34% of up-regulated genes and 36% of down-regulated genes) (Supplementary Fig. 2d). Both ralaniten (EPI-002) and BU3-12 were highly effective at reducing expression of genes positively associated with vehicle (EPI-002, 87%; BU3-12, 86% of genes) as well as increasing expression of genes negatively associated with vehicle treatment (EPI-002, 88%; BU3-12, 89% of genes). There was a strong overlap in the specific genes for which expression was inhibited by both ralaniten and BU3-12 (89%) as well as those which showed increased expression (89%). Additionally, expression of the majority of genes affected by enzalutamide were also affected by ralaniten, BU3-12 or both. Gene Set Enrichment Analysis (GSEA) revealed that enzalutamide impacted few pathways compared to vehicle control (Fig. 4a). Conversely both BU3-12 and ralaniten impacted gene expression involved in key signaling pathways; namely E2F targets, G2M checkpoint, mitotic spindle, and DNA-repair (Fig. 4a, b).

### Impact of ARTADIs on the cell cycle and DNA damage in AR-V7-dependent LNCaP95 cells

Pathway analyses revealed that ARTADIs uniquely impact the cell cycle and DNA-repair pathways in LNCaP95 cells, therefore we examined the effect of ARTADIs directly on these pathways using FACS analyses and staining with γH2AX, respectively. Amongst the ARTADIs examined, BU3-12 was unique in causing accumulation of LNCaP95 cells in G2 (Fig. 4c, Supplementary Fig. 2e–g). Enzalutamide and BU130 had negligible effects on cell cycle, whereas ralaniten, BU170, and masofaniten (EPI-7386) induced G1 arrest. Of the ARTADIs tested, BU3-12 had the greatest impact on inducing DNA-damage, followed by BU170 (Fig. 4d, Supplementary Fig. 2h–i). Ralaniten, BU130, masofaniten and enzalutamide had no significant effects.

Analyses of expression of specific genes identified (Supplementary Fig. 3) through GSEA involved in survival, proliferation, cell cycle progression, and spindle formation in LNCaP95 cells revealed that BU3-12 was consistently the best performer of all ARTADIs tested and significantly reduced the expression of *BIRC5*, *CCNA2*, *CCNB2*, *CENPE*, *KIF2C*, *KIF15*, *KIF18B*, *PLK1*, and *PRC1* (Fig. 4e). There were no significant differences in levels of expression of *AR* measured for any of the inhibitors. Consistent with the lack of impact of enzalutamide on cell cycle in this cell line, it had no significant effects on expression of the genes tested. These data highlight the structural/activity relationship that exists following slight chemical modifications to various ARTADIs.

### Impact of ARTADIs on the cell cycle and DNA damage in androgen-stimulated cells that are dependent on full-length AR

**Fig. 5:**
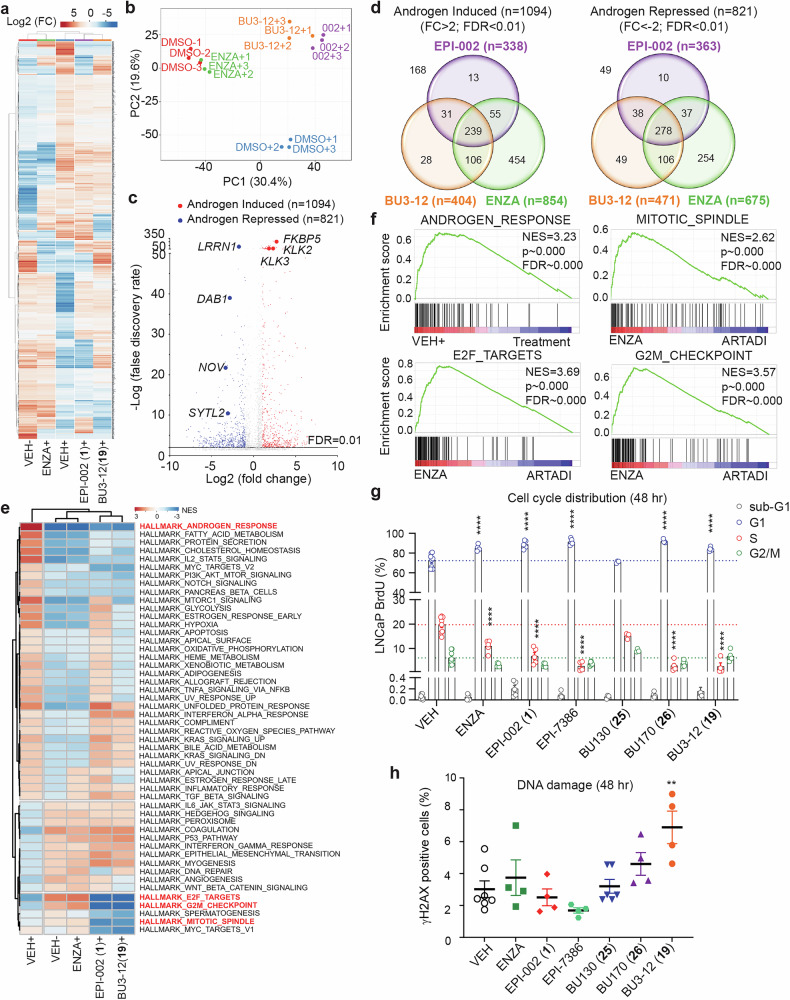
ARTADIs are unique compared to enzalutamide in blocking the cell cycle and DNA-damage repair pathways in cells that are dependent on full-length AR. **a** Heatmap of normalized signal intensity following RNA-seq transcriptional profiling of LNCaP cells pre-treated with 5 μM enzalutamide (ENZA), 35 μM EPI-002 (ralaniten), 5 μM BU3-12 or DMSO (VEH) for 16 h and stimulated with 1 nM R1881 (+) for 24 h from *n* = 3 biological replicates. **b** PCA plot based upon transcript counts for 3793 genes. **c** Volcano plot depicting androgen-regulated genes defined as genes which with a 2-fold change in expression (VEH+ vs VEH-) and FDR < 0.01. 1094 and 821 genes were defined as androgen-induced and androgen-repressed respectively. **d** Venn diagrams showing the ability of EPI-002, BU3-12 and/or enzalutamide to repress androgen-induced genes (left) or derepress androgen-repressed genes 2-fold or greater. 168 androgen-induced genes and 49 androgen-repressed genes were not affected by any treatment. **e** Heatmap showing normalized enrichment scores (NES) from GSEA. Gene sets were restricted to MSigDB set H (hallmark gene sets). Gene sets with nominal *p* < 0.05 and FDR *q* < 0.05 were considered significant. **f** Enrichment plots from selected gene sets comparing all treatments versus vehicle plus androgen (VEH + , top left) or ARTADI inhibitors EPI-002 and BU3-12 versus enzalutamide (top right and bottom). **g** Cell cycle distribution of LNCaP cells exposed for 48 h to DMSO, enzalutamide (ENZA, 10 µM), EPI-002 (35 µM), EPI-7386 (10 µM), BU130 (5 µM), BU170 (10 µM), and BU3-12 (10 µM). **h** γH2AX-positive LNCaP cells treated as in (**g**). Data in (**g**, **h**) represent the mean ± SEM of 3 independent experiments analyzed with one-way ANOVA test with Tukey correction. **p < 0.01, ****p < 0.0001. See also Supplementary Figs. 4 and 5

To determine if the unique impact of ARTADIs on the cell cycle pathways compared to enzalutamide also occurred in an androgen-stimulated cell line that is dependent on the activity of full-length AR, we examined differential gene expression using RNA-sequencing in LNCaP cells (Fig. 5a). PCA revealed that gene expression profiles from androgen-stimulated cells treated with enzalutamide clustered with profiles obtained from cells not treated with androgen (DMSO control; Fig. 5b). Gene expression profiles from cells treated with BU3-12 and ralaniten clustered together to support their similar impact on androgen-induced gene expression. Overall 1,094 genes increased expression and 821 were repressed in response to androgen which defined the androgen responsive gene set (Fig. 5c). Venn diagrams illustrate androgen-induced and androgen-repressed expression of genes impacted by ARTADIs and enzalutamide (Fig. 5d). Overall, BU3-12 showed more commonality with enzalutamide than ralaniten, which is likely due its increased potency compared to ralaniten.

A GSEA pathway analysis revealed differences between the ARTADIs and enzalutamide. All three inhibitors blocked the androgen-response, fatty acid metabolism, and cholesterol homeostasis pathways thereby supporting that they target AR-regulated pathways. Consistent to that observed in LNCaP95 cells, E2F targets, G2M checkpoint, and mitotic spindle pathways were uniquely affected by ARTADI treatment (Fig. 5e, f). However, only ralaniten inhibited expression of genes involved in DNA-repair in contrast to what was observed in LNCaP95 cells (Fig. 4a, b and Fig. 5e).

To validate these findings we examined the cell cycle and DNA damage following treatment with ARTADIs compared to enzalutamide. Inhibitors of androgen-induced full-length AR mediate accumulation of prostate cancer cells in G1.^4,8,31^ Consistent with those reports, cell cycle analyses revealed that all inhibitors, except for BU130, significantly increased cells in G1 and decreased cells in S-phase (Fig. 5g and Supplementary Fig. 4a). Notably, while BU3-12 caused accumulation of AR-V7-driven LNCaP95 cells in G2, androgen-induced LNCaP cells driven by full-length AR accumulated in G1. BU3-12 also increased γH2AX staining in LNCaP cells (Fig. 5h and Supplementary Fig. 4b, c). Neither enzalutamide nor any other ARTADI including masofaniten had significant impact on DNA-damage. Together these data signify gene-specific and cell-specific effects and sensitivities to inhibitors of full-length AR and AR-V7.

We next looked at expression of genes which were identified by GSEA. All inhibitors were effective at blocking induction of androgen-regulated genes with minimal effect on *AR* expression (Supplementary Fig. 4d). Differences revealed were with levels of expression of genes involved in E2F targets, G2M checkpoint, and mitotic spindle. On the whole, ARTADIs were superior to enzalutamide in blocking expression of cell cycle related genes even in the context of full-length AR stimulated with androgen.

### Binding of ARTADIs to AR-TAD

**Fig. 6:**
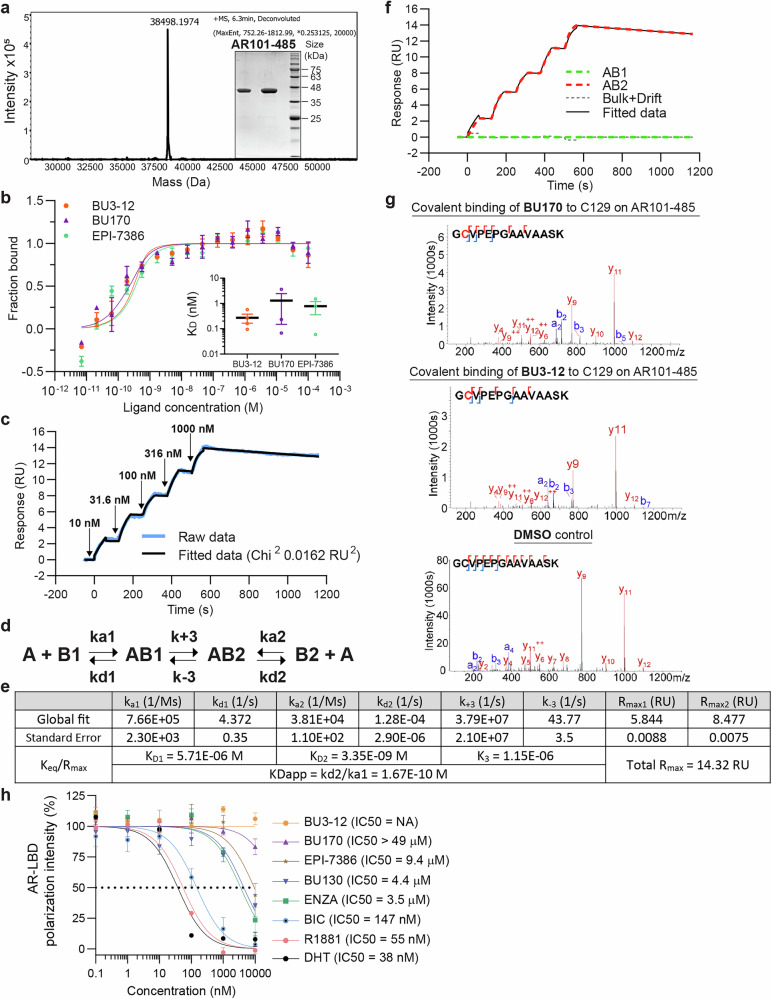
Direct binding of ARTADIs to purified AR-TAD. **a** Confirmation of AR101-485 isolation by LC-MS and SDS-PAGE. The observed molecular weight (38,500 Da) determined by LC-MS matched the expected theoretical value of the protein construct, with no evidence of cysteine oxidation or other post-translational modifications. A representative SDS-PAGE gel image of the protein used in this analysis is shown in the inset. **b** Steady-state binding of BU170, BU3-12, and EPI-7386 to AR101-485 measured by MST. The inset compares the calculated apparent dissociation constants *K*_D_ for compound binding. Data presented as mean ± SEM (*n* = 3). **c** SPR sensorgram of BU3-12 binding to His-AR101-485 covalently coupled to an NTA chip. The data fit the kinetic model shown in Fig. 6d. Arrows indicate injection points of BU3-12. The experiment was performed in triplicate, and a representative run is shown. **d** Reaction scheme of analyte A binding to two transient protein states B1 and B2, applied in the SPR kinetic model. **e** Calculated kinetic parameters obtained from fitting the SPR kinetic model shown in Fig. 6d. **f** Component analysis of the fitted SPR model showing accumulation of the second bound state AB2 despite the slower BU3-12 binding to B2 state than to the B1 state. **g** MS/MS data showing the fragmentation of BU170 modified (top) and BU3-12 modified (middle) GCVPEPGAAVAASK. Fragment ions display localization of the modification on C129. The DMSO control shows only the unmodified form of the peptide (bottom). **h** A competition binding curve showing inhibition of fluoromone binding to recombinant AR-LBD by dihydrotestosterone (DHT), R1881, bicalutamide (BIC) and enzalutamide (ENZA) which were all positive controls. BU130 and EPI-7386 also competed for the AR-LBD at concentrations similar to those mediating biological activity in the other assays (i.e., see Fig. 1g)

To evaluate the in vitro binding of ARTADIs to AR-TAD, a recombinant AR101-485 fragment was expressed, isolated and purified (Fig. 6a). This fragment includes the AF-1 region. Microscale thermophoresis (MST) analysis demonstrated direct binding of BU3-12, BU170 and masofaniten (EPI-7386) to AR101-485 (Fig. 6b). The apparent dissociation constants (K_D_) for these interactions were determined to be 0.3 nM, 1.3 nM, and 0.8 nM, respectively, indicating tight-affinity binding. To further confirm the strong binding of the most effective BU3-12, surface plasmon resonance (SPR) was performed. Sensorgrams revealed a low equilibration rate and very slow dissociation (Fig. 6c), features typical of tight binding. The best-fitting model provided by the Biacore evaluation software was initially a heterogeneous two-site competition model, which was further refined to a two-state transient binding model (Fig. 6d) to account for the conformational plasticity of the intrinsically disordered AR-TAD and for dependence on binding time dissociation rate (data not shown). The calculated kinetic and equilibrium parameters (Fig. 6e) indicated the presence of both low-micromolar (*K*_D1_ = 5.71 μM) and low-nanomolar (*K*_D2_ = 3.35 nM) binding states of His-AR101-485, suggesting weaker affinities to BU3-12 compared to MST data (*K*_D_ = 0.3 nM). Component analysis (Fig. 6f) revealed a rapid transition (*k*_+3_ = 3.79 × 10⁷ s^−1^) from the initial bound state (AB1) to a second state (AB2) characterized by a much slower dissociation rate. This transition was induced by BU3-12 and occurred fully despite the initial AB1/AB2 ratio corresponding to the *R*_max1_/*R*_max2_ values 0.7, and was only slightly reversible (*K*_3_ = 1.15 × 10^−6^). Because the association rate for the first state (B1) was higher than for the second (B2), the overall apparent affinity was determined by the most statistically confident *k*_a1_ and *k*_d2_ rate constants, resulting in a *K*_Dapp_ of 0.2 nM (Fig. 6e) which is consistent with the MST-derived apparent *K*_D_ of 0.3 nM (Fig. 6b).

Some of the tested ARTADIs were expected to act as covalent binders to ARTAD, as they contain the same chlorohydrin group as EPI-001.^4^ Under the concentrations and incubation times used for MST and SPR analyses, no covalent modifications were detected by LC–MS analysis. However, upon extending the incubation time to 20 h and increasing the concentrations of the binding partners, a small fraction of AR101-485 exhibited additional mass species (503 Da and 553 Da for BU3-12 and BU170, respectively), which are consistent with the molecular masses of the corresponding ARTADIs (541 Da and 591 Da for BU3-12 and BU170, respectively). These covalent modifications were used to localize the site of direct binding on AR101-485, so LC-MS/MS sequencing was performed on the BU170 and BU3-12 modified form, as well as the DMSO control. With sequence coverage for AR101-485 between 80-90% for each analysis, modification at cysteine 129 was detected for both the BU170 (Fig. 6g top) and BU3-12 (Fig. 6g middle) mass shifts in their respective samples, with no matching spectra in the DMSO control condition (Fig. 6g bottom). Additional validation of the modification is observed in the isotope distribution of the fragment ions, with c-terminal y-type ions showing normal peptide-like isotope distributions, while b-type ions containing the modified cysteine show characteristic isotope patterns of chlorine containing molecules; consistent with the presence of 2, and only two, chlorine atoms. We propose that a basic nitrogen in the AR-TAD binding site deprotonates the chlorohydrin secondary alcohols in BU3-12 and BU170 to form alkoxides that displace the adjacent chlorine atom to form epoxides. The epoxides are attacked at the primary carbon by C129 thiol to form the covalent modifications.^4^

To determine if any of the ARTADIs affected ligand-binding to the isolated AR-LBD, we employed fluorescence polarization. BU130 competed with fluoromone with a similar IC_50_ as enzalutamide (4.4 μM vs 3.5 μM, respectively) (Fig. 6h). Masofaniten had an IC_50_ of 9.4 μM, whereas both BU3-12 and BU170 did not substantially compete with ligand for AR-LBD. These data reveal that both masofaniten and BU130 that lack chlorohydrin have off-target effects on isolated AR-LBD that may partially explain the differences on gene expression and their ability to block the cell cycle compared to other ARTADIs. Together these binding data support that BU3-12 and BU170 both with chorohydrins are selective for AR-TAD which are consistent with gene expression data and functional assays.

### Impact of ARTADIs on AR protein-protein interactions

**Fig. 7:**
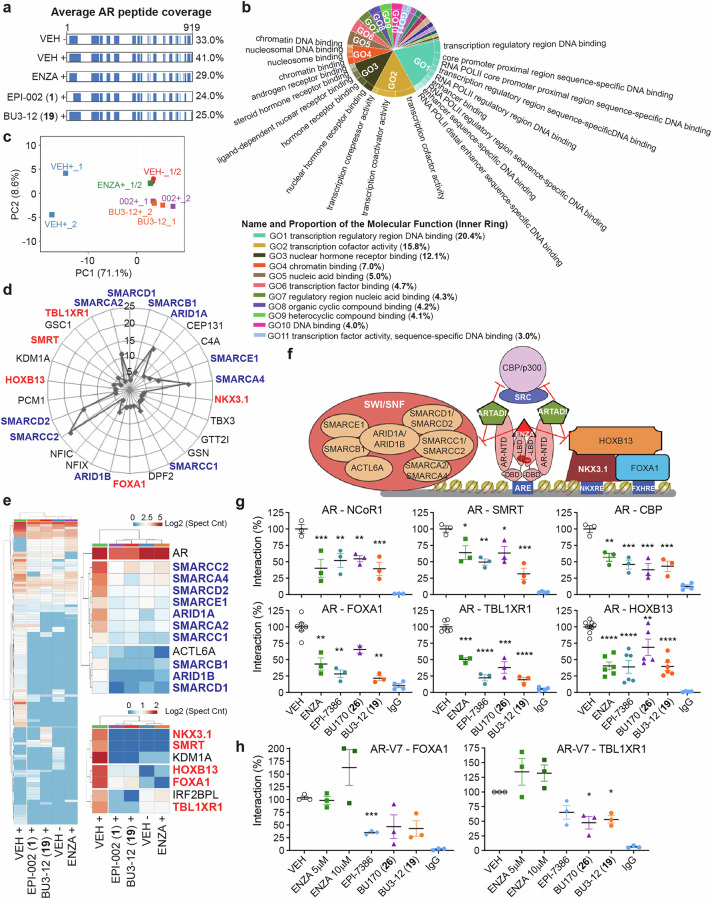
Impact of ARTADIs on AR protein-protein interactions. **a** AR RIME was performed in androgen-stimulated (+) LNCaP cells treated with DMSO vehicle, enzalutamide (5 µM), EPI-002/ralaniten (35 µM) or BU3-12 (5 µM). Average peptide coverage of AR bait protein for each treatment (*n* = 2). **b** A CirGO image plot of clustered gene ontology terms (molecular function) which were associated with AR interactions in vehicle treated samples stimulated with R1881. **c** PCA plot based upon 97 AR-specific interactions. **d** Radar chart showing top AR-binding partners in vehicle treated samples stimulated with R1881. Members of the SWI-SNF chromatin remodeling complex (blue) and known AR co-regulatory partners (red) are shown. **e** Heatmap showing selected AR-interactions following treatment with inhibitors. **f** Cartoon showing proposed model of ARTADI and enzalutamide antagonism of AR coregulatory proteins. **g** Plots showing AR interactions using PLA and normalized to vehicle control. LNCaP cells were pretreated with vehicle, ENZA (5 µM), EPI-7386 (5 µM), BU170 (10 µM), BU3-12 (5 µM), or normal rabbit and mouse IgG for 1 h and stimulated with R1881 (1 nM) for 2 h. **h** Plots showing AR-V7 interactions using PLA and normalized to vehicle control. VCaP-ENZR cells were treated with vehicle, ENZA (5 µM), EPI-7386 (5 µM), BU170 (10 µM), BU3-12 (5 µM) or normal rabbit and mouse IgG for 3 h. Bars represented the mean ± SEM (*n* = 3–6 independent experiments). *p < 0.05, **p < 0.01, ***p < 0.001, ****p < 0.0001. See Supplementary Fig. 6

The gene- and cell-specific sensitivities measured in response to AR-inhibitors likely involve differences in the repertoire and concentrations of AR-interacting-proteins and the abilities of a specific inhibitor to block such interactions. To reveal differences in protein complexes differentially disrupted by enzalutamide versus ARTADIs, rapid immunoprecipitation mass spectrometry of endogenous protein (RIME) was employed using full-length AR as bait in androgen-stimulated LNCaP cells. AR coverage was in the range of 24% to 41% depending upon the inhibitor (Fig. 7a). Interactions with AR included proteins involved in transcription as expected as well as many well-characterized AR binding partners (Fig. 7b). PCA shows clustering of all inhibitors suggesting that many of the interactions that were inhibited were common amongst enzalutamide and ARTADIs (Fig. 7c). Known interactions with AR were blocked by enzalutamide and ARTADIs that included members of the SWI/SNF complex, NKX3.1, SMRT, KDM1A, HOXB13, FOXA1, TBL1XR1 and IRF2BPL (Fig. 7d–f). The proximity ligation assay validated RIME data and revealed that BU3-12 was in general the superior ARTADI, and it outperformed enzalutamide; i.e., SMRT (68% vs 36% inhibition), TBL1XR1 (81% vs 50% inhibition), and FOXA1 (78% vs 57% inhibition) (Fig. 7g and Supplementary Fig. 6a). Repeating using VCaP-ENZR cells that have high expression of AR-V7,^9^ revealed that enzalutamide did not inhibit interactions between FOXA1 or TBL1XR1 with AR-V7 contrary to ARTADIs (Fig. 7h, Supplementary Fig. 6b).

### Potential of ARTADIs as a treatment for eugonadal prostate cancer patients

First generation antiandrogens are not FDA-approved for eugonadal prostate cancer patients due to a lack of survival benefit compared to castration for reasons that possibly included: antiandrogens elevating circulating levels of testosterone and estrogen^32–35^; and their inability to effectively compete with androgen for AR-LBD.^4,16,36^ Only recently has enzalutamide been approved for treatment in eugonadal prostate cancer patients.

**Fig. 8:**
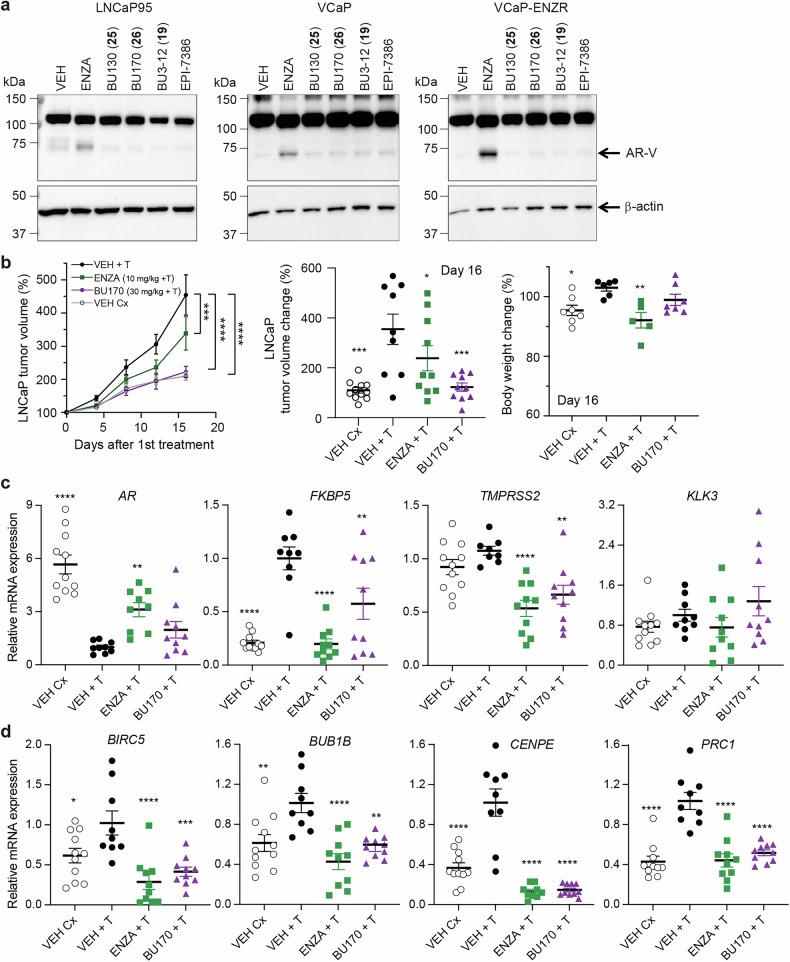
Potential of ARTADIs as a treatment for eugonadal prostate cancer patients. **a** Full-length AR (~110 kDa) and AR-V (~75 kDa) protein levels in androgen-stimulated cells treated with inhibitors. β-actin was used as a loading control. A representative blot is provided from *n* = 3–5 independent experiments. **b** Left panel - temporal tumor growth of subcutaneous LNCaP xenografts in NSG mice that were all castrated (Cx) and then supplemented with a time-release testosterone (T) pellet. Daily gavage treatments of vehicle with no T-pellet (VEH Cx); vehicle plus T-pellet (VEH + T); enzalutamide plus T-pellet (ENZA + T); and BU170 plus T-pellet (BU170 + T). Middle panel shows the final tumor volumes at day 16. Right panel shows final body weight changes for each mouse. **c** Transcript levels of *AR* and androgen-regulated genes, and **d** cell cycle genes that were normalized to the housekeeping gene *SDHA* using RNA isolated from xenografts shown in **b**. Data is normalized to VEH + T control and presented as mean ± SEM, and analyzed by one-way ANOVA with Dunnet’s correction. *p < 0.05; **p < 0.01; ***p < 0.001; ****p < 0.0001. See Supplementary Fig. 7b, c

Caveats of enzalutamide and castration are their propensities to induce expression of *AR3*/AR-V7 thereby leading to resistance.^14,21–23,37^ Consistent with others, here in the presence of androgen, enzalutamide induced levels of AR-V7 protein which did not occur with ARTADIs (Fig. 8a and Supplementary Fig. 7a). This phenotypic difference plus the fact that ARTADIs still maintain efficacy in the presence of high concentrations of androgen,^4,16^ directed us to test the feasibility of an ARTADI as a therapeutic against androgen-sensitive prostate cancer in the presence of androgen. The LNCaP xenograft was used in castrated hosts supplemented with a testosterone pellet to reduce complications of elevated testicular androgen induced by enzalutamide and to maintain levels of free testosterone due to a lack of sex hormone-binding globulin in mice. BU170 was selected based upon potency and the fact that it does not compete with androgen for AR-LBD (Fig. 6h).

In the presence of androgen, BU170 reduced the growth of LNCaP tumors to comparable volumes measured from castrated hosts without testosterone pellets (Fig. 8b). Contrary to the outstanding efficacy of enzalutamide under castrate conditions (Fig. 2a, b), in the presence of androgen it was inferior to BU170 (Fig. 8b). In spite of this modest efficacy and elevated expression of *AR* in the presence of testosterone (Fig. 8c), enzalutamide still inhibited expression of *FKBP5* and *TMPRSS2* as did BU170 (Fig. 8c, VEH + T vs ENZA and vs BU170). Contrary to serum levels of PSA as being one of the best biomarkers in oncology, *KLK3* mRNA is a poor indicator of blocking AR transcriptional activity and not a reliable prognostic marker.^4,38^ Consistent with these reports, neither enzalutamide, BU170 nor castration significantly decreased levels of *KLK3* transcript. Levels of mRNA of the highly related gene, *KLK2*, were also not impacted (Supplementary Fig. 7b). Whereas levels of mRNAs for *RHOU* and *NKX3.1* were both decreased with enzalutamide, but not with BU170. Enzalutamide and BU170 elevated levels of *AR3* (AR-V7) mRNA. Of the genes associated with E2F targets, G2M checkpoint, and mitotic spindle that were measured, castration, enzalutamide and BU170 all significantly decreased levels of androgen-induced expression of *BIRC5*, *BUB1B*, *CENPE*, and *PRC1* (Fig. 8d). Both enzalutamide and BU170 also decreased levels of *KIF15*, whereas gene-specific responses were observed for other genes with expression of some genes being more sensitive to enzalutamide (*CCNA2*, *CDC20*, *KIF18B*, *KIF2C*) and others being more sensitive to BU170 (*CCNB1*, *CCNB2*, *CCND1*) (Supplementary Fig. 7c).

Pharmacokinetic studies revealed substantial improvement in sustaining blood levels of BU170 compared to EPI-001. Eight hours after a single i.v. dose of 20 mg/kg body weight of BU170, blood levels dropped from 12.6 μM to 7.4 μM (Supplementary Fig. 7d), whereas previously we reported that 8 h after a single i.v. dose of 50 mg/kg body weight of EPI-001 the blood levels dropped from 61.2 μM to 2.4 μM.^4^

## Discussion

IDRs of transcription factors impact regulation of transcription by mechanisms that include: facilitation of transcriptional condensates at super-enhancers; contributions to DNA binding by flanking DNA-binding domains to impact base-dependent minor groove interactions; interacting with many different proteins to form multiple complexes; and directing the transcription factor to specific binding sites in the genome.^39,40^

Intrinsic disorder provides the unique property of multivalent binding which may encompass short, low affinity motifs that support interactions with a broad network of interacting partners which yield multiple points of contact that can add up to a stable tight-affinity interaction.^41^ Consistent with this, the long and disordered AR-TAD interacts with approximately 300 proteins^42^ and ARTADIs that were discovered here have extremely tight binding that challenges the affinities of clinical drugs that bind the folded AR-LBD. These multiple binding sites within ARTAD impacted by multivalent binding interactions may, or may not, occur in a stepwise fashion. Possible perturbations of any single binding event could have consequences on the conformations of the ensemble and alter the properties of interacting proteins and/or the potency of a small molecule. Thus the binding of a small molecule to an intrinsically disordered TAD is highly complex compared to the lock-and-key model for folded proteins. Drug development to IDRs was considered unfeasible due to skepticism that specificity and potency could be achieved due to the multitude of conformations and dynamic nature of the drug target.^43^The emergence of drugs in clinical trials that bind to the IDR of AR such as ralaniten-acetate and masofaniten has provided support that drugs can be developed to IDRs that have low toxicity and have shown signs of efficacy in Phase 1 clinical trials.

Here we are the first to report that minor modifications in the chemical structures of ARTADIs impact potency against AR-regulated genes in a gene- and cell-specific manner with unique consequences on cellular pathways. Regulation of transcription is dependent upon affinity of the transcription factor for DNA-binding sites and protein-protein interactions which are influenced by post-translational modifications and cellular levels of expression. The measured in vitro affinities were substantially tighter than the cellular IC₅₀ values reported for the same ARTADIs—a discrepancy frequently observed for many drugs and their targets.^44^ This difference may be attributed to unequal intracellular and extracellular distribution of the hydrophobic ARTADIs (BU170 CLogP = 2.83; BU3-12 CLogP = 3.88). Such properties likely result in compound sequestration, such as differential partitioning between hydrophobic membranes and hydrophilic cytosol/nucleus environments, leading to lower effective local concentrations in the nucleus than the total applied concentration. Additional factors, such as off-target binding, ongoing AR turnover (synthesis and degradation), may also contribute to reduced apparent binding efficiency in cells. Nevertheless, the tight in vitro affinities observed strongly support a direct interaction between the tested compounds and the AR-NTD in cells and in vivo, highlighting the relevance of this binding event for inhibition of AR functions. Despite the relatively close K_D_ values the observed relative differences in binding affinity are significant and reflect meaningful structural distinctions among the compounds. Notably, BU3-12 exhibited approximately two-fold stronger affinity than EPI-7386 and over four-fold stronger affinity than BU170. These differences correlate well with the compounds’ inhibitory effects on AR-V7 transcriptional activity (Fig. 3a), supporting a shared mechanism of AR inhibition via binding AR-TAD.

The observed covalent binding of BU3-12 and BU170 to the AR-TAD suggests a more complex mechanism of AR inhibition that involves at least two distinct steps. The first step is a rapid, non-covalent interaction with AR-TAD, as demonstrated by MST and SPR analyses. This interaction can be partial, likely due to the conformational flexibility of AR-TAD; according to SPR data, approximately half of the total AR101-485 population is capable of binding BU3-12. The second step is a very slow covalent attachment to Cys129, as revealed by MS analysis. This covalent modification renders the interaction irreversible and stabilizes the AR in an inhibited conformation. In addition, covalent attachment shifts the conformational equilibrium of unbound AR-TAD toward states that are competent for ARTADI binding. Provided that the half-time of covalent bond formation is shorter than the half-life of AR (i.e., the rate of covalent binding exceeds the rate of receptor synthesis and degradation), this mechanism would result in a gradual accumulation of inhibited AR within cells over the course of several days. The fact that both ARTADIs form covalent bonds exclusively with the same cysteine residue provides strong evidence for the high selectivity of this interaction, as none of the other nine cysteine residues present in the AR101-485 fragment were modified. This observation further suggests that Cys129, and likely neighboring amino acid residues, directly participate in the initial non-covalent step of BU3-12 and BU170 binding.

One of the most striking differences was that BU3-12 induced G2 arrest in LNCaP95 cells, while other ARTADIs caused G1 arrest. Subsequent gene expression analysis indicated that there was a concurrent bias towards BU3-12 inhibiting cell cycle genes which were either unaffected or inhibited to a much lower degree with other ARTADIs. Interestingly, in LNCaP cells this effect was lost, and BU3-12 blocked cells in G1 as with the other ARTADIs. Together these highlight the gene- and cell-specific differences seen by slight chemical alterations of ARTADIs.

These gene- and cell specificities may be due to differing DNA-binding affinities depending upon the gene and concentrations and affinities of interacting proteins for full-length AR compared to AR-V7. In addition to possible differences in post-translational modification pathways between different cells, the cellular concentrations of full-length AR and AR-V7 proteins are highly variable across prostate cancer tissues and cells. Such differences in levels of AR have been proposed to contribute, at least in part, to differences in the number of genes regulated by full-length AR as well as those regulated by AR-V7.^20^ In addition, AR-V7 regulated genes have DNA-binding sites enriched near the transcriptional start sites of genes unlike those for full-length AR.^20^ The DNA-binding sites for AR-V7 varies depending on whether expression of the gene is induced or repressed by AR-V7. Induced genes are enriched in consensus motifs for androgen-response element (ARE)/glucocorticoid response element while repressed genes are enriched with FOX family binding-sites unlike full-length AR which has DNA-binding sites of regulated genes enriched with both AREs and FOX sites regardless of whether its expression is induced or repressed.^20^ We show that FOXA1 interaction with full-length AR is inhibited by ARTADIs and enzalutamide yet enzalutamide failed to block interaction of FOXA1 with AR-V7 unlike ARTADIs. These differences between AR-LBD inhibitors and ARTADIs on protein-protein interactions would contribute to the overall cistrome and transcriptome mediated by each inhibitor to alter the cellular pathways that could ultimately impact proliferation, survival and invasiveness of a cancer cell. Indeed, we revealed that ARTADIs were unique from enzalutamide in altering expression of genes within the cellular pathways involved in E2F targets, G2M checkpoint and mitotic spindle thereby attenuating core protumorigenic mechanisms.

The transcriptional activity of androgen-induced full-length AR is dependent upon interdomain interactions between its N-terminal TAD and C-terminal LBD (called N/C interactions).^45^ Here we found that full-length AR was more sensitive to ARTADIs compared to AR-V7 as shown for reporter-genes and survival of androgen-stimulated LNCaP cells versus LNCaP95 cells. Others have also reported this trend with a ralaniten-like analogues with differing structural motifs.^11^ This suggests that the AR-LBD modulates the conformational ensemble(s) of the N-terminal TAD possibly due to androgen-induced N/C interactions. Both blocking N/C interaction^11^ and ARTADIs^11,12,46^ attenuate the formation of AR transcriptionally active condensates thereby highlighting modulation of liquid-liquid phase separation as a plausible mechanism of action of ARTADIs.

The improved potency of ARTADIs against androgen-induced full-length AR compared to AR-V7, together with ARTADIs not inducing levels of AR-V7 protein and not competing with ligand for the AR-LBD led to the testing of BU170 efficacy against hormone-sensitive prostate cancer tumor growth in the presence of testosterone. These studies revealed that ARTADIs are more efficacious than enzalutamide under these conditions. Past failures of first-generation antiandrogens as a monotherapy in eugonadal prostate cancer patients have been revisited with second-generation antiandrogens to provide patients with an option that could prevent the considerable adverse effects of castration such as osteoporosis. This patient population may be optimal for treatment with ARTADIs with possible superior efficacy to enzalutamide as well as potential maintenance of a phenotype that would retain sensitivity to androgen-deprivation therapy, antiandrogens and abiraterone-acetate based upon differences in the mechanisms of action.

## Materials and methods

### Reagents

R1881 was purchased from AK Scientific (Union City, Ca), Enzalutamide was purchased from Selleckchem (Houston, TX). BU compounds were synthesized by us. Masofaniten was purchased from WuXi (Shanghai, China).

### Cell lines and tissue culture

LNCaP cells were obtained from Dr. Lelund Chung (Cedars Sinai Medical Center, Los Angeles, CA), LNCaP95 cells from Dr. Jun Luo (Johns Hopkins University, Baltimore, MD) and PC3 and VCaP cells were obtained from American Type Culture Collection (Manassas, VA). LNCaP and PC3 cells were authenticated by short tandem repeat analysis and tested for Mycoplasma by DDC Medical and VCaP and LNCaP95 cells by The Centre of Applied Genomics, Sick Kids Hospital, Toronto, Ontario. The cell lines are regularly tested for Mycoplasma (VenorTMGeM Mycoplasma detection kit, Sigma- Aldrich). LNCaP cells were maintained in RPMI with 5% FBS, LNCaP95 in RPMI (Invitrogen, Carlsbad, CA) with 10% Charcoal stripped serum (CSS), PC3 in DMEM with 5% FBS and VCaP in DMEM (Sigma-Aldrich, St Louis, MO) with 10% FBS, 2 mM L-glutamine and 1 mM Sodium pyruvate. All cell lines were cultured in a 37 °C humidified incubator containing 5% CO_2_. LNCaP95-D3 and VCaP-ENZR cells have previously been described by us^1,2^.

### Transfections and reporter gene assays

PSA (6.1kb)-luciferase reporter^3^ was provided by Dr. J.-T. Hsieh at the University of Southwestern Medical Center, Dallas, TX. PB-luciferase reporter was obtained from Dr. Matusik, Nashville, Tennessee; ARR3-tk-luc reporter was developed by us^4^. The *AR3*/AR-V7 expression plasmid and V7BS_3_-luciferase plasmid, which contains three tandem repeats of an *AR3*-specific promoter element of the *UBE2C* gene^5^, was a gift from Dr. Stephen Plymate (University of Washington).

### Transcriptional activity of full-length AR

LNCaP cells were seeded at 5.5 × 10^4^ cells per well into 24-well plates. To test AR-FL transcription activity, the following day, cells were transfected with the reporter plasmids PSA(6.1kb)-Luc, PB-Luc and ARR3-Luc in serum-free and red phenol-free medium, using Fugene HD Transfection agent (Promega). For IC_50_ calculations, cells were transfected for 18 h, followed by treatment with different concentrations of compounds or vehicle for 1 h before the addition of 1 nM R1881. Cells were lysed after 24 h and analyzed for luciferase activities. Raw data were normalized to protein concentrations. At least three independent experiments were performed in triplicate wells.

### Transcriptional activity of AR-V7

LNCaP cells were seeded at 5.5 × 10^4^ cells per well into 24-well plates and co-transfected with the V7BS3-Luc reporter and the expression plasmid AR-V7 (6.25 ng/well). Eighteen hours after transfection, the cells were treated with different concentrations of each compound and incubated for another 24 h. Luciferase activities were measured and normalized to protein concentration.

### Cell cycle analyses and γH2AX expression

LNCaP and LNCaP95 cells were seeded into 100 mm plates in 5% FBS and 60 mm dishes in 1.5% CSS, respectively. The cells were incubated for 24 or 48 h and labeled with BrdU (1 µM final concentration) for 2 h before being harvested and fixed in 70% ethanol. For γH2AX analysis, cells were incubated first with γH2AX rabbit antibody (Ser139, #9718 Cell Signaling, 1:500) followed by AlexaFluor 488 anti-rabbit (1:200). For cell cycle analysis, cells were incubated with BrdU-FITC antibody (BD Biosciences, Franklin Lakes, New Jersey, USA). DNA was stained with 7-AAD. Data were acquired using a FACS Calibur or LSR Fortessa (BD Biosciences) and analyzed using FlowJo software ver.10.3 (Ashland, Oregon, USA).

### Clonogenic assay

LNCaP cells (2000 cells/100 mm dish), LNCaP95 (1000 cells/well into 6-well plate) and PC3 cells (600 cells/well into 6-well plates), respectively, were treated with different concentrations of compounds. And allowed to form colonies for an additional 21 days for LNCaP95 cells, while LNCaP and PC3 for 14 days and 8 days, respectively. Colonies were fixed with 4% paraformaldehyde and stained with 0.1% (PC3 cells) or 0.01% (LNCaP and LNCaP95 cells) crystal violet for 15 min. Colonies containing more than 50 cells were counted using GelCount v.1.2.1.0 (Oxford-Optronix, Abingdon, United Kingdom).

### Gene expression

LNCaP cells (125,000 cells/well) were plated in 6-well plates. Cells were serum-starved for 24 h prior to pre-treatment with DMSO vehicle, enzalutamide, BU130, BU170, ralaniten (EPI-002), BU3-12 or masofaniten (EPI-7386) for 1 h before stimulation with 1 nM R1881 for 24 h in serum-free media. LNCaP95 (250,000 cells/well) and VCaP (500,000 cells/well) cells were plated in 6-well plates for 24 h prior to treatment with DMSO vehicle, enzalutamide, BU130, BU170, EPI-002 or EPI-7386 for 48 h in media containing 1.5% charcoal-stripped serum. For gene expression of xenografts, tumors were flash frozen in liquid nitrogen immediately following excision. Approximately 300 mg of tumors was homogenized in 1 mL TRIzol (Invitrogen) using a FastPrep-24 tissue homogenizer (MP Biomedicals). Total RNA was isolated using pure link RNA isolation kit (ThermoFisher Scientific), cleaned using the DNAse I kit (Millipore Sigma) and reverse transcribed with high-capacity RNA to cDNA kit (ThermoFisher Scientific) according to the manufacturer’s protocols. Diluted cDNA and PowerUp SYBR Green qPCR Master Mix (Invitrogen) were combined with gene-specific primers. Transcript quantification was performed on a QuantStudio 6 Flex Real-Time PCR system (ThermoFisher Scientific). Expression levels were normalized to levels of *SDHA* transcript. Primers are described in Supplementary Table S1.

### RNA-seq and GSEA analysis

Total RNA derived from LNCaP or LNCaP95 cells was purified using the pure link RNA isolation kit (ThermoFisher Scientific). Cells were treated as for qRT-PCR experiments. RNA quality was assessed prior to sequencing on the Agilent Bioanalyzer 2100 using the RNA 6000 Nano Kit (Agilent). RNA-sequencing was performed using the Illumina NovaSeq 6000 platform run as paired-end reads of 150 base pairs with a depth of 25 million fragments/library. Raw sequences were mapped via STAR to the human reference genome GRCh38. Differential gene expression analysis was performed using R package DEseq2 (1.42.1) and the Benjamini–Hochberg correction was applied to all *p* values. Gene Set Enrichment Analysis (GSEA) was carried out using software available from the Broad Institute (GSEA version 7.0; http://software.broadinstitute.org/gsea/msigdb/index.jsp). The difference in the expression levels between vehicle and drug treatment for each gene was analyzed based on the Molecular Signatures Database set H (Hallmark gene sets; h.all.v7.1.symbols.gmt). Those enrichment gene sets revealed by GSEA as exhibiting a nominal *p* < 0.05 and FDR < 0.05 were considered to indicate a statistically significant difference. Hierarchical clustering was performed on normalized enrichment scores (NES) for each sample, and heatmap and PCA plots were generated using ClustVis software (https://biit.cs.ut.ee/clustvis/#general). RNA-seq data were submitted to GEO (accession numbers GSE271953, LNCaP; GSE271954, LNCaP95) and are provided in Supplementary Document S2 (LNCaP) and Supplementary Document S3 (LNCaP95).

### Recombinant protein expression and purification

The pET28a vector encoding the AR101-485 fragment with an N-terminal hexahistidine-SUMO tag was transformed into *E. coli* SHuffle electrocompetent cells, which were grown in Terrific Broth (TB) at 37 °C until an OD_600_ of 0.8–0.9 was reached. Protein expression was induced with IPTG, and the culture temperature was reduced to 23 °C for 16–18 h. Cells were harvested by centrifugation and stored frozen until further processing. His-SUMO-AR101-485 was isolated using a denaturation-refolding protocol^6^. Cell pellets were resuspended in lysis buffer containing 8 M urea, 50 mM HEPES pH 7.4, 500 mM NaCl, 10% glycerol, 1 mM TCEP, and 5 mM MgCl_2_, and lysed by sonication (Branson 250, 50% amplitude). Cell debris was removed by centrifugation at 200,000 × *g* for 1 h. The supernatant was applied to a nickel-affinity column (Ni-NTA agarose, GoldBio) equilibrated in 8 M urea, 50 mM HEPES pH 7.4, 300 mM NaCl, 10% glycerol, 1 mM TCEP, and 10 mM imidazole. After washing with three column volumes of the same buffer, the bound protein was eluted with 200 mM imidazole. The eluate was dialyzed (12–14 kDa MWCO, Spectra/Por) stepwise against buffers containing 4, 2, and 0 M urea to allow gradual refolding. Following dialysis, the His-SUMO tag was cleaved with SUMO protease. The cleaved tag and protease were removed by reverse IMAC, and the AR101-485 fragment was eluted with 30–40 mM imidazole in buffer containing 25 mM HEPES pH 7.4, 50 mM NaCl, 10% glycerol, 1 mM TCEP, and 2.5 mM MgCl_2_. Final purification was performed using ion-exchange chromatography on a Mono Q 5/50 GL column equilibrated in the same buffer (without imidazole). Target fractions were eluted using a step gradient from 4.5% to 9% of 1 M NaCl, pooled, and concentrated to the desired final concentration. The expected molecular weight 38,500 Da of the isolated protein was confirmed with SDS-PAGE and liquid chromatography mass spectrometry analysis at Simon Fraser University (Fig. 6a). The AR101-485 fragment containing an N-terminal decahistidine tag for SPR experiments was generated using the previously provided vector. Expression and purification followed the established protocol, except that the His-tag was not cleaved and reverse IMAC was omitted. Target fractions were eluted with a linear gradient up to 1 M NaCl and subsequently pooled. The expected molecular weight of the His-AR101-485 fragment (41.2 kDa) was confirmed by SDS-PAGE.

### Steady state binding analysis using microscale thermophoresis (MST)

MST was employed to measure the binding affinity (*K*_D_) between the AR101-485 fragment and selected compounds. The MST method was conducted as previously described^7^, using a Monolith NT.115 Pico instrument (NanoTemper Technologies). The AR101–485 protein was fluorescently labeled with the NT-647 dye by amine coupling, targeting lysine residues and/or the N-terminus. Labeling was performed using the Monolith NT Protein Labeling Kit according to the manufacturer’s protocol. Labeling efficiency, determined by absorbance measurements at 280 and 650 nm, ranged from 1/1 to 2/1 (protein/dye ratio). Test compounds were serially diluted from 100 µM to ~7 pM. The labeled protein was added at a final concentration of 0.1–0.5 nM, depending on the optimal fluorescence signal. Samples were loaded into standard silica capillaries (NanoTemper) and analyzed at 37 °C in assay buffer containing 20 mM HEPES (pH 7.4), 150 mM NaCl, 2.5 mM MgCl_2_, 1 mM TCEP, 0.1% DMSO, and 0.1% Pluronic F-127. Measurements were performed using 20% LED power and 40% IR-laser power; additional measurements were acquired at 60% IR-laser power for comparison. Data analysis was performed using NanoTemper Analysis software, version 2.3.

### Kinetic binding analysis using surface plasmon resonance (SPR)

A Biacore T200 instrument (Cytiva) was used to evaluate the interaction between His-AR101-485 and BU3-12. The His-AR101-485 fragment was immobilized on an NTA sensor chip to ~2500 RU and further stabilized by amine coupling. Experiments were carried out at 37 °C in PBS (pH 7.4) supplemented with 2.5 mM MgCl₂ and 0.1% (v/v) DMSO, with solvent correction applied. Multi-injection, single-cycle sensorgrams were double-referenced, and kinetic parameters were determined using Biacore™ T200 BIA evaluation software. The data best fit a heterogeneous ligand model incorporating an additional transition between two binding states (Fig. 6d). The mean total *R*_max_ of three experiments was 17.8 RU, which is ~54% of the theoretical *R*_max_ (32.7 RU), indicating that approximately half of the immobilized His-AR101-485 was active.

### Covalent binding detection using mass spectrometry (MS) analysis

AR101-485 (15 µM**)** was incubated with compounds BU3-12 and BU170 (150 µM) for 20 h at 37 °C in the buffer containing 20 mM HEPES (pH 7.4), 150 mM NaCl, 2.5 mM MgCl_2_, 1 mM TCEP, 0.15% DMSO, 0.5% glycerol. The control sample was incubated in the same conditions without any inhibitors. Then the buffer was changed to 5 mM (NH_3_)HCO_3_ buffer with micro Bio-Spin P-30 chromatography column. The level of possible protein degradation was monitored with SDS-PAGE and was negligible compared to non-incubated samples. Samples were reduced and alkalized simultaneously with 10 mM TCEP and 40mM CAA, digested with trypsin overnight, and cleaned up via StageTip (PMID: 17703201). The resulting peptides were reconstituted in 0.1% formic acid, 0.5% acetonitrile in water, and 20 ng were loaded onto a Bruker TimsTOF Pro2 mass spectrometer, coupled to a NanoElute 2 with online separation on an Aurora Series Gen3 CSI column (25 cm × 75 µm, 1.7 µm C18; Ion Opticks, Parkville, Victoria, Australia). Data was acquired using standard nano-LC and MS (DDA-PASEF) methods. Raw data were searched against the sequence of AR101-485 using Byonic (v4.0.12; Protein Metrics Inc.) with 20 ppm/30 ppm precursor/product tolerance, 1% false discovery rate, variable (*C*) carbamidomethylation, (*Q*) deamidation and (*M*) oxidation. In addition, an unbiased open search was added for any amino acid with a mass delta from 552 to 554 Da for BU170, or from 502 to 504 Da for BU3-12. Data are shared on the ProteomeXchange server with the identifier: PXD071971.

### Fluorescent polarization assay

PolarScreen™ Androgen Receptor Competitor Assay kit (Invitrogen) was used according to the manufacturer’s protocol. Serial dilution was done for each compound in DMSO. Fluorescence polarization at excitation wavelength 470 nm and emission at 530 nm were measured in Greiner 384 black clear-bottomed plates using Infinite M1000 (TECAN, Austria, GmbH).

### RIME

RIME AR (cat# 39311; Active Motif) was performed by Active Motif Epigenetic Services (Carlsbad, CA, USA). 2.5 × 10^6^ LNCaP cells were plated in 15 cm plate, serum starved for 24 h, and treated with vehicle, 10 μM Enzalutamide, 35 μM EPI-002 and 5 μM BU3-12 for 16 h before adding R1881 to a final concentration of 1 nM for 3 h. The cells were fixed according to the manufacturer’s instructions (RIME cell fixation protocol, Active Motif). A total of 1 × 10^8^ cells were prepared per treatment with two biological replicates per treatment. Analysis was performed by Active Motif and results are presented in Supplementary Document S4. All protein interactions that were present in IgG controls were excluded, as were interactions that were not present in both positive control samples, and *p* < 0.01 was used as a cut-off. Heatmap showing average sample spectral counts of both biological replicates and PCA analysis was completed using ClustVis software (https://biit.cs.ut.ee/clustvis/#general). Arc plot of significant Gene Ontology terms (Molecular Function; p<0.05) was generated using CirGO software (v.2.0).

### Proximity ligation assay (PLA)

LNCaP cells were grown on 8-well chamber slides and were serum-starved in phenol-red-free RPMI for 48 h. Then cells were treated with 5 µM ENZA, 5 µM EPI-7386, 10 µM BU170, and 5 µM BU3-12. After 1 h of treatment, cells were stimulated with 1 nM R1881 for 2 h, followed by fixing and permeabilizing using 4% paraformaldehyde and 0.1% Triton X-100. For PLA analysis, LNCaP cells were blocked with Duolink Blocking Solution and incubated with anti-AR (EMD Millipore), anti-FOXA1 (Abcam), anti-HoxB13 (Santa Cruz Biotechnology), anti-TBL1XR1 (Santa Cruz Biotechnology), anti-NCoR1 (Invitrogen), anti-SMRT (Invitrogen), anti-CBP (Santa Cruz Biotechnology) and normal mouse and rabbit IgG (Cell Signaling). VCaP -ENZR cells (50,000/well) were plated in 8-well chamber slides. The next day, the media was changed to DMEM + 1.5% DCC for 24 h before treating with 5 µM ENZ, 5 µM EPI-7386, 10 µM BU-170, and 5 µM BU3-12, for 3 h. The cells were fixed using 4% paraformaldehyde and permeabilized with 0.1% Triton x-100. PLA was performed using the Duolink PLA kit and incubated with Anti-ARV-7 (RevMaB, Burlingame, CA), anti-FOXA1 (Abcam), anti-TBL1XR1 (Santa Cruz Biotechnology). Protein–protein interactions in both cell lines were analyzed using Duolink-based in situ PLA Red or Orange kit (Sigma-Aldrich), according to the manufacturer’s instructions. Fluorescence signals were captured using a Zeiss microscope, quantified in ImageJ, and normalized to the corresponding counts of DAPI-stained nuclei.

### Western blot analyses

LNCaP95 cells were plated in 6-well plates (250,000 cells per well) in culture medium (RPMI-1640 + 10% CSS). The next day, cells were pre-treated with DMSO (VEH), BU130 (5 μM) and 10 μM of all remaining compounds in RPMI-1640 medium containing 1.5% CSS for 1 h, and then treated with 1 nM R1881 for 2 days prior to collecting cell lysate. BU130 was toxic at higher doses using these cell culture conditions. VCaP and VCaP-ENZR cells were plated in 12-well plates (200,000 cells per well) in culture medium (DMEM + 10% FBS + L-Glutamine + Sodium pyruvate and 0.35% D-glucose). The next day, the culture medium was changed to DMEM medium containing 1.5% CSS. After 1 day, the cells were pre-treated with indicated inhibitors (all at 5 μM except BU130, which was 2.5 μM) or control vehicle in DMEM medium containing 1.5% CSS for 1 h, and then treated with 1nM R1881 for 2 days prior to collecting cell lysate. Upon collecting cell lysate, cells were rinsed in ice-cold PBS and lysed in lysis buffer containing 50 mM Tris, pH 7.4, 150 mM NaCl, 0.5% NP-40, 0.5% sodium deoxycholate, 0.1% SDS, protease inhibitors (Roche cOmplete EDTA-free protease inhibitor cocktail; MilliporeSigma), phosphatase inhibitors (Roche PhosStop; Millipore Sigma), and nuclease (Benzonase; MilliporeSigma). Clear whole-cell lysate was collected after centrifugation. Protein concentration was quantified using bicinchoninic acid assay (Pierce BCA Protein Assay kit; Thermo Scientific). Equal amounts of lysate were loaded and resolved on SDS-PAGE, and were transferred to nitrocellulose membranes for Western blot analyses. Antibodies were anti-AR (rabbit monoclonal, clone ER179(2); Abcam Cat# ab108341) and anti-β-actin (mouse monoclonal, clone AC-15; MillioreSigma Cat# A5441). Secondary antibodies conjugated with HRP were used (goat polyclonal anti-rabbit IgG and goat polyclonal anti-mouse IgG from Cell Signaling Technology Cat# 7074 and Cat# 7076). Enhanced ECL (Clarity Max Western ECL Substrate; Bio-Rad) was applied for chemiluminescence detection using ChemiDoc XRS+ Imaging System (Bio-Rad). Protein bands were quantified and calculated by Image Lab 5.1 and GraphPad Prism 8.

### Animal studies

All experiments involving animals conform to the relevant regulatory and ethical standards and were approved by the University of British Columbia Animal Care Committee (A22-0079). The duration of dosing varied depending on either IRB (Institutional Review Board) compliance and/or limitation of compound availability. Metacam (1 mg/kg, 0.05 mL/10 g of body weight) was administered subcutaneously prior to any surgery. Animals were anesthetized with isoflurane and euthanized by CO_2_. Six- to 8-week-old male NSG mice were maintained in the Animal Care Facility at the British Columbia Cancer Research Centre. LNCaP cells (5 × 10^6^) were injected into 6- to 8-week-old male mice in 1:1 volume of Matrigel (Corning). The mice were castrated when the tumors reached 100 mm^3^. Daily dosing started 7 days after castration. The tumors were harvested 24 h after the last dose. For LNCaP95-D3 tumors, the mice were castrated 15 days prior to being injected with LNCaP95-D3 cells (2 × 10^6^). Daily dosing by gavage started when the tumor volumes reached 50–90 mm^3^. Xenografts were harvested 3 h after the last dose. VCaP-ENZR cells (2.5 × 10^6^) were injected into 6- to 8-week-old male NSG mice in 1:1 ratio with Matrigel. The mice were castrated when the tumor volumes reached 150–200 mm^3^. Daily oral dosing started 7 days after castration for 14 days and xenografts were harvested 2 h after the last dose. For LNCaP xenografts plus testosterone pellet (Innovative Research of America, Sarasota, Florida), the testosterone pellet (7.5 mg, 60 day release) was implanted subQ when the tumors were palpable and then mice were castrated when the tumors reached 50 mm^3^. The mice were treated 21 days. The tumors were harvested 24 h after the last dose for LNCaP.

### Stability of BU170 in vivo

Each NRG mouse was administered by tail vein i.v. a single dose of 20 mg/kg body weight of BU170 in vehicle (30% PEG; 8% DMSO; 62% H_2_O). Blood samples were collected at 5  min, 30 min, 2 h, and 8 h. Immediately upon blood collection 19.0 µg of BU106 in 10 µL of DMSO was added to each sample to provide an internal control for extraction. The resulting blood serum obtained for each sample was extracted with EtOAc (2 × 400 µL). In each case, the combined EtOAc extracts were washed with brine (400 µL). Each EtOAc extract was then dried and taken up in 100 µL DMSO. Forty µL of each sample was then quantified utilizing C_18_ reversed-phase HPLC analytical method that was first developed using a Waters 1525 Binary HPLC Pump attached to a Waters 2998 Photodiode Array Detector using a InertSustain, 5 µm, 25 × 1 cm column. An isocratic system with 52:48 MeCN/H_2_O as eluent for 38 min followed by a linear gradient to 85/15 MeCN/H_2_O over an additional 22 min at a flow rate of 2 mL/min with UV detection at 197.8 and 275.0 nm was used. A reliable detection limit for both BU170 and BU106 was determined to be ~35 ng per 10 µL injection with a linear response above this level up to the highest concentration tested at ~10 µg per 10 µL injection.

### Statistics

A one- or two-way ANOVA statistical test was used to determine significance for all comparisons unless specifically stated otherwise (Graphpad Prism version 9.1.0). *p* value corrections were applied for all multiple comparisons (Tukey or Dunnett as appropriate), and *p* < 0.05 was considered statistically significant.

### Ethics approval

All animal experiments conform to the relevant regulatory and ethical standards and guidelines for the care and use of laboratory animals. Experimental procedures were approved by the University of British Columbia Animal Care Committee (certificate A22-0079, April 14, 2025).

## Supplementary information

S1 Supplementary Materials for Drugging the intrinsically disordered transactivation domain of androgen receptor

S2 RNA-Seq LN95 supplementary data

S3 RNA-Seq LNCaP supplementary data

S4 RIME supplementary data

S5 Synthesis and Compound Characterization

## Data Availability

All data in the manuscript are available in the main text and its supplementary information. RNA-sequencing data are available in the GEO repository under accession numbers GSE271953 for LNCaP and GSE271954 for LNCaP95. Mass spectrometry data is shared on the ProteomeXchange server with the identifier: PXD071971.

## References

[1] Holehouse, A. S. & Kragelund, B. B. The molecular basis for cellular function of intrinsically disordered protein regions. *Nat. Rev. Mol. Cell Biol.* **25**, 187–211 (2024). 37957331 10.1038/s41580-023-00673-0 PMC11459374

[2] Staller, M. V. Transcription factors perform a 2-step search of the nucleus. *Genetics* **222**, iyac111 (2022). 35939561 10.1093/genetics/iyac111 PMC9526044

[3] Andersen, R. J. et al. Regression of castrate-recurrent prostate cancer by a small-molecule inhibitor of the amino-terminus domain of the androgen receptor. *Cancer Cell* **17**, 535–546 (2010). 20541699 10.1016/j.ccr.2010.04.027

[4] Myung, J. K. et al. An androgen receptor N-terminal domain antagonist for treating prostate cancer. *J. Clin. Invest* **123**, 2948–2960 (2013). 23722902 10.1172/JCI66398 PMC3696543

[5] De Mol, E. et al. EPI-001, a compound active against castration-resistant prostate cancer, targets transactivation unit 5 of the androgen receptor. *ACS Chem. Biol.* **11**, 2499–2505 (2016). 27356095 10.1021/acschembio.6b00182 PMC5027137

[6] Sadar, M. D. et al. Sintokamides A to E, chlorinated peptides from the sponge Dysidea sp. that inhibit transactivation of the N-terminus of the androgen receptor in prostate cancer cells. *Org. Lett.* **10**, 4947–4950 (2008). 18834139 10.1021/ol802021w

[7] Banuelos, C. A. et al. Sintokamide A is a novel antagonist of androgen receptor that uniquely binds activation function-1 in its amino-terminal domain. *J. Biol. Chem.* **291**, 22231–22243 (2016). 27576691 10.1074/jbc.M116.734475 PMC5064002

[8] Banuelos, C. A. et al. Ralaniten sensitizes enzalutamide-resistant prostate cancer to ionizing radiation in prostate cancer cells that express androgen receptor splice variants. *Cancers* **12**, 1991 (2020). 32708219 10.3390/cancers12071991 PMC7409302

[9] Hirayama, Y., Tam, T., Jian, K., Andersen, R. J. & Sadar, M. D. Combination therapy with androgen receptor N-terminal domain antagonist EPI-7170 and enzalutamide yields synergistic activity in AR-V7-positive prostate cancer. *Mol. Oncol.* **14**, 2455–2470 (2020). 32734688 10.1002/1878-0261.12770 PMC7530779

[10] Yan, L. et al. Structure-activity relationships for the marine natural product sintokamides: androgen receptor N-terminus antagonists of interest for treatment of metastatic castration-resistant prostate cancer. *J. Nat. Prod.* **84**, 797–813 (2021). 33124806 10.1021/acs.jnatprod.0c00921 PMC8802828

[11] Xie, J. et al. Targeting androgen receptor phase separation to overcome antiandrogen resistance. *Nat. Chem. Biol.* **18**, 1341–1350 (2022). 36229685 10.1038/s41589-022-01151-y

[12] Basu, S. et al. Rational optimization of a transcription factor activation domain inhibitor. *Nat. Struct. Mol. Biol.* **30**, 1958–1969 (2023). 38049566 10.1038/s41594-023-01159-5 PMC10716049

[13] Kotamarti, S., Armstrong, A. J., Polascik, T. J. & Moul, J. W. Molecular mechanisms of castrate-resistant prostate cancer. *Urol. Clin. North Am.* **49**, 615–626 (2022). 36309418 10.1016/j.ucl.2022.07.005

[14] Zheng, Z. et al. The crucial role of AR-V7 in enzalutamide-resistance of castration-resistant prostate cancer. *Cancers* **14**, 4877 (2022). 36230800 10.3390/cancers14194877 PMC9563243

[15] Obst, J. K. et al. Revealing metabolic liabilities of ralaniten to enhance novel androgen receptor targeted therapies. *ACS Pharm. Transl. Sci.* **2**, 453–467 (2019). 10.1021/acsptsci.9b00065 PMC7088963 32259077

[16] Imamura, Y. et al. An imaging agent to detect androgen receptor and its active splice variants in prostate cancer. *JCI Insight* **1**, e87850 (2016). 27525313 10.1172/jci.insight.87850 PMC4980083

[17] Leung, J. K. et al. Pin1 inhibition improves the efficacy of ralaniten compounds that bind to the N-terminal domain of androgen receptor. *Commun. Biol.* **4**, 381 (2021). 33753863 10.1038/s42003-021-01927-3 PMC7985297

[18] Tien, A. H. & Sadar, M. D. Cyclin-dependent kinase 4/6 inhibitor palbociclib in combination with ralaniten analogs for the treatment of androgen receptor-positive prostate and breast cancers. *Mol. Cancer Ther.* **21**, 294–309 (2022). 34815359 10.1158/1535-7163.MCT-21-0411 PMC8828702

[19] Gritsina, G., Gao, W. Q. & Yu, J. Transcriptional repression by androgen receptor: roles in castration-resistant prostate cancer. *Asian J. Androl.* **21**, 215–223 (2019). 30950412 10.4103/aja.aja_19_19 PMC6498738

[20] Basil, P. et al. Cistrome and transcriptome analysis identifies unique androgen receptor (AR) and AR-V7 splice variant chromatin binding and transcriptional activities. *Sci. Rep.* **12**, 5351 (2022). 35354884 10.1038/s41598-022-09371-x PMC8969163

[21] Hornberg, E. et al. Expression of androgen receptor splice variants in prostate cancer bone metastases is associated with castration-resistance and short survival. *PLoS ONE* **6**, e19059 (2011). 21552559 10.1371/journal.pone.0019059 PMC3084247

[22] Sharp, A. et al. Androgen receptor splice variant-7 expression emerges with castration resistance in prostate cancer. *J. Clin. Invest* **129**, 192–208 (2019). 30334814 10.1172/JCI122819 PMC6307949

[23] Hu, R. et al. Distinct transcriptional programs mediated by the ligand-dependent full-length androgen receptor and its splice variants in castration-resistant prostate cancer. *Cancer Res.* **72**, 3457–3462 (2012). 22710436 10.1158/0008-5472.CAN-11-3892 PMC3415705

[24] Cao, B. et al. Androgen receptor splice variants activating the full-length receptor in mediating resistance to androgen-directed therapy. *Oncotarget* **5**, 1646–1656 (2014). 24722067 10.18632/oncotarget.1802 PMC4039237

[25] Xu, D. et al. Androgen receptor splice variants dimerize to transactivate target genes. *Cancer Res.* **75**, 3663–3671 (2015). 26060018 10.1158/0008-5472.CAN-15-0381 PMC4558376

[26] Yang, Y. C. et al. Targeting androgen receptor activation function-1 with epi to overcome resistance mechanisms in castration-resistant prostate cancer. *Clin. Cancer Res.* **22**, 4466–4477 (2016). 27140928 10.1158/1078-0432.CCR-15-2901 PMC5010454

[27] Leung, J. K., Tam, T., Wang, J. & Sadar, M. D. Isolation and characterization of castration-resistant prostate cancer LNCaP95 clones. *Hum. Cell* **34**, 211–218 (2021). 32954481 10.1007/s13577-020-00435-6 PMC8693726

[28] Miller, K. J. et al. A compendium of androgen receptor variant 7 target genes and their role in castration resistant prostate cancer. *Front. Oncol.* **13**, 1129140 (2023). 36937454 10.3389/fonc.2023.1129140 PMC10014620

[29] Liu, W. et al. Homozygous deletions and recurrent amplifications implicate new genes involved in prostate cancer. *Neoplasia* **10**, 897–907 (2008). 18670647 10.1593/neo.08428 PMC2481576

[30] Makkonen, H., Kauhanen, M., Jaaskelainen, T. & Palvimo, J. J. Androgen receptor amplification is reflected in the transcriptional responses of vertebral-cancer of the prostate cells. *Mol. Cell Endocrinol.* **331**, 57–65 (2011). 20728506 10.1016/j.mce.2010.08.008

[31] Murthy, S. et al. Role of androgen receptor in progression of LNCaP prostate cancer cells from G1 to S phase. *PLoS ONE* **8**, e56692 (2013). 23437213 10.1371/journal.pone.0056692 PMC3577675

[32] Verhelst, J. et al. Endocrine profiles during administration of the new non-steroidal anti-androgen Casodex in prostate cancer. *Clin. Endocrinol.* **41**, 525–530 (1994). 10.1111/j.1365-2265.1994.tb02585.x 7525125

[33] Tyrrell, C. J. et al. A randomised comparison of ‘Casodex’ (bicalutamide) 150 mg monotherapy versus castration in the treatment of metastatic and locally advanced prostate cancer. *Eur. Urol.* **33**, 447–456 (1998). 9643663 10.1159/000019634

[34] Smith, M. R. et al. Bicalutamide monotherapy versus leuprolide monotherapy for prostate cancer: effects on bone mineral density and body composition. *J. Clin. Oncol.* **22**, 2546–2553 (2004). 15226323 10.1200/JCO.2004.01.174

[35] Tombal, B. et al. Enzalutamide monotherapy in hormone-naive prostate cancer: primary analysis of an open-label, single-arm, phase 2 study. *Lancet Oncol.* **15**, 592–600 (2014). 24739897 10.1016/S1470-2045(14)70129-9

[36] Kunath, F. et al. Non-steroidal antiandrogen monotherapy compared with luteinizing hormone-releasing hormone agonists or surgical castration monotherapy for advanced prostate cancer: a Cochrane systematic review. *BJU Int.* **116**, 30–36 (2015). 25523493 10.1111/bju.13026

[37] Sun, S. et al. Castration resistance in human prostate cancer is conferred by a frequently occurring androgen receptor splice variant. *J. Clin. Investig.* **120**, 2715–2730 (2010). 20644256 10.1172/JCI41824 PMC2912187

[38] Mostaghel, E. A. et al. Intraprostatic androgens and androgen-regulated gene expression persist after testosterone suppression: therapeutic implications for castration-resistant prostate cancer. *Cancer Res.* **67**, 5033–5041 (2007). 17510436 10.1158/0008-5472.CAN-06-3332

[39] Brodsky, S., Jana, T. & Barkai, N. Order through disorder: the role of intrinsically disordered regions in transcription factor binding specificity. *Curr. Opin. Struct. Biol.* **71**, 110–115 (2021). 34303077 10.1016/j.sbi.2021.06.011

[40] Cermakova, K. & Hodges, H. C. Interaction modules that impart specificity to disordered protein. *Trends Biochem. Sci.* **48**, 477–490 (2023). 36754681 10.1016/j.tibs.2023.01.004 PMC10106370

[41] Sipko, E. L., Chappell, G. F. & Berlow, R. B. Multivalency emerges as a common feature of intrinsically disordered protein interactions. *Curr. Opin. Struct. Biol.* **84**, 102742 (2024). 38096754 10.1016/j.sbi.2023.102742

[42] Culig, Z. & Puhr, M. Androgen receptor-interacting proteins in prostate cancer development and therapy resistance. *Am. J. Pathol.* **194**, 324–334 (2024). 38104650 10.1016/j.ajpath.2023.12.003 PMC12178391

[43] Lazar, T., Connor, A., DeLisle, C. F., Burger, V. & Tompa, P. Targeting protein disorder: the next hurdle in drug discovery. *Nat. Rev. Drug Discov.* **24**, 743–763 (2025). 40490488 10.1038/s41573-025-01220-6

[44] Kotani, N. & Ito, K. Translatability of in vitro potency to clinical efficacious exposure: A retrospective analysis of FDA-approved targeted small molecule oncology drugs. *Clin. Transl. Sci.* **16**, 1359–1368 (2023). 37173825 10.1111/cts.13532 PMC10432864

[45] Askew, E. B., Minges, J. T., Hnat, A. T. & Wilson, E. M. Structural features discriminate androgen receptor N/C terminal and coactivator interactions. *Mol. Cell Endocrinol.* **348**, 403–410 (2012). 21664945 10.1016/j.mce.2011.03.026 PMC3199032

[46] Zhang, F. et al. Dynamic phase separation of the androgen receptor and its coactivators key to regulate gene expression. *Nucleic Acids Res.* **51**, 99–116 (2023). 36535377 10.1093/nar/gkac1158 PMC9841400

