# Supplementary material for: Drugging the intrinsically disordered transactivation domain of androgen receptor
Source: Signal Transduct Target Ther. 2026 Apr 28;11:157. doi: 10.1038/s41392-026-02642-3 (PMC13121470; doi:10.1038/s41392-026-02642-3)
Supplement: Supplementary file 1 — S1 Supplementary Materials for Drugging the intrinsically disordered transactivation domain of androgen receptor [file 41392_2026_2642_MOESM1_ESM.docx]

Supplementary Materials for

**Drugging the intrinsically disordered transactivation domain of androgen receptor**

Jon K. Obst^1^, Carmen A. Banuelos^1^, Kunzhong Jian^1,2^, Amy H. Tien^1^, Oleksandr A. Shkrabak^3^, Jun Wang^1^, Nasrin R. Mawji^1^, Teresa Tam^1^, Marija Vuckovic^3^, David E. Williams^2^, Jason C. Rogalski^4^, Xiaojing Yuan^4^, Natalie C.J. Strynadka^3,*^, Raymond J. Andersen^2,*^, Marianne D. Sadar^1, 5,*^

Correspondence to: msadar@bcgsc.ca

**This WORD file includes:**

- Supplementary Table 1. Primer sequences.
- Supplementary Document 1. Supplementary Figs. 1–7.
- Supplementary Material. Supplementary Fig 8. Uncut gels

**Other Supplementary Materials for this manuscript include the following:**

- Supplementary Document 2. Excel file containing RNA-seq data from LNCaP95 experiment, related to Fig. 4, Supplementary Figs. 2 and 3.
- Supplementary Document 3. Excel file containing RNA-seq data from LNCaP experiment, related to Fig. 5, Supplementary Figs. 4 and 5.
- Supplementary Document 4. Excel file containing RIME data, related to Fig. 7.
- Supplementary Document 5. Word document containing the Synthesis and Compound Characterization of small molecules.


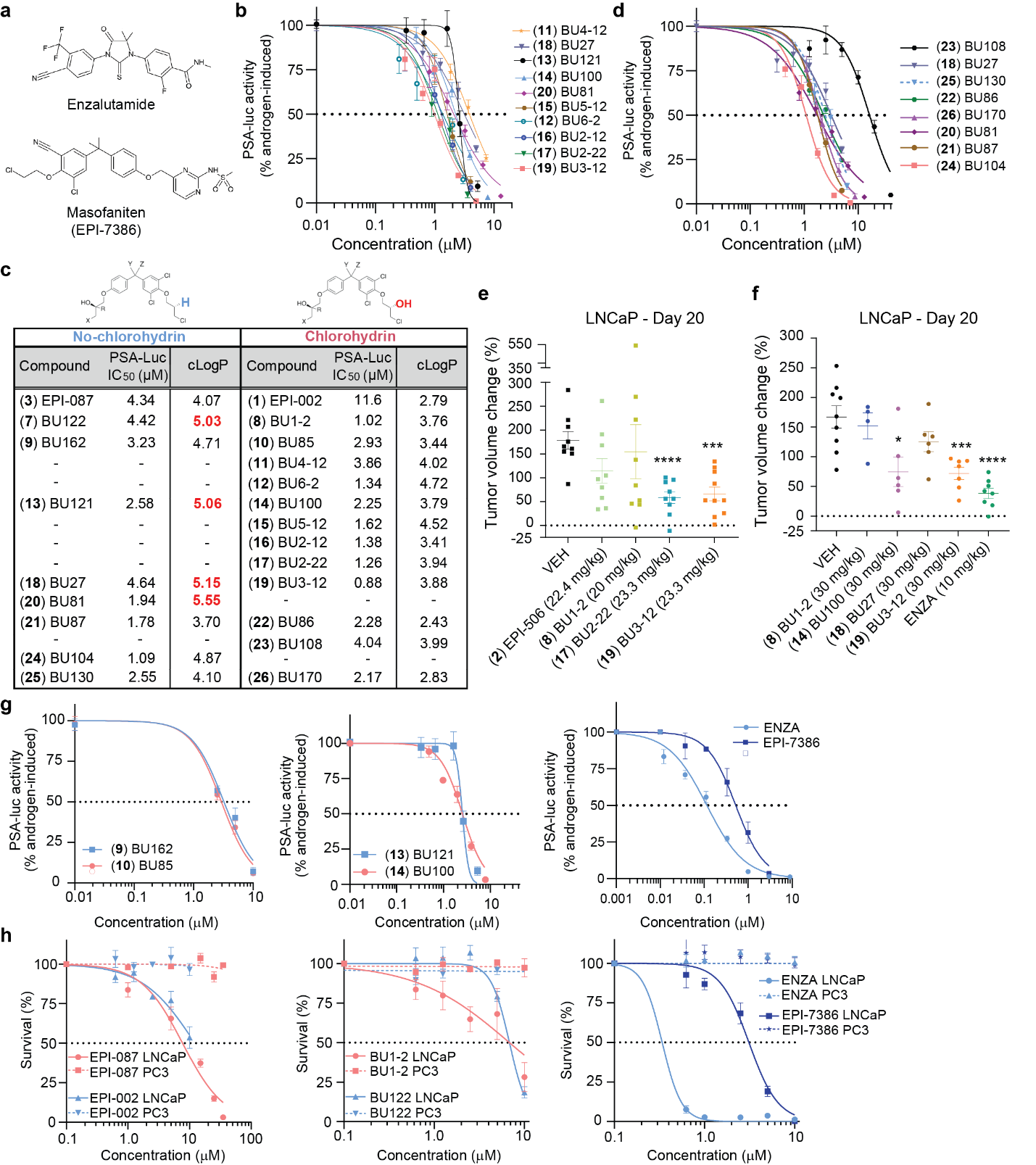


Figure. S1.

**Improving the potency and efficacy of compounds**. **a** Structures of enzalutamide and masofaniten. **b** Dose response curves of compounds designed to improve metabolically stability on androgen-induced PSA-luciferase activity in LNCaP cells. **c** Table showing IC_50_s of compounds to inhibit androgen-induced PSA-luciferase activity in LNCaP cells and their cLogP values. Compounds in the same row differ only by the presence or absence of a chlorohydrin as shown in cartoon above the table. Dashed lines in same row indicate no matched compound was used. **d** Dose response curves of fluorinated compounds on androgen-induced PSA-luciferase activity in LNCaP cells. **e** Tumor volume change of LNCaP xenografts from castrated hosts after 20 days of oral dosing compared to the starting volume. Mice were treated with equimolar concentrations of EPI-506, BU1-2, BU2-22 or BU3-12. **f** Tumor volume change of LNCaP xenografts from castrated hosts after 20 days of oral dosing compared to the starting volume. Mice were treated with equal doses of 30 mg/kg body weight of BU1-2, BU100, BU27, and BU3-12 compared to enzalutamide (10 mg/kg body weight). **g** Dose response curves of inhibition of androgen-induced PSA-luciferase activity for paired compounds which differed only in the presence (red) or absence (blue) of the chlorohydrin functional group. Enzalutamide (ENZA) and masofaniten (EPI-7386) are included as controls for comparison. **h** Cell survival curves of the colony formation assay with LNCaP (solid lines) or PC3 (hashed lines). Cells exposed to serial dilutions of enzalutamide (ENZA), masofaniten (EPI-7386) or paired compounds with (red) or without (blue) chlorohydrin functional group. Data represented as mean ± SEM.


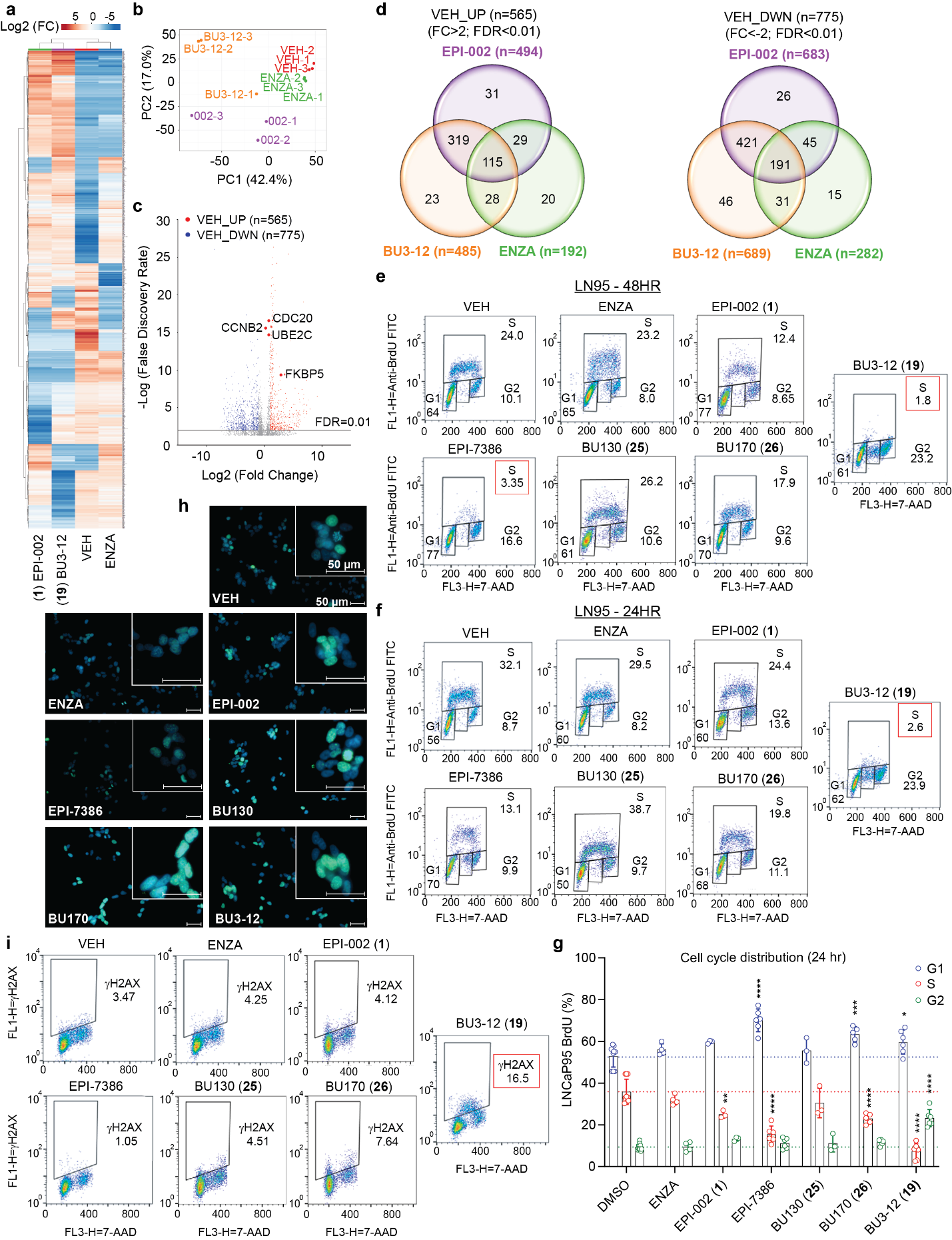


Figure. S2.

**Impact of ARTADIs on cell cycle and DNA damage in AR-V7-dependent LNCaP95 cells. a** Heatmap of normalized signal intensity following RNA-seq transcriptional profiling of LNCaP95 cells treated with 35 μM EPI-002/ralaniten, 5 μM BU3-12, DMSO vehicle (VEH-), or 5 μM enzalutamide (ENZA) for 48 hrs in steroid-depleted media. The mean expression value of three independent experiments is presented. Fold-change was calculated using normalized signal intensity averaged across all samples. **b** PCA plot representing each sample localized based upon transcript counts for 3,275 genes. **c** Volcano plot depicting genes which displayed a 2-fold change in levels of expression and FDR<0.01 in control samples (DMSO, VEH). 565 and 775 genes were defined as DMSO_UP and DMSO_DWN respectively. **d** Venn diagrams showing the ability of EPI-002, BU3-12 and/or enzalutamide to repress genes positively associated with DMSO treatment (left) or derepress negatively expressed genes associated with DMSO (VEH) treatment by 2-fold or greater. **e** Representative bivariate plots showing cell cycle distribution of LNCaP95 cells treated with DMSO (VEH), enzalutamide (ENZA, 5 µM), EPI-002 (35 µM), EPI-7386 (5µM), BU130 (5 µM), BU170 (10 µM at 48 hrs and 5 μM at 24 hrs), and BU3-12 (5 µM) for 48 hrs, or **f** 24 hrs and stained with BrdU-FITC and 7-AAD. **g** Bar plot of averaged cell cycle distribution of LNCaP95 cells exposed for 24 hrs to DMSO (VEH), enzalutamide (ENZA, 5 μM), EPI-002 (35 μM), EPI-7386 (5 μM), BU130 (5 μM), BU170 (5 μM), and BU3-12 (5 μM). Bars represent the mean ± SEM of n=3 independent experiments. * p<0.05, ** p<0.01, *** p<0.001, **** p <0.0001; ANOVA test with Tukey correction. **h** γH2AX staining in LNCaP95 cells treated with DMSO (VEH), enzalutamide (ENZA, 5 µM), EPI-002 (35 µM), EPI-7386 (5 µM), BU130 (5 µM), BU170 (10 µM), and BU3-12 (5 µM) for 48 hrs. Nuclei were co-stained with DAPI. **i** Representative plots of FACS analysis for γH2AX. Fixed single LNCaP95 cells were treated as in **h** for 48h and stained with γH2AX antibody and 7-AAD.


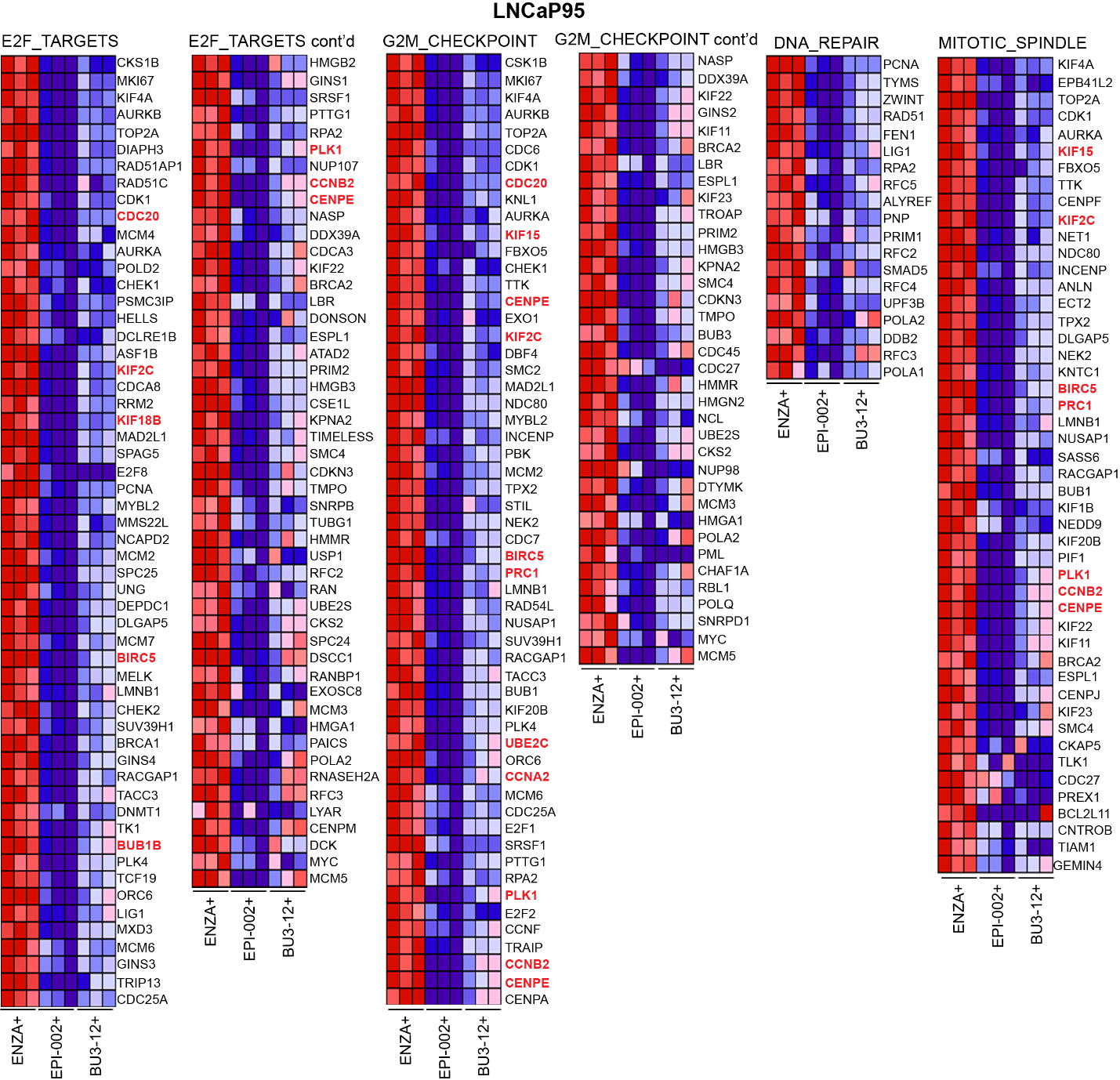


Figure. S3.

**Unique gene expression profiles associated with ARTADIs compared to enzalutamide in LNCaP95 cells.** Heatmap showing relative expression of core enriched genes contributing to the leading edge in GSEA enrichment plots shown in Fig. 4b. Genes highlighted in red were chosen for further qRT-PCR validation.


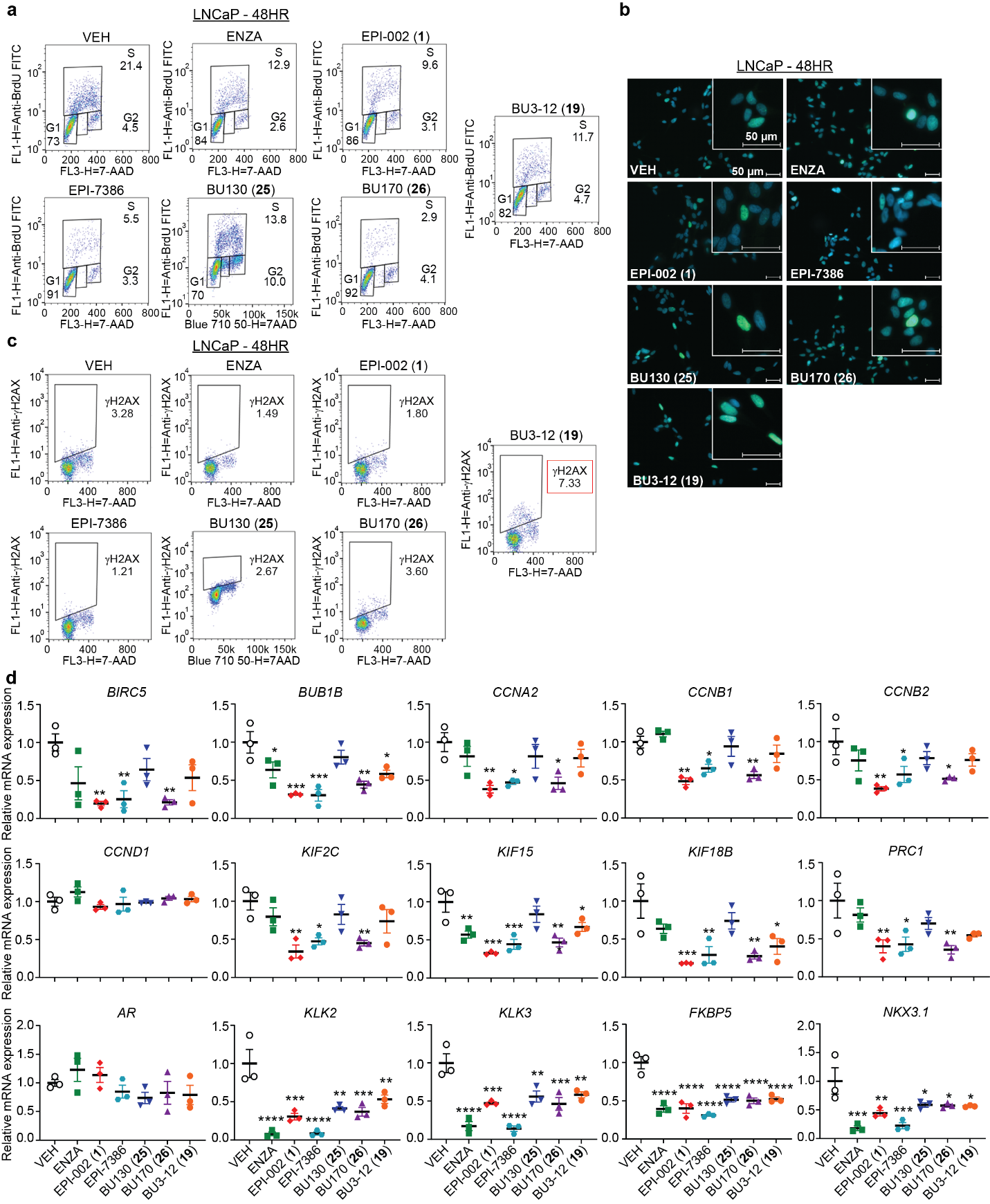


Figure. S4.

**Impact of ARTADIs on cell cycle and DNA damage in androgen-stimulated cells that are dependent on full-length AR. a** Representative bivariant plots showing cell cycle of LNCaP cells treated for 48 h with DMSO (VEH), enzalutamide (ENZA, 10 µM), EPI-002/ralaniten (35 µM), EPI-7386 (10 µM), BU130 (5 µM), BU170 (10 µM), and BU3-12 (10µM). **b** Immunostaining for γH2AX of LNCaP cells exposed to same concentration of compounds and for the same amount of time as in **a**. **c** Bivariate representative experiment of γH2AX expression of LNCaP cells treated as in **a**. **d** Levels of mRNA transcript of cell cycle genes *BIRC5*, *BUB1B*, *CCNA2*, *CCNB1*, *CCNB2*, *CCND1*, *KIF2C*, *KIF15*, *KIF18B*, *PRC1* and androgen-induced genes *KLK2*, *KLK3*, *FKBP5* and *NKX3.1* plus *AR* all normalized to housekeeping gene *SDHA* from LNCaP cells treated DMSO (VEH), enzalutamide (5 µM), EPI-002 (35 µM), EPI-7386 (5 µM) BU130 (5 µM), BU170 (10 µM), or BU3-12 (5 µM), for 48 hrs in media supplemented with 5% FBS. Data is normalized to DMSO (VEH) control and presented as mean ± SEM, and analyzed by one-way ANOVA with Dunnet’s test applied post hoc to correct for multiple comparisons (n=3 independent experiments). *p<0.05; ** p<0.01; *** p<0.001; ****p<0.0001.


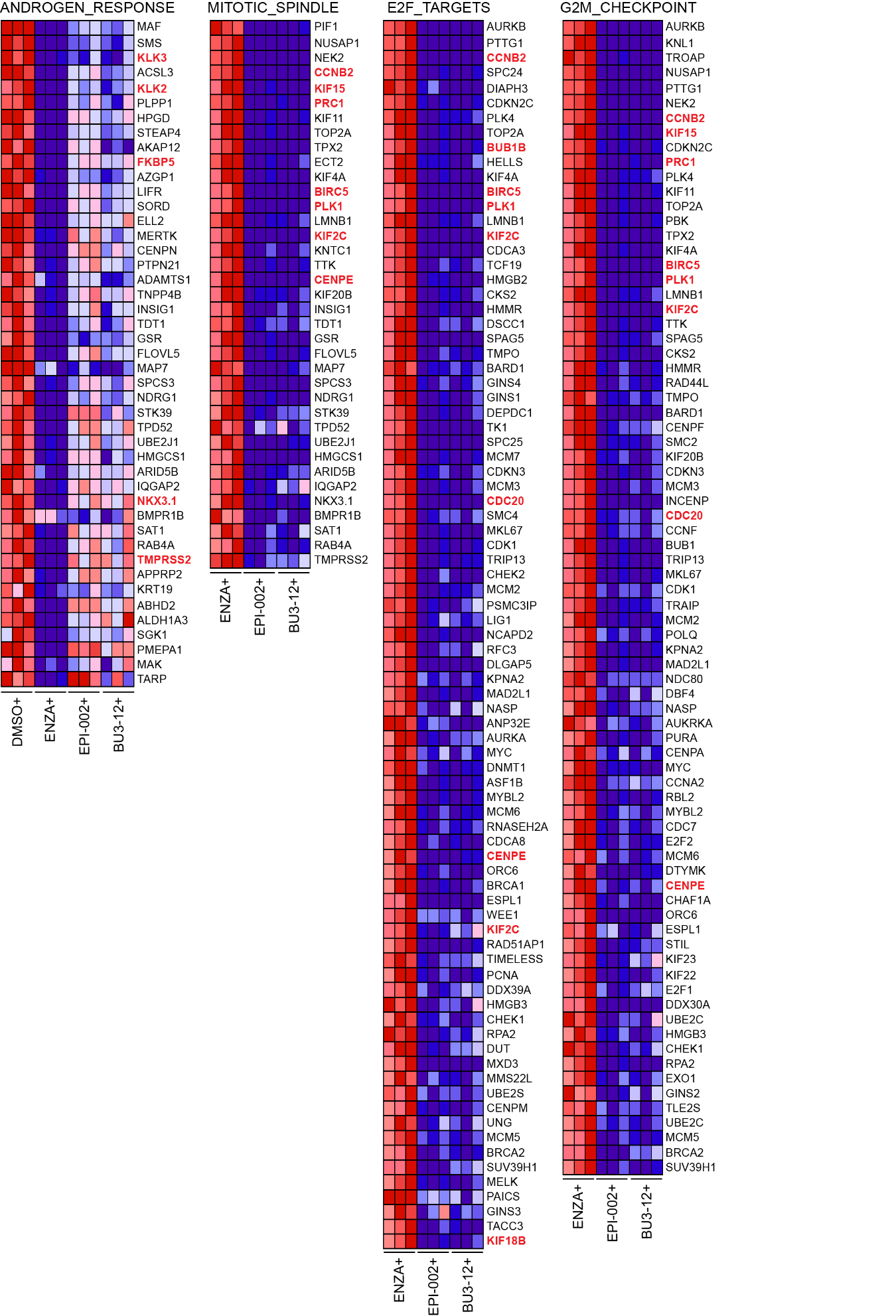


Figure. S5.

**Unique gene expression profiles associated with ARTADIs compared to enzalutamide in androgen-stimulated LNCaP cells.** Heatmap showing relative expression of core enriched genes contributing to the leading edge in GSEA enrichment plots shown in Fig. 5f. Genes highlighted in red were chosen for further qRT-PCR validation.


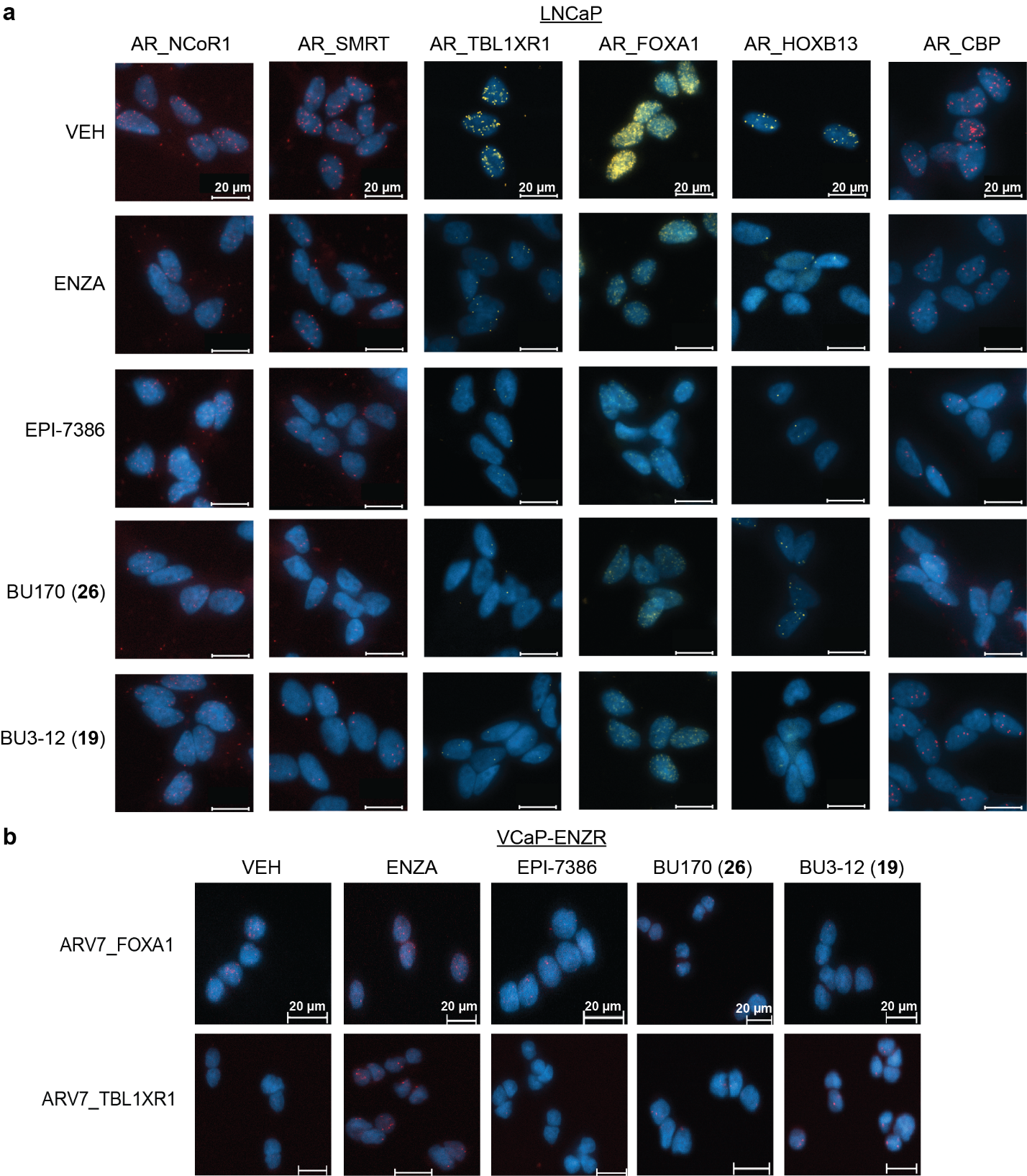


Figure. S6.

**Protein-protein interactions inhibited by inhibitors. a** Representative PLA images of AR interaction with NCoR1, SMRT, TBL1XR1, FOXA1, HOXB13 or CBP in androgen-stimulated LNCaP cells treated with vehicle, ENZA (5µM), EPI-7386 (5µM), BU170 (10µM), or BU3-12 (5µM). **b** Representative PLA images of AR-V7 interaction with FOXA1 and TBL1XR1 in VCaP-ENZR cells. Scale bars=20µm.


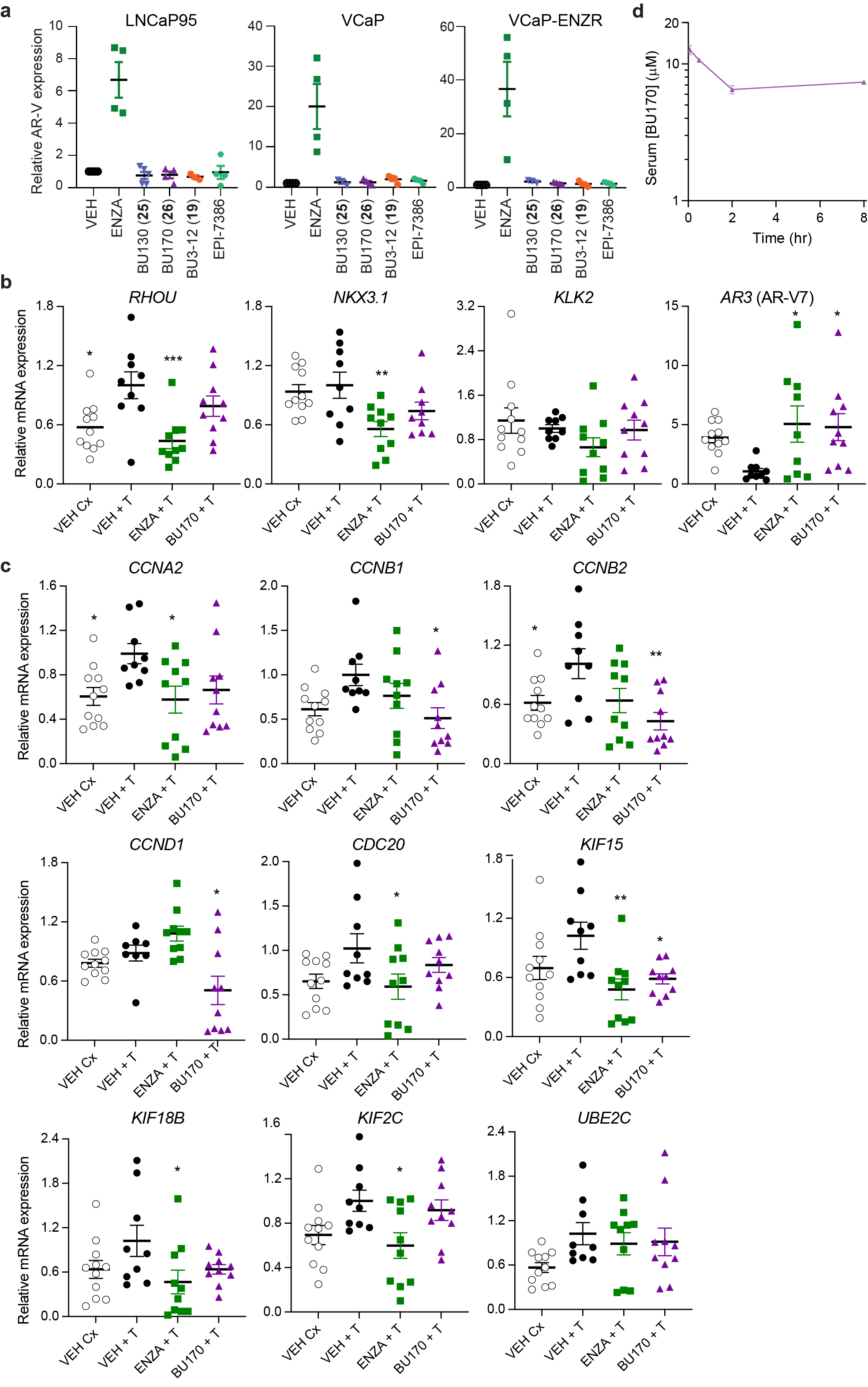


Figure. S7.

**ARTADIs as a treatment in the presence of androgen. a** Quantification of levels of AR-V protein normalized to β-actin from androgen-stimulated LNCaP-95, VCaP, and VCaP-ENZR cells treated with vehicle (VEH), enzalutamide (ENZA), BU130, BU170, BU3-12 and EPI-7386. Data are presented as the mean ± SEM from n=3-5 independent experiments. See Fig. 8a. **b** Transcript levels of androgen-regulated genes and *AR3*/AR-V7 normalized to housekeeping gene *SDHA* isolated from total RNA in harvested xenografts shown in Fig. 8b. **c** Transcript levels of cell cycle genes normalized to housekeeping gene *SDHA* isolated from total RNA in harvested xenografts shown in Fig. 8b. Data is normalized to VEH+T control and presented as mean ± SEM, and analyzed by one-way ANOVA with Dunnet’s correction. *p<0.05; ** p<0.01; *** p<0.001; ****p<0.0001. **d** Serum levels of BU170 after a single i.v. dose of 20 mg/kg body weight. n=2-3 animals per time point.


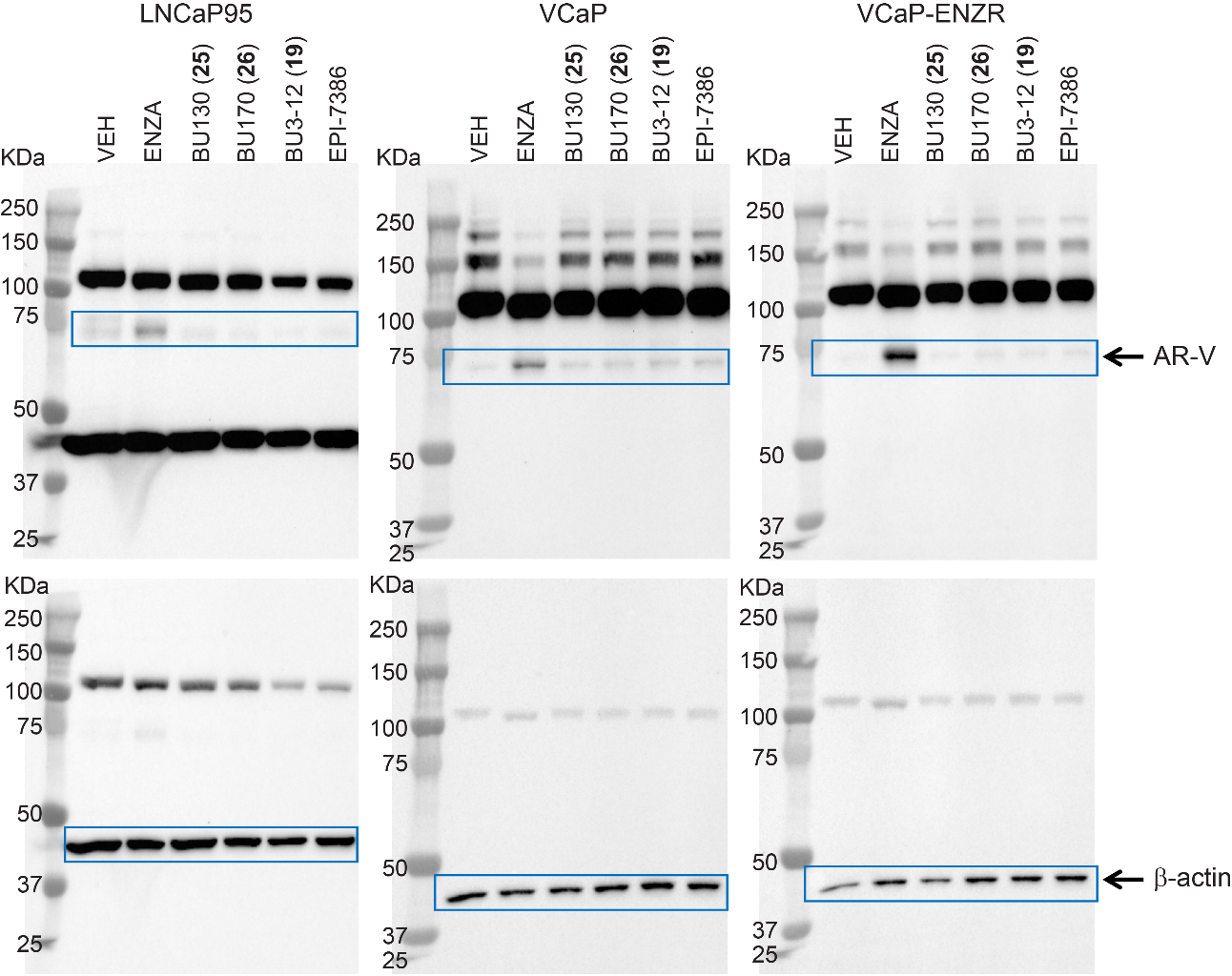


Figure. S8.

**ARTADIs as a treatment in the presence of androgen.** Representative images of original Western blot membranes are shown. See Fig 8a

Table S1.

Sequences of primers used in gene expression

| **Target** | **Direction** | **Sequence** |
| --- | --- | --- |
| ***KLK2*** | FWD  REV | 5’-TGTGTGCTAGAGCTTACTCTGA-3’  5’-CCACTTCCGGTAATGCACCA-3’ |
| ***KLK3*** | FWD  REV | 5'-TCATCCTGTCTCGGATTGTG-3'  5'-ATATCGTAGAGGGGGTGTGG-3' |
| ***FKBP5*** | FWD  REV | 5'-CGCAGGATATACGCCAACAT-3'  5'-GAAGTCTTCTTGCCCATTGC-3' |
| ***RHOU*** | FWD  REV | 5’- CCCGTGAGACTCCAACTCTG-3’  5’-TGAAGCAGAGCAGGAAGATG-3’ |
| ***NKX3.1*** | FWD  REV | 5’-CCGAGACGCTGGCAGAGACC-3’  5’-GCTTAGGGGTTTGGGGAAG-3’ |
| ***TMPRSS2*** | FWD  REV | 5’-GGACAGTGTGCACCTCAAAGAC-3’  5’-TCCCACGAGGAAGGTCCC-3’ |
| ***AKR1C3*** | FWD  REV | 5’-AGGTTTTTGAGTTCCAGTTG-3’  5’-GGCTAGCAAAACTATCACTG-3’ |
| ***DAB1*** | FWD  REV | 5’-GAACCTGTTATTCTGGACTTG-3’  5’-TACTGGTACACAGGATCTTC-3’ |
| ***DEPTOR*** | FWD  REV | 5’-AGGTGTCAAACTCTTCTAACC-3’  5’-CTTTTCATATAGCCCTCTGTCC-3’ |
| ***LRRN1*** | FWD  REV | 5’-CTCTCCTCGTCTTTCTC-3’  5’-TGAAGTCTGGAGATGGC-3’ |
| ***NOV*** | FWD  REV | 5’-ACCGTCAATGTGAGATGCTG-3’  5’-TCTTGAACTGCAGGTGGATG-3’ |
| ***SYTL2*** | FWD  REV | 5’-CCCATATGTAAAGGCCTAATTG-3’  5’-CAAGGTTTTCTTCACTACGAG-3’ |
| ***CCNA2*** | FWD  REV | 5’-CTCTACACAGTCACGGGACAAAG-3’  5’-CTGTGGTGCTTTGAGGTAGGT-3’ |
| ***CDC20*** | FWD  REV | 5’-CGGAAGACCTGCCGTTACATTC-3’  5’-CAGAGCTTGCACTCCACAGGTA-3’ |
| ***UBE2C*** | FWD  REV | 5’-AAAAGCTGTGGGGTTTTTCC-3’  5’-TGGTCTGCCCTGTATGATGT-3’ |
| ***B4GALT1*** | FWD  REV | 5’-AGAACCCAAATGTGAAGATG-3’  5’-CCTGGTTGATAACATAGATGC-3’ |
| ***NUP210*** | FWD  REV | 5’-GGGCTTGTTCCAGTGAT-3’  5’-CCACTATTTCGCTGCCTCAT-3’ |
| ***SLC3A2*** | FWD  REV | 5’-ACCCCTGTTTTCAGCTACGG-3’  5’-GGTCTTCACTCTGGCCCTTC-3’ |
| ***SLC30A7*** | FWD  REV | 5’-CAGTCATTCCCTCTTTAATGG-3’  5’-TCATGAGAATGAAGTGTCC-3’ |
| ***HIF1A*** | FWD  REV | 5’-AATGTTCCAATTCCAACTGC-3’  5’-AAAATCTCATCCAAGAAGCC-3’ |
| ***SNX14*** | FWD  REV | 5’-CTTCTCCTTTGGTTCCATTC-3’  5’-TCCACAGTCAAACAAAACTG-3’ |
| ***SDHA*** | FWD  REV | 5'-CAGCATGTGTTACCAAGCTGT-3'  5'-CGTGTCGTAGAAATGCCACCT-3' |

**Supplemental Information References**

1 Hirayama, Y., Tam, T., Jian, K., Andersen, R. J. & Sadar, M. D. Combination therapy with androgen receptor N-terminal domain antagonist EPI-7170 and enzalutamide yields synergistic activity in AR-V7-positive prostate cancer. *Mol Oncol* **14**, 2455-2470 (2020).

2 Leung, J. K., Tam, T., Wang, J. & Sadar, M. D. Isolation and characterization of castration-resistant prostate cancer LNCaP95 clones. *Hum Cell* **34**, 211-218 (2021).

3 Cleutjens, K. B. *et al.* An androgen response element in a far upstream enhancer region is essential for high, androgen-regulated activity of the prostate-specific antigen promoter. *Mol Endocrinol* **11**, 148-161 (1997).

4 Snoek, R. *et al.* Differential transactivation by the androgen receptor in prostate cancer cells. *Prostate* **36**, 256-263 (1998).

5 Xu, D. *et al.* Androgen Receptor Splice Variants Dimerize to Transactivate Target Genes. *Cancer Res* **75**, 3663-3671 (2015).

6 Yamaguchi, H. & Miyazaki, M. Refolding techniques for recovering biologically active recombinant proteins from inclusion bodies. *Biomolecules* **4**, 235-251 (2014).

7 Jerabek-Willemsen, M. *et al.* MicroScale Thermophoresis: Interaction analysis and beyond. *Journal of Molecular Structure* **1077**, 101-113 (2014).

8 Sadar MD, Mawji NR, et al. (2014). Diglycidic ether derivative therapeutics and methods for their use. (US Patent No. 8,686,050). U.S. Patent and Trademark Office.

9 Andersen RJ, Sadar MD, et al. (2015). Ester derivatives of androgen receptor modulators and methods for their use. (US Patent No. 9,173,939). U.S. Patent and Trademark Office.

10 Andersen RJ, and Sadar MD. (2016). Aziridine bisphenol ethers and related compounds and methods for their use. (US Patent No. 9,365,510). U.S. Patent and Trademark Office.

11 Andersen RJ, Banuelos CA, et al. (2016). Halogenated compounds for cancer imaging and treatment and methods for their use. (US Patent No. 9,375,496). U.S. Patent and Trademark Office.

12 Sadar MD, Mawji NR, et al. (2016). Bisphenol derivatives and their use as androgen receptor activity modulators. (US Patent No. 9,388,112). U.S. Patent and Trademark Office.

13 Sadar MD, Mawji NR, et al. (2016). Small molecule inhibitors of N-terminus activation of the androgen receptor. (US Patent No. 9,487,479). U.S. Patent and Trademark Office.

14 Yan L, Andersen RJ, et al. (2017). Androgen receptor modulators and methods for their use. (US Patent No. 9,682,933). U.S. Patent and Trademark Office.

15 Sadar MD, Mawji NR, et al. (2018). Diglycidic ether derivative therapeutics and methods for their use. (US Patent No. 9,862,667). U.S. Patent and Trademark Office.

16 Yan L, Andersen RJ, et al. (2018). Androgen receptor modulators and methods for their use. (US Patent No. 10,071,962). U.S. Patent and Trademark Office.

17 Andersen RJ, Kunzhong J, et al. (2019). Bisphenol ether derivatives and methods for using the same. (US Patent No. 10,471,023). U.S. Patent and Trademark Office.

18 Andersen RJ, Fernandez JG, et al. (2020). Heterocyclic compounds for cancer imaging and treatment and methods for their use. (US Patent No. 10,654,811). U.S. Patent and Trademark Office.

19 Zhou JH, Virsik P, et al. (2021). Androgen receptor modulators and methods for their use. (US Patent No. 11,059,795). U.S. Patent and Trademark Office.

20 Andersen RJ, and Sadar MD. (2021). Bisphenol derivatives and their use as androgen receptor activity modulators. (US Patent No. 11,142,508). U.S. Patent and Trademark Office.

21 Andersen RJ, Fernandez JG, et al. (2022). Heterocyclic compounds for cancer imaging and treatment and methods for their use. (US Patent No. 11,345,670). U.S. Patent and Trademark Office.

22 Zhou JH, Virsik P, et al. (2022). Androgen receptor modulators and methods for their use. (US Patent No. 11,485,713). U.S. Patent and Trademark Office.

23 Andersen RJ, Kunzhong J, et al. (2023). Bisphenol ether derivatives and methods for using the same. (US Patent No. 11,779,550). U.S. Patent and Trademark Office.

24 Andersen RJ, and Sadar MD. (2024). Bisphenol derivatives and their use as androgen receptor activity modulators. (US Patent No. 11,919,874). U.S. Patent and Trademark Office.

25 Virsik P, Zhou HJ, et al. (2024). Pharmacuetical compositions and combinations comprising inhibitors of the androgen receptor and uses thereof. (US Patent No. 12,109,179). U.S, Patent and Trademark Office.
