# Supplementary material for: Drugging the intrinsically disordered transactivation domain of androgen receptor
Source: Signal Transduct Target Ther. 2026 Apr 28;11:157. doi: 10.1038/s41392-026-02642-3 (PMC13121470; doi:10.1038/s41392-026-02642-3)

**Supplemental information: Synthesis and Compound Characterization**

This Supplemental Document describes the complete experimental details for the synthesis of two compounds BU3-12 (**19**) and BU-170 (**26**). The reactions and synthetic approaches used to prepare the other compounds discussed in the manuscript are only slight modifications of the reactions and synthetic approaches used to make BU3-12 (**19**) and BU-170 (**26**) so only the NMR and MS characterization data is given for these other compounds.

**General Experimental Procedures.** UV spectra were recorded with a Waters 996 Photodiode Array Detector. The ^1^H and ^13^C NMR spectra were recorded on Bruker AV-600 or AV400 spectrometers. ^1^H chemical shifts are referenced to the residual DMSO-*d*_6_ or CDCl_3_ signal (δ 2.49 and 7.24 ppm, respectively) and ^13^C chemical shifts are referenced to the DMSO-*d*_6_ or CDCl_3_ solvent peak (δ 39.5 and 77.0 ppm, respectively). Low and high resolution ESI-QIT-MS were recorded on a Bruker-Hewlett Packard 1100 Esquire–LC system mass spectrometer. Merck Type 5554 silica gel plates and Whatman MKC18F plates were used for analytical thin layer chromatography. Reversed-phase HPLC analyses were performed with a Waters 1525 Binary HPLC Pump system attached to a Waters 2998 Photodiode Array Detector. All solvents used for HPLC were Fisher HPLC grade.

**EXAMPLE 1: Synthesis of BU3-12 (19)**

**Synthetic Scheme:**

**Experimental Details:**

**Preparation of 1.1**

To a 100 mL round-bottom flask containing Bisphenol A (5000 mg, 21.9 mmol) in anhydrous DCM (40 mL) at 0°C was added in subsequent order Et_3_N (2216 mg, 21.9 mmol), Trityl chloride (4884 mg, 17.52 mmol) in 10 mL DCM added dropwise, followed by a catalytic amount of 4-dimethylamino pyridine (DMAP). The reaction was allowed to proceed overnight, slowly warming to room temperature. The reaction was diluted with 300 mL of EtOAc and washed with H_2_O (150 mL) and brine (150 mL) and the organic layer was dried over Na_2_SO_4_ and concentrated in vacuo. The crude extract was purified using silica gel flash chromatography eluting with hexanes/EtOAc (9:1) to afford **1.1** (8560 mg, 18.19 mmol) as a white solid in 83 % yield.

**Preparation of 1.2**

To a 250 mL round-bottom flask cooled to 0 °C containing **1.1** (1000 mg, 2.12 mmol) in anhydrous DMF (24 mL) was added 60% in mineral oil NaH (102 mg, 2.54 mmol). After stirring for 10 minutes (2R)-(-)-glycidyl tosylate (532 mg, 2.33 mmol) was added in 6 mL of DMF dropwise. The reaction was allowed to proceed overnight slowly warming to room temperature. The reaction was quenched with 1 mL of saturated NH_4_Cl and diluted with 150 mL of EtOAc and washed with H_2_O (3x 30 mL) and brine (3x 30 mL) and dried over Na_2_SO_4_ and concentrated in vacuo. The reaction was purified using silica gel flash chromatography eluting with hexanes/EtOAc (8:2) to afford **1.2** (928 mg, 1.76 mmol) in 83 % yield.

**Preparation of 1.3**

To a 100 mL round-bottom flask containing **1.2** (7320 mg, 13.90 mmol) dissolved in anhydrous DMF (50 mL) was added methane-sulfonamide (2642g, 27.78mmol) followed by Cs_2_CO_3_ (5430 mg, 16.67 mmol). The reaction mixture was equipped with a condenser and heated to 80°C overnight. After cooling, the reaction mixture was diluted with 300 mL of EtOAc and washed with H_2_O (3x 100 mL) and brine (3x 100 mL) and dried over Na_2_SO_4_ and concentrated in vacuo. The extract was then dissolved in 40 mL of DCM and ~12 mL of 6 M HCl was added, followed by the addition of methanol until the solution became homogenous. The mixture was stirred for 4 hours at room temperature before being concentrated in vacuo. The crude mixture was taken up in 200 mL of EtOAc and quenched with NaHCO_3_ until pH 6, the organic layer was then washed with H_2_O (1x 50 mL) and brine (1x 50 mL) and dried over Na_2_SO_4_ and concentrated in vacuo. The crude extract was then purified by silica gel flash chromatography eluting with DCM/MeOH (95:5) to afford **1.3** (3700 mg, 9.75 mmol) as a colorless oil in 70 % yield.

**Preparation of 1.4**

To a 100 mL round-bottom flask cooled to -20°C containing **1.3** (1560 mg, 4.11 mmol) dissolved in acetonitrile (36 mL) was added 10% NaOCl (6733 mg, 9.04 mmol). The reaction was stirred for 10 minutes before being quenched with saturated NaHSO_3_ (30 mL) and diluted with 150mL of EtOAc and washed with H_2_O (1x 20 mL) and brine (1x 20 mL) and dried over Na_2_SO_4_ then concentrated in vacuo. The crude extract was purified silica gel flash chromatography eluting with hexanes/EtOAc (2:3) to afford **1.4** (1370 mg, 3.06 mmol) as a pale-yellow oil in a yield of 74.45%.

**Preparation of 1.5**

To a 250 mL round-bottom flask cooled to 0 °C containing **1.4** (1770 mg, 3.95 mmol) in anhydrous DMF (36 mL) was added 60% in mineral oil NaH (190 mg, 4.74 mmol). After stirring for 10 minutes (2R)-(-)-glycidyl tosylate (902 mg, 3.95 mmol) was added in 6 mL of DMF dropwise. The reaction was allowed to proceed overnight slowly warming to room temperature. The reaction was quenched with 1 mL of saturated NH4Cl and diluted with 120 mL of EtOAc and washed with H2O (3x 30 mL) and brine (3x 30 mL) and dried over Na_2_SO_4_ and concentrated in vacuo. The reaction was purified using silica gel flash chromatography eluting with hexanes/EtOAc (3:1) to afford **1.5** (1715 mg, 3.40 mmol) in 86 % yield.

**Preparation of BU3-12 (19)**

To a 100 mL round-bottom flask containing **1.5** (2180 mg, 4.33 mmol) dissolved in anhydrous MeCN (36 mL) was added CeCl_3_·7H_2_O (339 mg, 0.909 mmol) and the reaction mixture was refluxed overnight. The coupled reaction mixture was filtered through celite using vacuum and the filter cake was washed with acetonitrile (3 x 60mL) and then concentrated in vacuo. The crude mixture was purified by silica gel flash chromatography eluting with hexanes/EtOAc (2:3) to afford BU3-12 **(19)** (2120 mg, 3.92 mmol) as a white solid in 91 % yield.

**EXAMPLE 2: Synthesis of BU170 (26)**

**Synthetic Scheme:**

**Experimental Details:**

**Preparation of 2.1**

To a 100 mL round-bottom flask cooled to 0°C containing 2-methyl glycidol (1.00 g, 11.35 mmol) in anhydrous DCM (30 mL) was added Et_3_N (3.19 mL, 22.70 mmol) followed by tosyl chloride (2.27 g, 11.92 mmol) in 10 mL of DCM added dropwise then a catalytic amount of DMAP was added. The reaction mixture was stirred overnight and allowed to warm to room temperature slowly. The reaction mixture then was diluted with 120 mL of DCM and washed with H_2_O (2x 20 mL) and brine (2x 20 mL) and dried over Na_2_SO_4_ and concentrated in vacuo. The crude extract was purified with silica gel flash chromatography eluting with hexanes/EtOAc (4:1) to afford **2.1** (2.58g, 10.65mmol) as a colorless oil in 94 % yield. ¹H NMR (400 MHz, DMSO-*d_6_*) δ: 7.81 (d, J = 8.3Hz, 2H), 7.51 (d, J = 8.0Hz, 2H), 4.25 (d, J = 10.9Hz, 1H), 3.87 (d, J = 10.9Hz, 1H), 2.70 (d, J = 4.8Hz, 1H), 2.65 (d, J = 4.8Hz, 1H), 2.44 (s, 3H), 1.23 (s, 3H).

**Preparation of 2.2**

To a 100 mL round-bottom flask containing phenol (2.20 g, 23.4 mmol) dissolved in DCM (40 mL) was added 1,3 difluoroacetone (1.00 g, 10.6 mmol). The flask was subsequently cooled to -10°C and Iron (III) chloride (1.72 g, 10.6 mmol) was added. The reaction was stirred overnight before the flask was allowed to slowly warm to room temperature. DCM was removed in vacuo and the crude oil was taken up in 150 mL of ethyl acetate/acetone (2:1) and washed with H_2_O (25 mL) and brine (25 mL). The organic layer was dried over Na_2_SO_4_ and concentrated in vacuo. The crude extract was purified using silca gel flash chromatography eluting with hexanes/ethyl acetate (4:1) to afford **2.2** (406.1mg, 1.54mmol) as a yellow oil in 15 % yield. ¹H NMR (400 MHz, DMSO-*d_6_*) δ 9.40 (s, 2H), 7.01 (d, J = 8.7 Hz, 4H), 6.71 (d, J = 8.7 Hz, 4H), 5.01 (s, 2H), 4.89 (s, 2H) ppm; ^13^C NMR (100 MHz, DMSO-*d_6_*) δ 156.1, 131.2, 128.7, 115.0, 85.7, 83.9, 50.4 ppm; ^19^F NMR (300 MHz, DMSO- *d_6_*) δ -219.8 ppm.

**Preparation of 2.3**

To a 100 mL round-bottom flask containing **2.2** (460 mg, 1.74 mmol) in anhydrous DCM (20 mL) at 0 °C was added in subsequent order Et_3_N (0.25 mL, 1.74 mmol), Trityl chloride (325 mg, 1.17 mmol) in 4 mL DCM added dropwise, followed by a catalytic amount of 4-dimethylamino pyridine (DMAP). The reaction was allowed to proceed overnight, slowly warming to room temperature. The reaction was diluted with 80 mL of EtOAc and washed with H_2_O (15 mL) and brine (15 mL) and the organic layer was dried over Na_2_SO_4_ and concentrated *in vacuo*. The crude extract was purified using silica gel flash chromatography eluting with hexanes/EtOAc (9:1) to afford **2.3** (360 mg, 0.711 mmol) as a white solid in 61 % yield. ¹H NMR (400 MHz, DMSO-*d_6_*) δ 9.41 (s, 1H), 7.42 (d, J = 7.6 Hz, 3H), 7.30 (m, 12H), 7.03 (d, J = 8.6 Hz, 2H), 6.89 (dd, J = 8.8, 3.7 Hz, 2H), 6.73 (d, J = 8.7 Hz, 2H), 6.69 (d, J = 8.7 Hz, 1H), 6.63 (d, J = 8.8 Hz, 1H), 4.98 (d, J = 30.2 Hz, 2H) ppm; ^13^C NMR (100 MHz, DMSO-*d_6_*)) δ 156.1, 147.8, 143.7, 128.7, 128.6, 128.4, 127.9, 127.8, 127.5, 127.2, 126.6, 120.1, 115.0, 89.6, 80.5 ppm; ^19^F NMR (300 MHz, DMSO-*d_6_*) δ: -219.8, -220.4 ppm.

**Preparation of 2.4**

To a 250 mL round-bottom flask cooled to 0 °C containing **2.3** (3.17 g, 6.26 mmol) in anhydrous DMF (40 mL) was added 60% in mineral oil NaH (300 mg, 7.51 mmol). After stirring for 10 minutes **2.1** (1.60 g, 6.57 mmol) was added in 10 mL of DMF dropwise. The reaction was allowed to proceed overnight slowly warming to room temperature. The reaction was quenched with 30 mL of saturated NH_4_Cl and diluted with 120 mL of EtOAc and washed with H_2_O (3x 30 mL) and brine (3x 30 mL) and dried over Na_2_SO_4_ and concentrated in vacuo. The reaction was purified using silica gel flash chromatography eluting with hexanes/EtOAc (8:2) to afford **2.4** (2.41 g, 4.20 mmol) in 67 % yield. ¹H NMR (400 MHz, DMSO-*d_6_*) δ 7.42 (d, J = 7.4Hz, 6H), 7.35 (t, J = 7.2 Hz, 6H), 7.29 (t, J = 7.1 Hz, 4H), 7.00 (d, J = 8.8 Hz, 2H), 6.90 (d, J = 1.9, 2H), 6.88 (d, J = 1.8 Hz, 2H), 6.62 (d, J = 8.8 Hz, 2H), 4.98 (s, 2H), 4.86, (s, 2H), 4.12 (d, J = 10.8 Hz, 1H), 3.87 (d, J = 10.8 Hz, 1H), 2.81 (d, J = 5.0 Hz, 1H), 2.71 (d, J = 5.0 Hz, 1H), 2.05 (s, 1H), 1.39 (s, 3H) ppm; ^13^C NMR (100 MHz, DMSO-*d_6_*) δ 143.6, 128.7, 128.4, 127.9, 127.2, 120.2, 114.3, 71.3, 55.2, 50.8, 18.2 ppm; ^19^F NMR (300 MHz, DMSO-*d_6_*) δ -220.8 ppm.

**Preparation of 2.5**

To a 100 mL round-bottom flask containing **2.4** (2.41 g, 4.20 mmol) dissolved in anhydrous DMF (50 mL) was added methane-sulfonamide (6.18g, 63.0mmol) followed by Cs_2_CO_3_ (1.64 g, 5.04 mmol). The reaction mixture was equipped with a condenser and heated to 80 °C overnight. The cooled reaction mixture was diluted with 150 mL of EtOAc and washed with H_2_O (3x 30 mL) and brine (3x 30 mL) and dried over Na_2_SO_4_ and concentrated in vacuo. The extract was then dissolved in 40 mL of DCM and ~6 mL of 6 M HCl was added, followed by the addition of methanol until the solution became homogenous. The mixture was stirred for 4 hours at room temperature before being concentrated in vacuo. The crude mixture was taken up in 150 mL of EtOAc and quenched with NaHCO_3_ until pH 6, and then the organic layer was then washed with H_2_O (1x 30 mL) and brine (1x 30 mL) and dried over Na_2_SO_4_ and concentrated in vacuo. The crude extract was then purified by silica gel flash chromatography eluting with DCM/MeOH (95:5) to afford **2.5** (1.35 g, 3.25 mmol) as a colorless oil in 77.4% yield. ¹H NMR (400 MHz, DMSO-*d_6_*) δ 9.40 (s, 1H), 7.14 (d, J = 8.8 Hz, 2H), 7.02 (d, J = 8.7 Hz, 2H), 6.90 (d, J = 8.8 Hz, 2H), 6.72 (d, J = 8.7 Hz, 2H), 5.04 (s, 2H), 4.91 (s, 2H), 4.89 (s, 1H), 3.80 (d, J = 9.3 Hz, 1H), 3.73 (d, J = 9.3 Hz, 1H), 3.05 (m, 2H), 2.91 (s, 3H), 1.20 (s, 3H) ppm; ^13^C NMR (100 MHz, DMSO-*d_6_*) δ 158.1, 156.8, 129.4, 115.7, 115.0, 86.3, 73.1, 71.1, 51.2, 50.1, 23.1 ppm; ^19^F NMR (300 MHz, DMSO-*d_6_*) δ -220.1 ppm.

**Preparation of 2.6**

To a 100 mL round-bottom flask cooled to -20 °C containing **2.5** (1.35 g, 3.25 mmol) dissolved in acetonitrile (40 mL) was added 10% NaOCl (6.05 g, 8.13 mmol). The reaction was stirred for 10 minutes before being quenched with saturated NaHSO_3_ (30 mL) and diluted with 100 mL of EtOAc and washed with H_2_O (1x 20 mL) and brine (1x 20 mL) and dried over Na_2_SO_4_ then concentrated in vacuo. The crude extract was purified silica gel flash chromatography eluting with hexanes/EtOAc (2:3) to afford **2.6** (1.03 g, 2.13 mmol) as a pale-yellow oil in a yield of 66 %. ¹H NMR (400 MHz, DMSO-*d_6_*) δ 10.20 (s, 1H), 7.19 (s, 2H), 7.16 (d, J = 8.6 Hz, 2H), 6.93 (d, J = 8.6 Hz, 2H), 6.92 (t, J = 6.5 Hz, 1H), 5.10 (dd, J_1,3_ = 16.5 Hz, J_2,3_ = 6.7 Hz, 2H), 4.98 (dd, J_1,3_ = 16.4 Hz, J_2,3_ = 6.9 Hz, 2H), 4.90 (s, 1H), 3.82 (d, J = 9.3 Hz, 1H), 3.75 (d, J = 9.3 Hz, 1H), 3.06 (m, 2H), 2.91 (s, 3H), 1.20 (s, 3H) ppm; ^13^C NMR (100 MHz, DMSO-*d_6_*) δ 157.7, 148.0, 133.9, 128.8, 128.0, 122.1, 114.6, 72.5, 70.4, 50.8, 49.5, 22.6 ppm; ^19^F NMR (300 MHz, DMSO-*d_6_*) δ -221.1 ppm.

**Preparation of BU-170 (26)**

To a 50 mL round bottom flask cooled to 0°C containing **2.6** (339 mg, 0.699 mmol) in anhydrous DMF (12 mL) was added K_2_CO_3_ (102 mg, 0.734 mmol) followed by glycidyl tosylate (168 mg, 0.734 mmol) in 3 mL of DMF dropwise. The reaction mixture was stirred at 0°C for 15 minutes before the ice bath was removed and the reaction was heated to 40 °C overnight. The reaction mixture was quenched with water (10 mL) and diluted with 80 mL of EtOAc and washed with H_2_O (2x 20 mL) and brine (3x 20 mL) and dried over Na_2_SO_4_ and concentrated in vacuo. The crude mixture was used without purification and added to a dry flask and dissolved in 40 mL of acetonitrile followed by CeCl_3_·7H_2_O (339 mg, 0.909 mmol) and refluxed overnight. The reaction was filtered through celite by vacuum filtration and the filter cake was washed with acetonitrile (3 x 60 mL) and then concentrated in vacuo. The crude mixture was purified by silica gel flash chromatography eluting with hexanes/EtOAc (2:3) to afford **BU170 (26)** (384 mg, 0.650 mmol) as a white solid in 93 % yield.

**3. Spectral Characterization for the Final Products**:

**3.1 EPI-002 (1)**

¹H NMR (600 MHz, DMSO-*d_6_*) δ 7.09-7.06 (m, 4H), 6.82-6.79 (m, 4H), 5.50 (d, J = 6.0 Hz, 1H), 4.90 (d, J = 6.0 Hz, 1H), 4.62 (t, J = 6.0 Hz, 1H), 4.02-3.97 (m, 1H), 3.93-3.90 (m, 3H), 3.80-3.77 (m, 1H), 3.76-3.70 (m, 2H), 3.65-3.62 (m, 1H), 3.43-3.38 (m, 2H), 1.55(s, 6H) ppm; ^13^C NMR (150 MHz, DMSO-*d_6_*) δ: 156.51, 156.11, 142.91, 142.45, 127.46, 127.40, 113.88, 113.81, 69.97, 69.44, 68.84, 68.62, 62.74, 46.80, 41.16, 30.75 ppm; ESI-HRMS: *m/z* calculated for C_21_H_27_ClKO_5_ [M + K]^+^, 433.1184; found, 433.1179.

C_18_ reversed-phase HPLC trace of **EPI-002** (**1**) dissolved in DMSO using a InertSustain 5 µm, 25 x 1 cm column with 3:2 MeCN/H_2_O as eluent at a flow rate of 2 mL/min with UV detection at 202 and 228 nm.


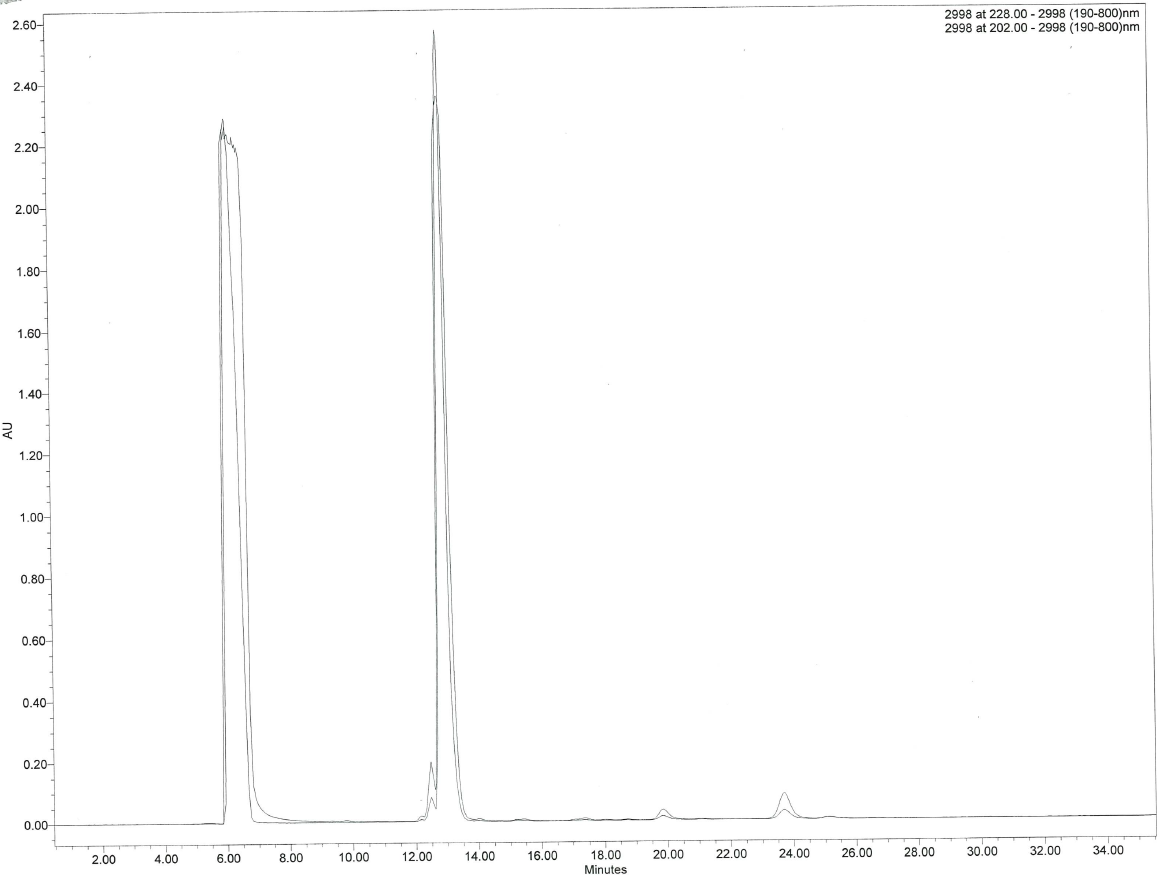


^1^H NMR Spectrum of **EPI-002** (**1**) recorded at 600 MHz in DMSO-*d*_6_


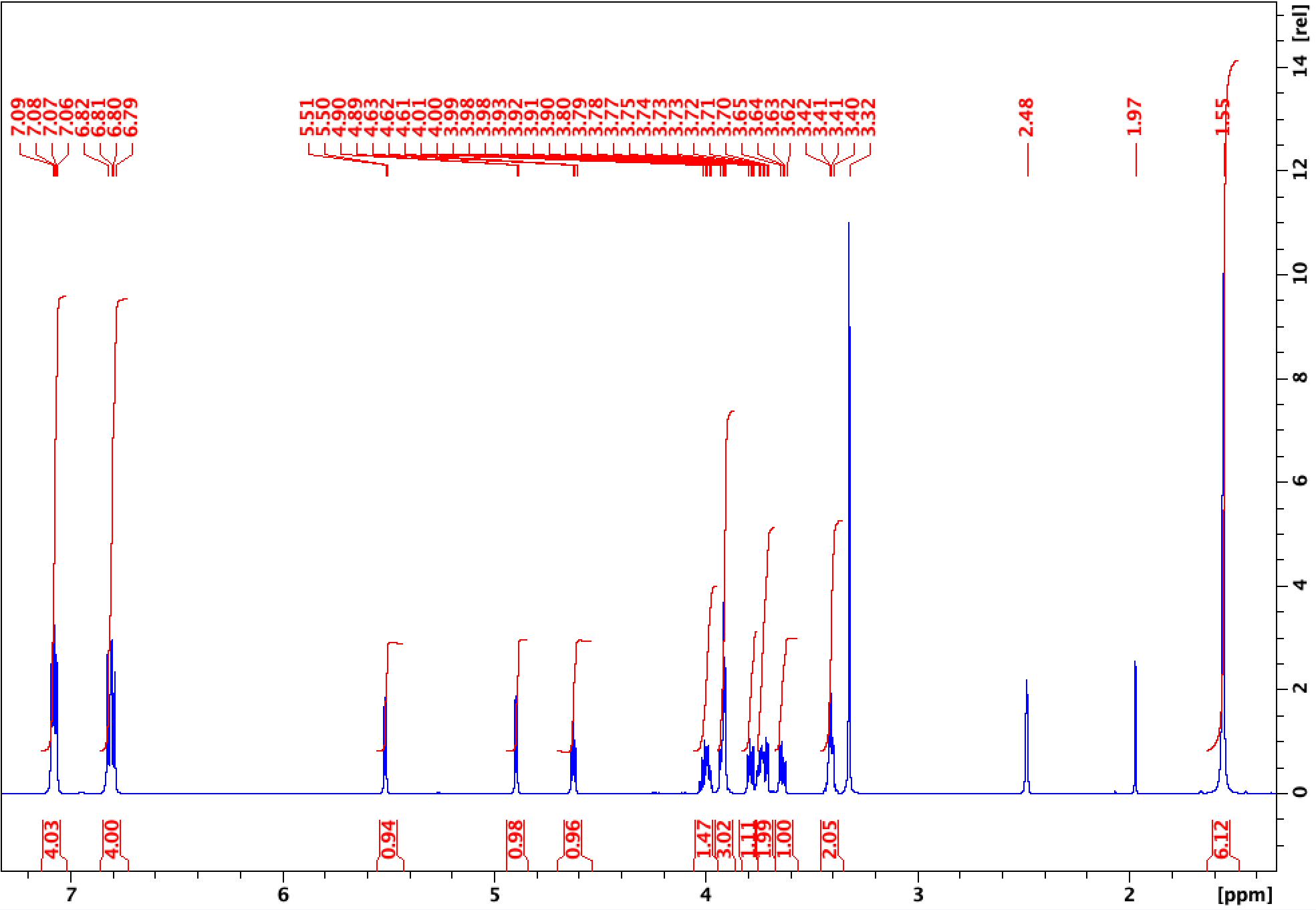


^13^C NMR Spectrum of **EPI-002** (**1**) recorded at 150 MHz in DMSO-*d*_6_


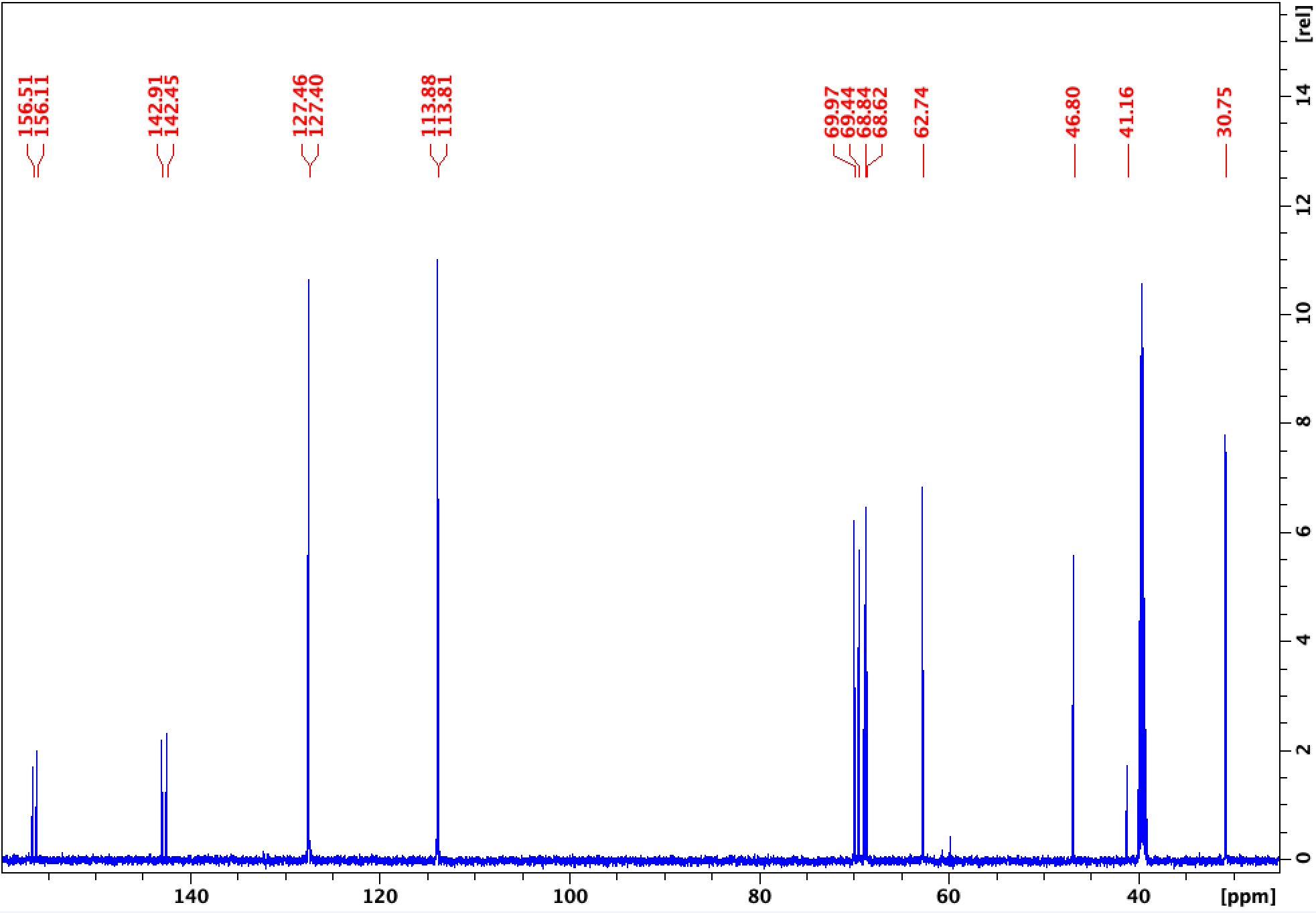


**3.2 EPI-506 (2)**

¹H NMR (600 MHz, DMSO-*d_6_*) δ 7.11-7.09 (m, 4H), 6.87-6.84 (m, 4H), 5.29-5.25 (m, 2H), 4.32 (dd, J_1_ = 12.0Hz, J_2_ = 6.0 Hz, 1H), 4.22 (dd, J_1_ = 12.0 Hz, J_2_ = 6.0 Hz, 1H), 4.14-4.09 (m, 4H), 3.93 (dd, J_1_ = 12.0 Hz, J_2_ = 6.0 Hz, 1H), 3.87 (dd, J_1_ = 12.0 Hz, J_2_ = 6.0 Hz, 1H), 2.06 (s, 3H), 2.03 (s, 3H), 2.02 (s, 3H), 1.57 (s, 6H) ppm; ^13^C NMR (150 MHz, DMSO-*d_6_*) δ 170.06, 169.75, 169.60, 155.72, 155.65, 143.11, 143.01, 127.39, 127.38, 113.9, 70.71, 69.35, 66.19, 65.92, 62.01, 43.04, 41.12, 30.57, 20.62, 20.55, 20.42 ppm; ESI-HRMS: *m/z* calculated for C_27_H_33_ClNaO_8_ [M + Na]^+^, 543.1762; found, 543.1766.

C_18_ reversed-phase HPLC trace of **EPI-506** (**2**) dissolved in DMSO using a InertSustain 5 µm, 25 x 1 cm column with 13:7 MeCN/H_2_O as eluent at a flow rate of 2 mL/min with UV detection at 197 nm.


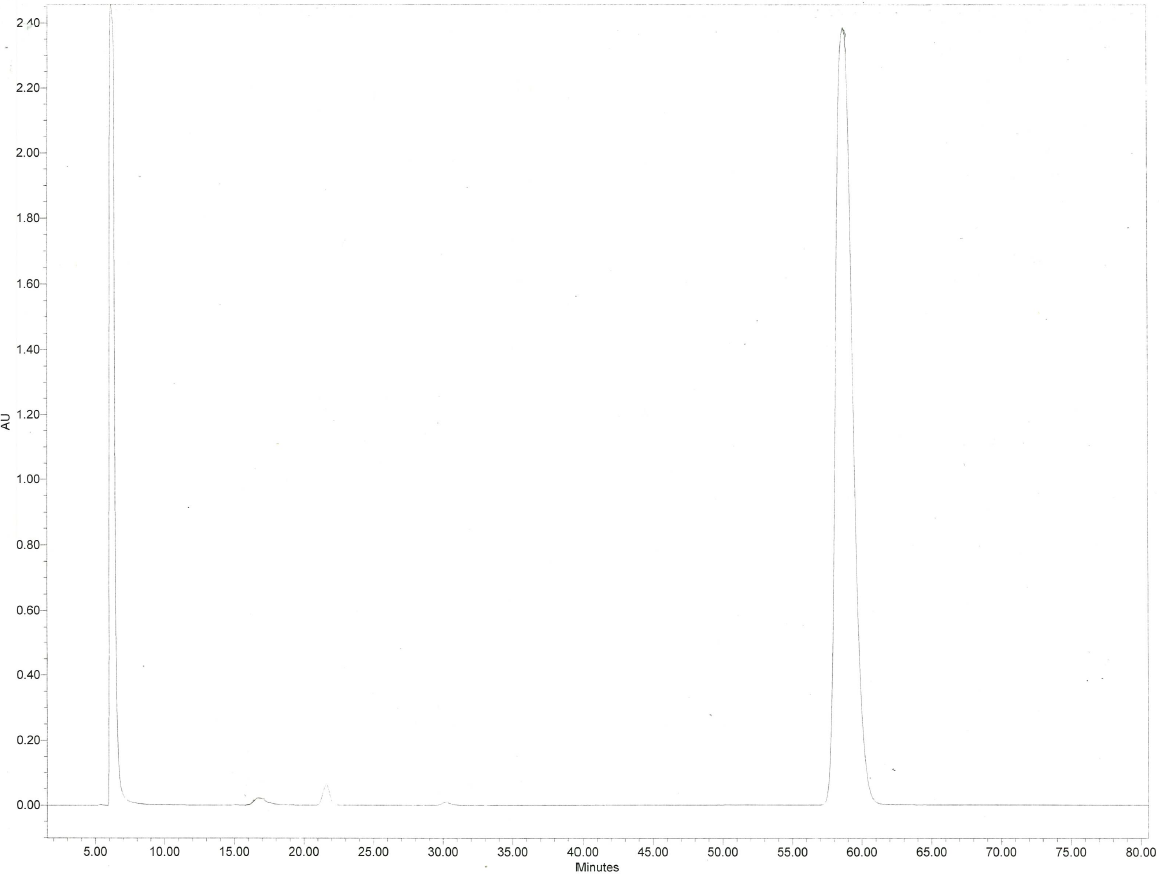


^1^H NMR Spectrum of **EPI-506** (**2**) recorded at 600 MHz in DMSO-*d*_6_

_
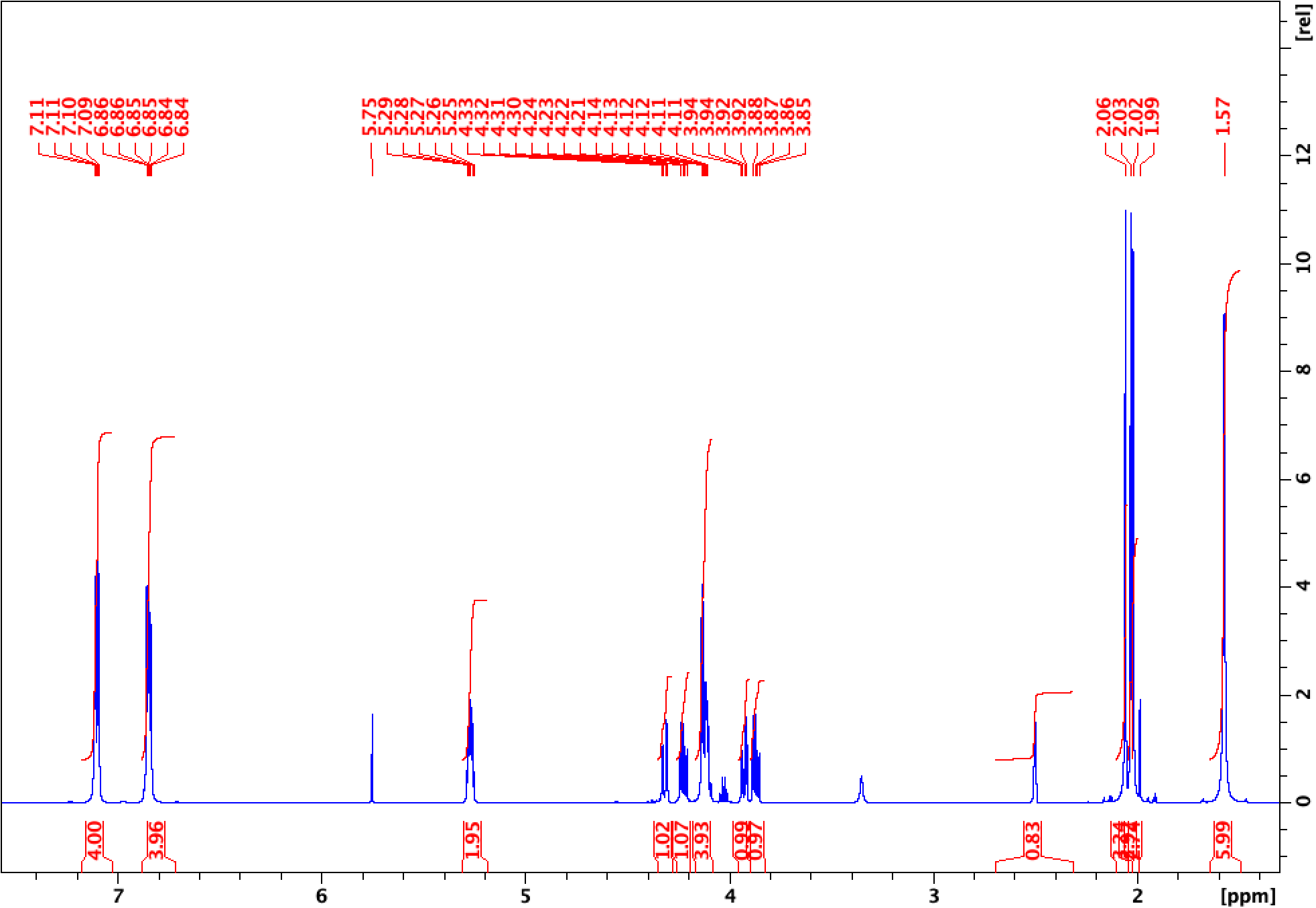
_

^13^C NMR Spectrum of **EPI-506** (**2**) recorded at 150 MHz in DMSO-*d*_6_

_
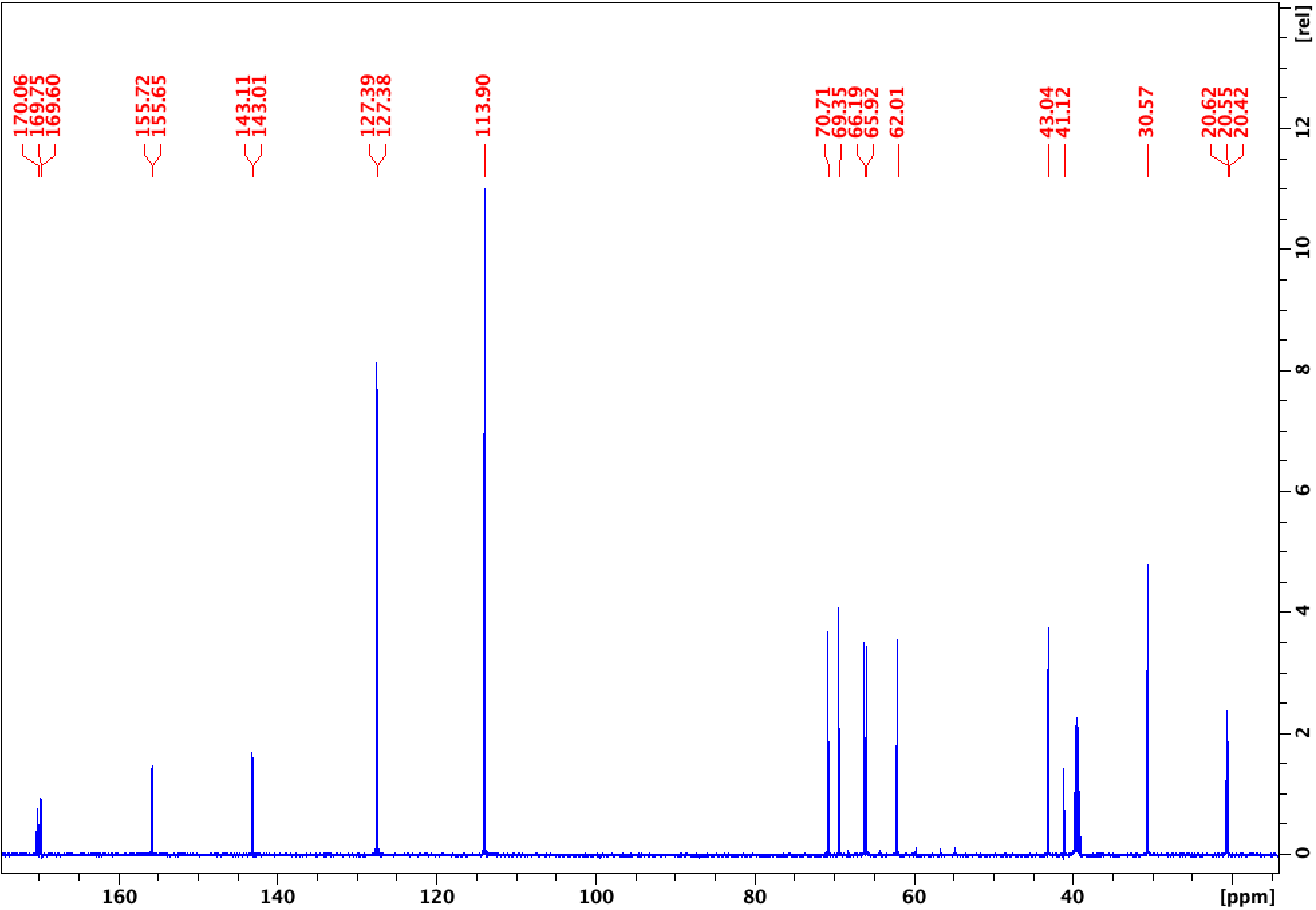
_

**3.3 EPI-087 (3)**

¹H NMR (400 MHz, DMSO-*d_6_*) δ 7.10-7.08 (m, 4H), 6.84-6.80 (m, 4H), 4.94 (d, J = 8.0 Hz, 1H), 4.68 (t, J = 8.0 Hz, 1H), 4.04 (t, J = 8.0 Hz, 2H), 3.96-3.92 (m, 1H), 3.82-3.79 (m, 1H), 3.79-3.75 (m, 3H), 2.17-2.11 (m, 2H), 1.57 (s, 6H) ppm; ^13^C NMR (150 MHz, CDCl_3_) δ 156.49, 156.20, 143.76, 143.23, 127.78, 127.70, 113.87, 113.82, 70.36, 69.10, 64.12, 63.68, 41.68, 41.55, 32.30, 30.98 ppm; ESI-HRMS: *m/z* calculated for C_21_H_27_ClKO_4_ [M + K]^+^, 417.1235; found, 417.1233.

^1^H NMR Spectrum of **EPI-087** (**3**) recorded at 400 MHz in DMSO-*d*_6_


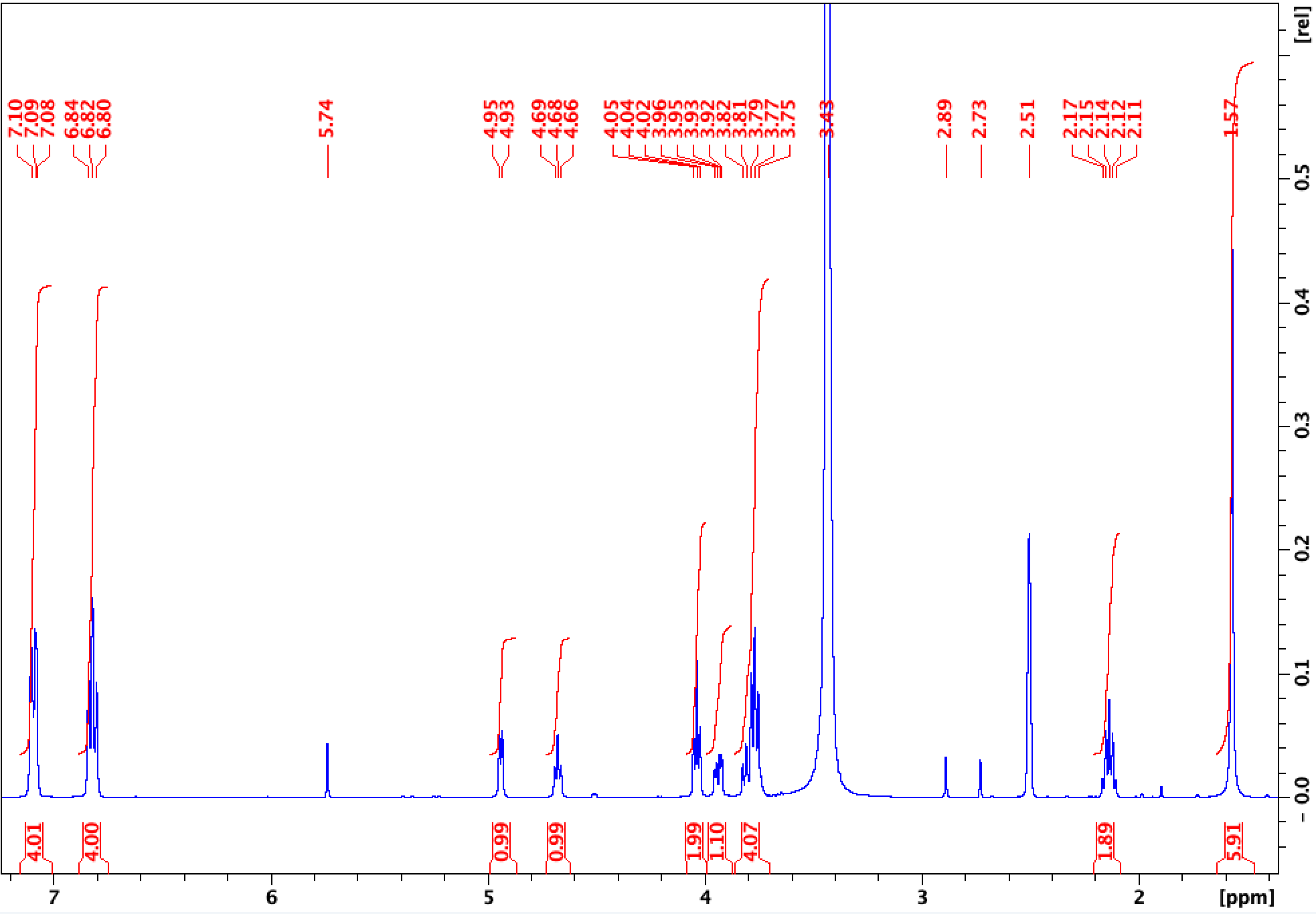


^13^C NMR Spectrum of **EPI-087** (**3**) recorded at 150 MHz in CDCl_3_

_
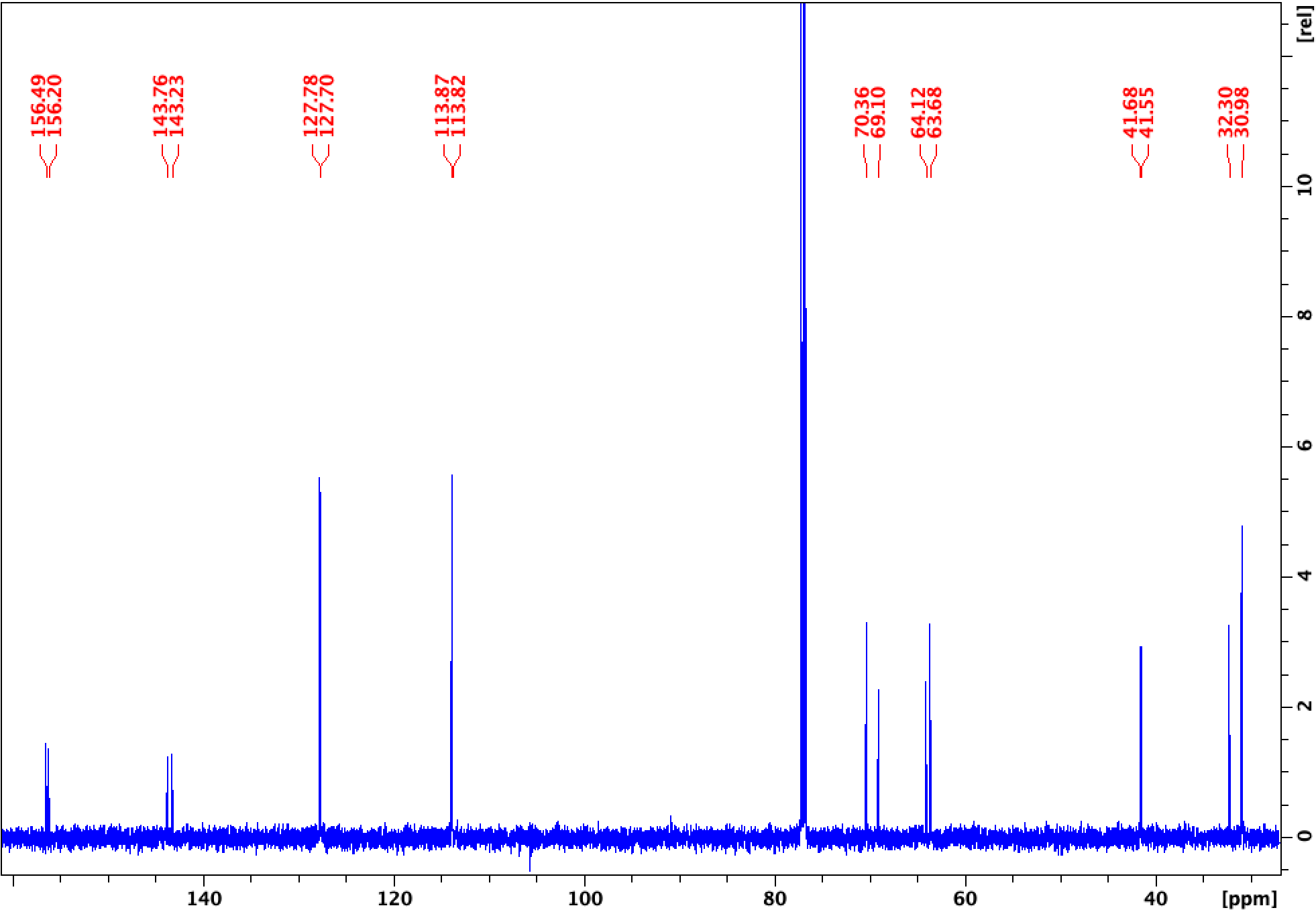
_

**3.4 EPI-20000 (4)**

^1^H NMR (400 MHz, CDCl_3_) δ 7.23 (d, J = 2.3 Hz, 1H), 7.13 (d, J = 8.8 Hz, 2H), 7.05 (dd, J = 8.6, 2.4, 1H), 6.86-6.82 (m, 3H), 4.26-4.21 (m, 1H), 4.17-4.08 (m, 3H), 4.04-4.02 (m, 2H), 3.86-3.73 (m, 4H), 1.63 (s, 6H) ppm; ^13^C NMR (100 MHz, CDCl_3_) δ 156.29, 151.40, 145.26, 142.75, 128.60, 127.64, 126.01, 122.48, 113.94, 113.40, 70.26, 69.65, 69.59, 69.03, 63.55, 53.30, 45.55, 41.72, 30.73 ppm; ESI-HRMS: *m/z* calculated for C_21_H_26_BrClKO_5_ [M + K]^+^, 511.0289; found, 511.0284.

C_18_ reversed-phase HPLC trace of **EPI-20000** (**4**) dissolved in DMSO using a InertSustain 5 µm, 25 x 1 cm column with 3:2 MeCN/H_2_O as eluent at a flow rate of 2 mL/min with UV detection at 197 and 228 nm.


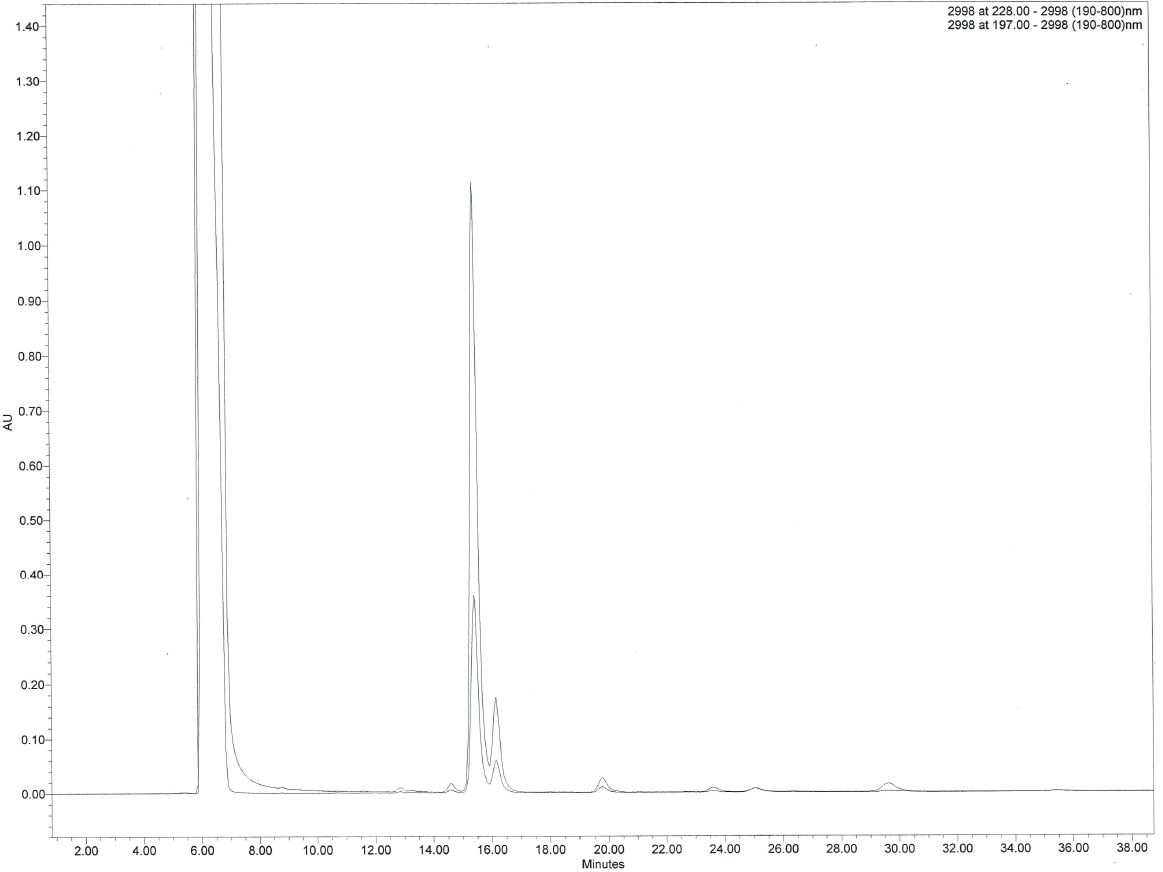


^1^H NMR Spectrum of **EPI-20000** (**4**) recorded at 400 MHz in CDCl_3_


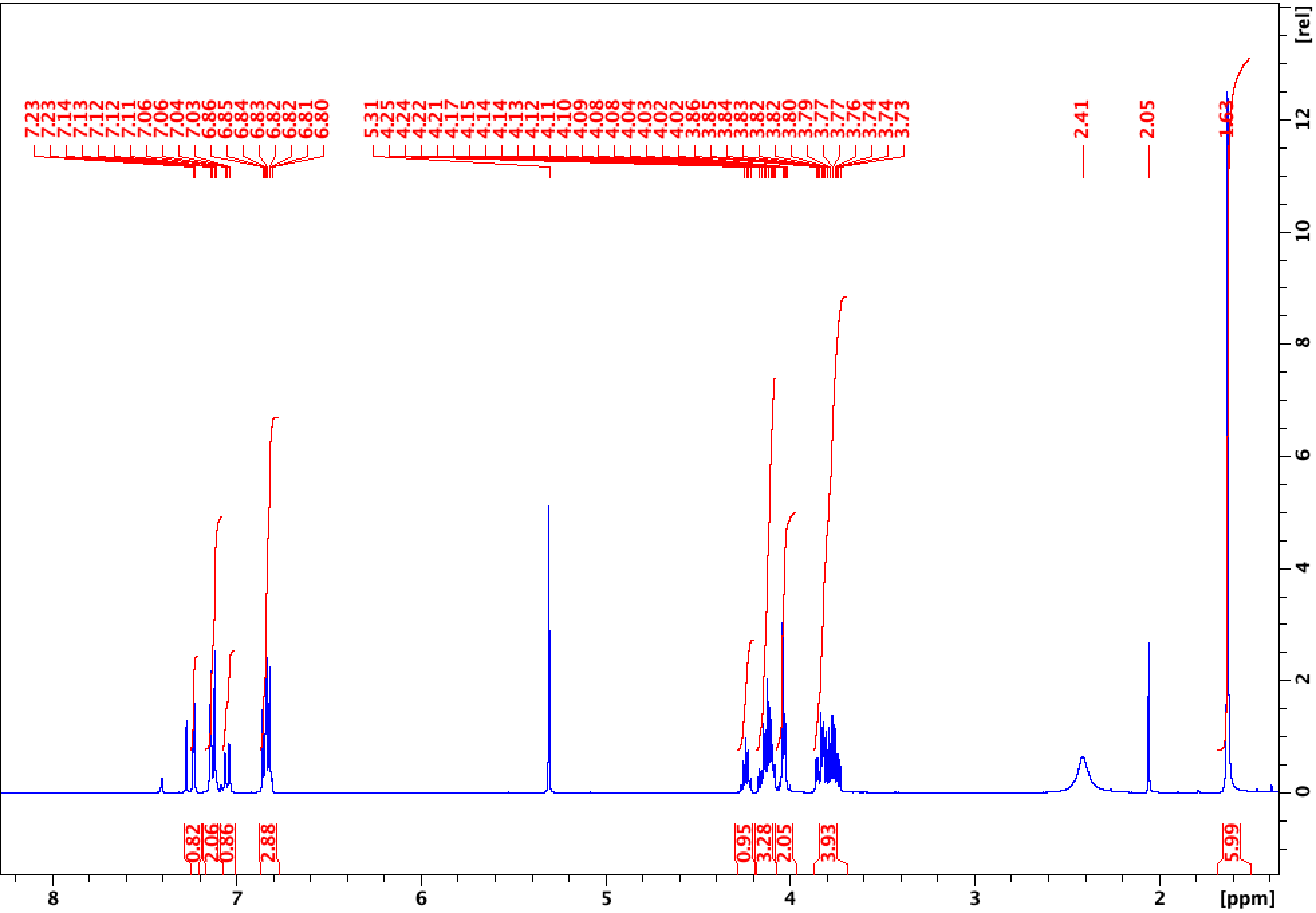


^13^C NMR Spectrum of **EPI-20000** (**4**) recorded at 100 MHz in CDCl_3_


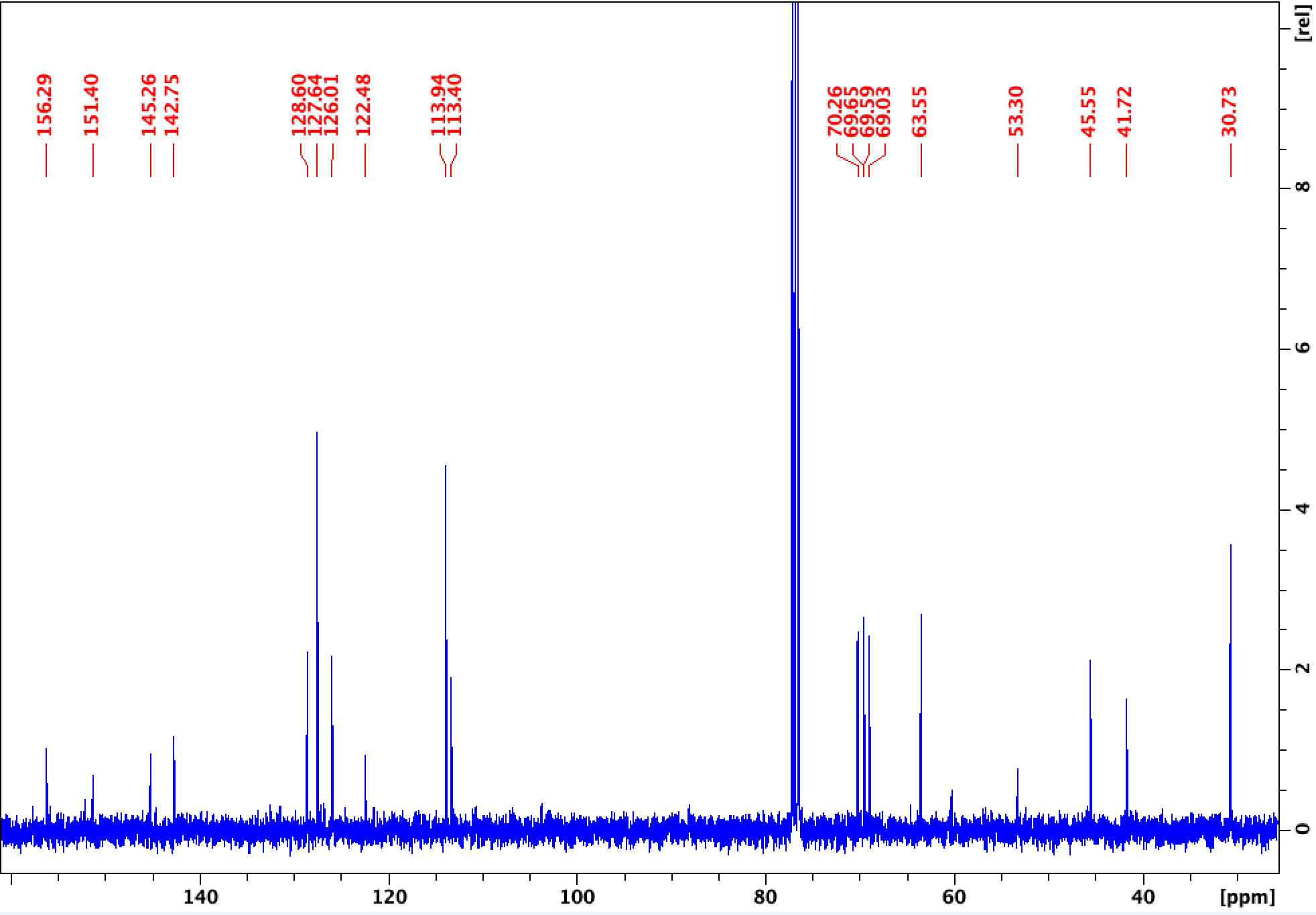


**3.5 EPI-12000 (5)**

^1^H NMR (600 MHz, CDCl_3_) δ 7.28-7.23 (m, 1H), 7.13-7.11 (m, 2H), 7.04 (d, J = 6.0 Hz, 1H), 6.85-6.82 (m, 3H), 4.25-4.24 (m, 1H), 4.16-4.10 (m, 3H), 4.02 (m, 2H), 3.83-3.82 (m, 2H), 3.78-3.76 (m, 2H), 1.63 (s, 6H) ppm; ^13^C NMR (150 MHz, CDCl_3_) δ 155.99, 151.09, 144.90, 142.39, 128.28, 127.33, 125.72, 122.08, 113.62, 113.03, 70.03, 69.31, 68.65, 63.26, 45.29, 41.4, 30.43 ppm; ESI-LRMS: *m/z* calculated for C_21_H_26_Cl_2_NaO_5_ [M + Na]^+^, 451.1055; found, 451.3.

C_18_ reversed-phase HPLC trace of **EPI-12000** (**5**) dissolved in DMSO using a InertSustain 5 µm, 25 x 1 cm column with 3:2 MeCN/H_2_O as eluent at a flow rate of 2 mL/min with UV detection at 197 and 228 nm.


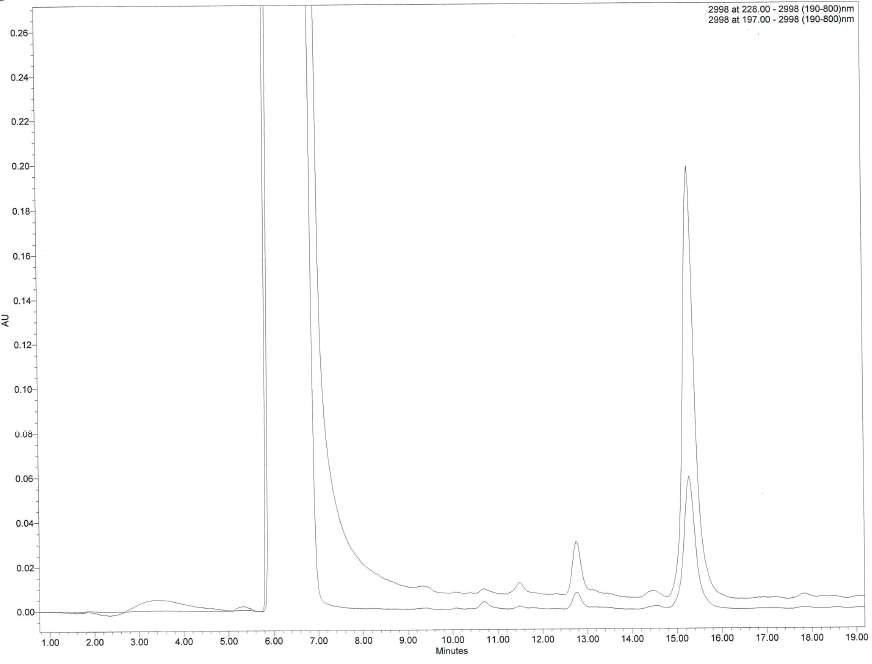


**3.6 EPI-10000 (6)**

¹H NMR (400 MHz, CDCl_3_) δ 7.22 (d, J = 4.0 Hz, 1H), 7.13 (d, J = 8.0 Hz, 2H), 7.06-7.03 (m, 1H), 6.86-6.82 (m, 3H), 4.25-4.21 (m, 1H), 4.17-4.08 (m, 3H), 4.04-4.02 (m, 2H), 3.86-3.73 (m, 4H), 1.63 (s, 6H) ppm; ^13^C NMR (150 MHz, CDCl_3_) δ 156.51, 156.11, 142.91, 142.45, 127.46, 127.40, 113.88, 113.81, 69.97, 69.44, 68.84, 68.62, 62.74, 46.80, 41.16, 30.75 ppm; ESI-HRMS: *m/z* calculated for C_21_H_27_ClKO_5_ [M + K]^+^, 433.1184; found, 433.1179.

C_18_ reversed-phase HPLC trace of **EPI-10000** (**6**) dissolved in DMSO using a InertSustain 5 µm, 25 x 1 cm column with 3:2 MeCN/H_2_O as eluent at a flow rate of 2 mL/min with UV detection at 107 and 228 nm.


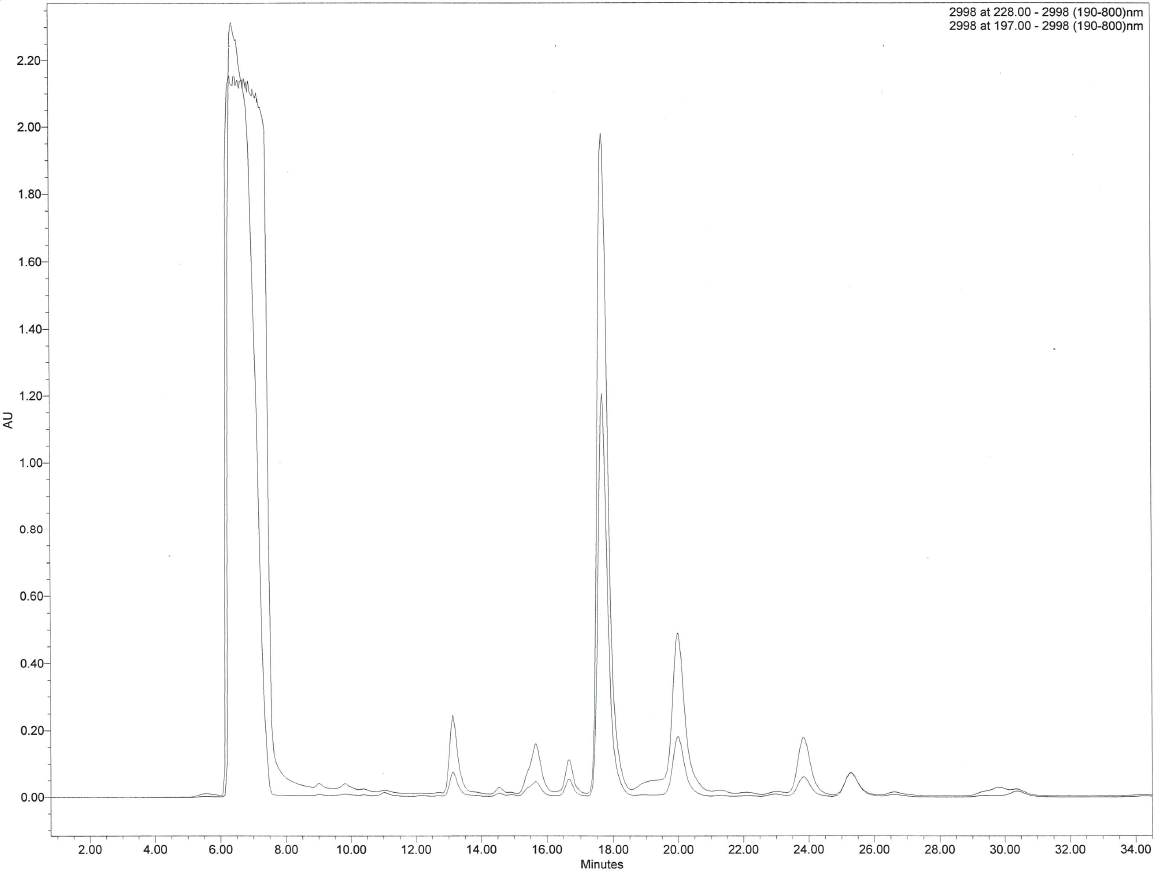


**3.7 BU-122 (7)**

¹H NMR (600 MHz, DMSO-*d_6_*) δ 7.19 (s, 2H), 7.12 (d, J = 12.0 Hz, 2H), 6.84 (d, J = 12.0 Hz, 2H), 5.02 (d, J = 6.0 Hz, 1H), 4.77 (t, J = 6.0 Hz, 1H), 4.05 (t, J = 6.0 Hz, 2H), 3.94 (dd, J = 9.7, 4.2 Hz, 1H), 3.84-3.80 (m, 3H), 3.76 (m, 1H), 3.43 (m, 2H), 2.17 (tt, J = 6.2, 6.2, 2H), 1.57 (s, 6H) ppm; ^13^C NMR (150 MHz, DMSO-*d_6_*) δ 156.68, 149.05, 148.02, 140.57, 127.64, 127.35, 127.09, 113.98, 69.99, 69.77, 69.24, 62.49, 41.64, 41.60, 32.45, 29.93 ppm; ESI-LRMS: *m/z* calculated for C_21_H_25_Cl_3_NaO_4_ [M + Na]^+^, 469.0716; found, 469.2.

^1^H NMR Spectrum of **BU-122** (**7**)) recorded at 600 MHz in DMSO-*d*_6_

_
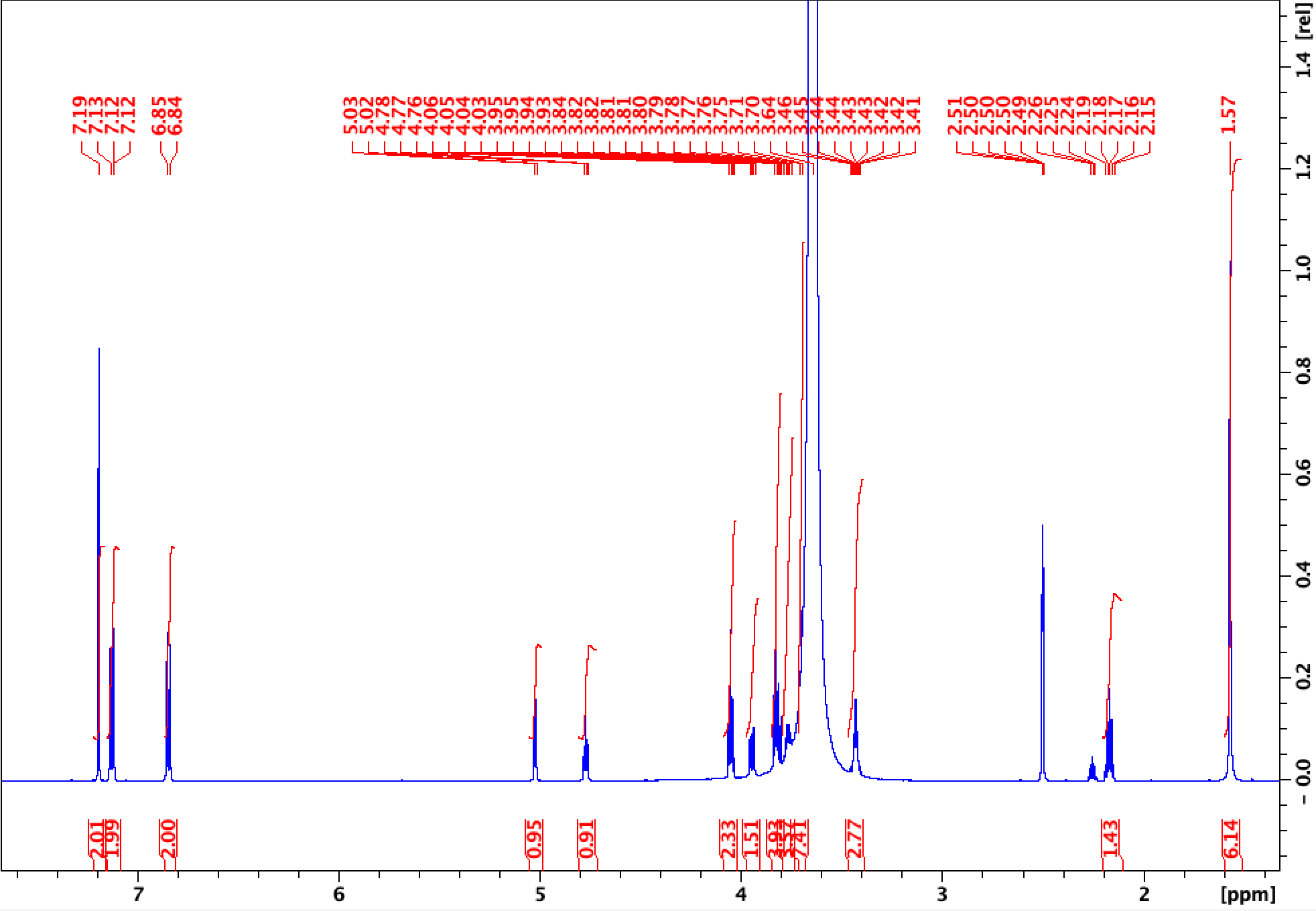
_

^13^C NMR Spectrum of **BU-122** (**7**) recorded at 150 MHz in DMSO-*d*_6_


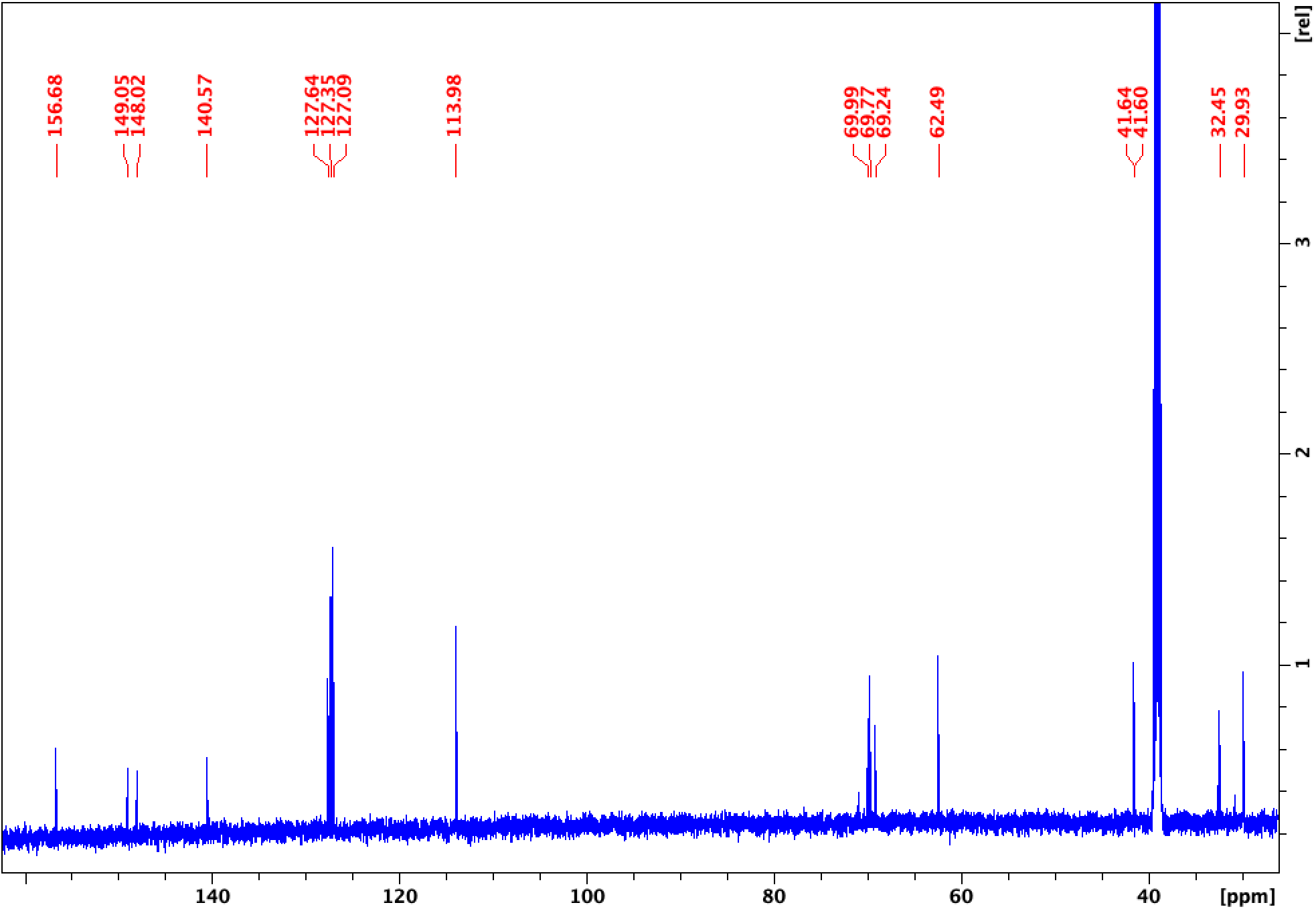


**3.8 BU1-2 (8)**

^1^H NMR (600 MHz, CDCl_3_) δ 7.14-7.13 (m, 4H), 6.86 (d, J = 9.0 Hz, 2H), 4.26-4.23 (m, 1H), 4.21-4.15 (m, 2H), 4.15-4.11 (m, 1H), 4.08-4.03 (m, 2H), 3.86 (dd, J = 10.8, 4.8Hz, 2H), 3.78 (dd, J = 12.6, 6.6 Hz, 2H), 1.64 (s, 6H) ppm; ^13^C NMR (150 MHz, CDCl_3_) δ 156.76, 149.30, 148.26, 141.84, 128.52, 127.87, 127.60, 114.35, 73.69, 70.48, 69.26, 63.78, 45.55, 42.34, 30.79 ppm; ESI-HRMS: *m*/*z* calculated for C_21_H_25_Cl_3_KO_5_ [M + K]^+^, 501.0405; found, 501.0400.

C_18_ reversed-phase HPLC trace of **BU1-2** (**8**) dissolved in DMSO using a InertSustain 5 µm, 25 x 1 cm column with 3:2 MeCN/H_2_O as eluent at a flow rate of 2 mL/min with UV detection at 197 and 228 nm.


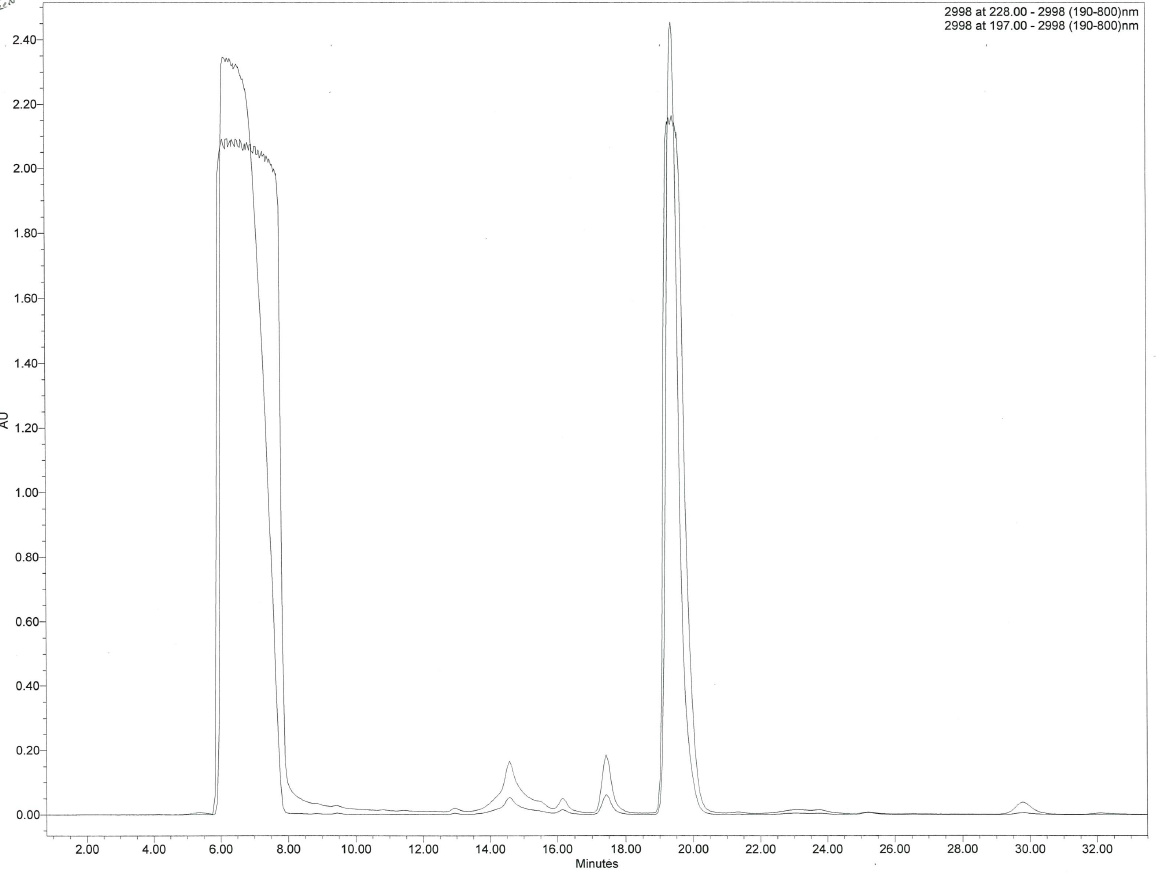


^1^H NMR Spectrum of **BU1-2** (**8**) recorded at 600 MHz in CDCl_3_


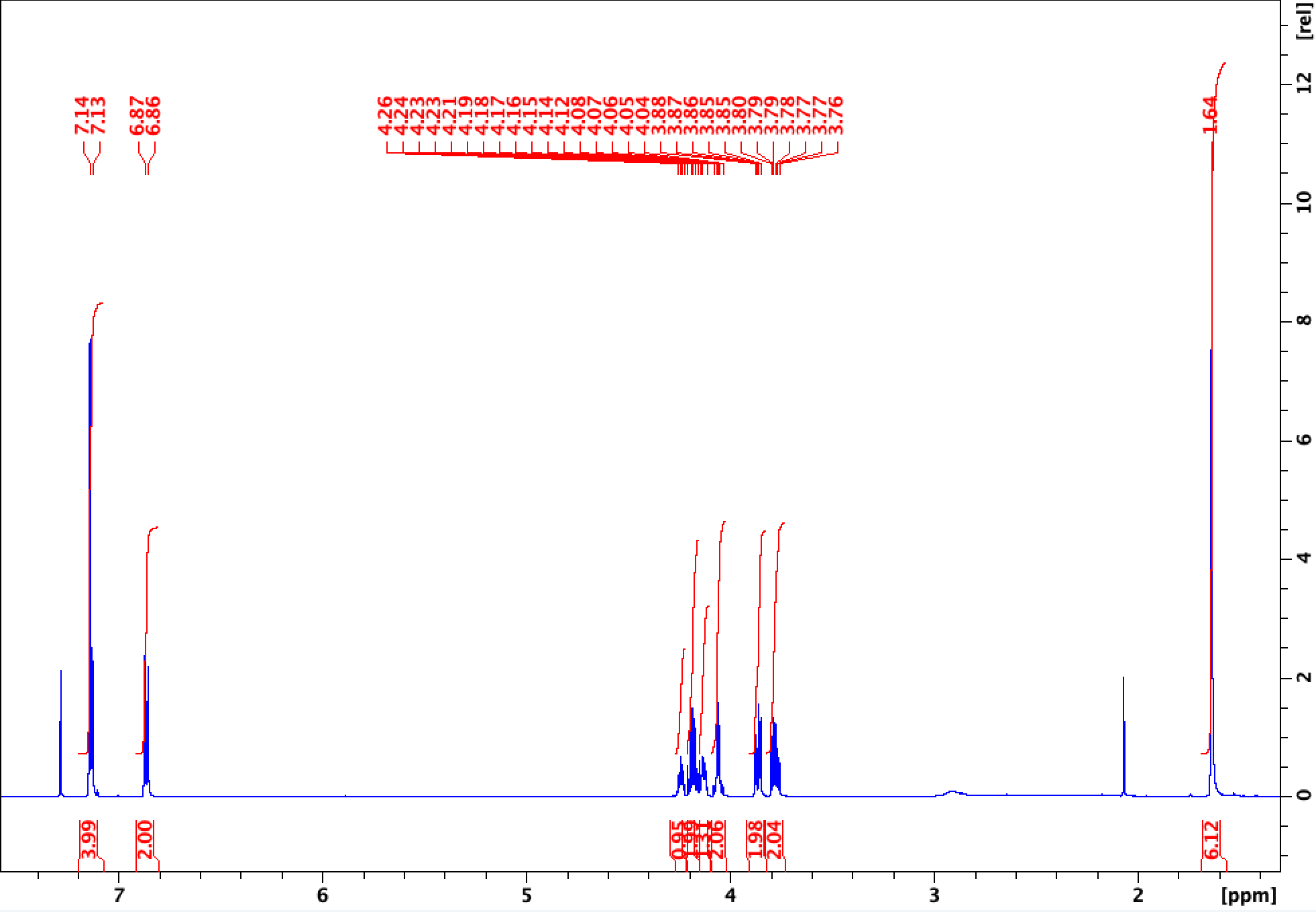


^13^C NMR Spectrum of **BU1-2** (**8**) recorded at 150 MHz in CDCl_3_


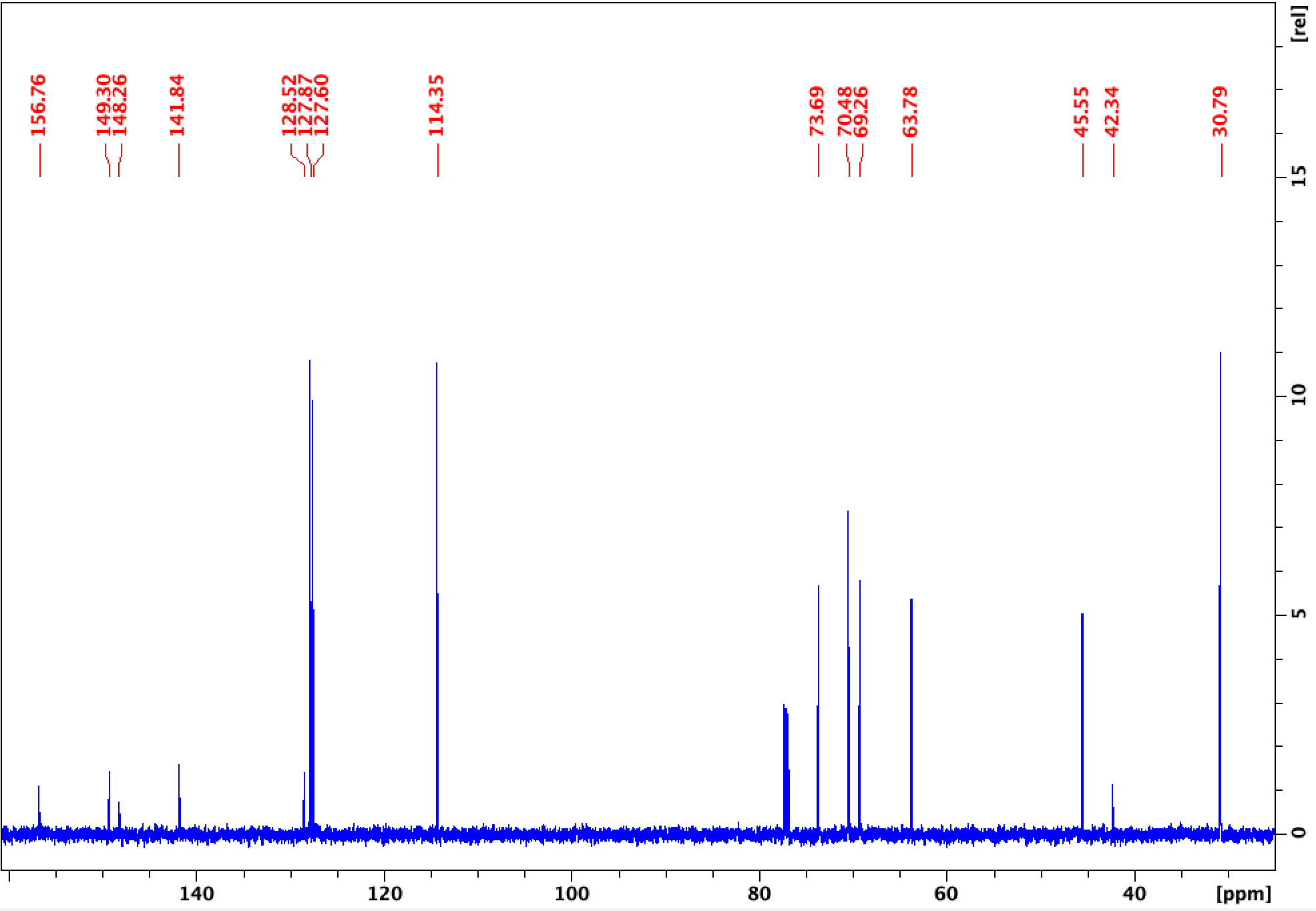


**3.9 BU-162 (9)**

^1^H NMR (400 MHz, CDCl_3_) δ 7.11-7.07 (m, 4H), 6.87 (d, J = 8.3 Hz, 2H), 4.36 (t, J = 6.72 Hz, 1H), 4.24 (t, J = 7.1 Hz, 1H), 4.16 (t, J = 5.8 Hz, 2H), 4.14-4.10 (m, 1H), 4.06-4.04 (m, 2H), 3.88-3.84 (m, 3H), 3.76 (dd, J = 11.4, 5.4 Hz, 1H), 2.50 (m, 1H), 2.45 (m, 1H), 2.28 (t, J = 5.9 Hz, 2H), 1.64 (s, 3H) ppm; ^13^C NMR (100 MHz, CDCl_3_) 157.03, 149.28, 147.28, 139.73, 136.46, 129.11, 128.36, 127.71, 114.56, 92.84, 81.60 (d, J = 164.4 Hz), 70.49, 70.05, 69.28, 63.78, 44.41 (d, J = 5.8 Hz), 41.69 (d, J = 15.1 Hz), 41.65, 33.37, 28.28 ppm.

^1^H NMR Spectrum of **BU-162** (**9**) recorded at 400 MHz in CDCl_3_


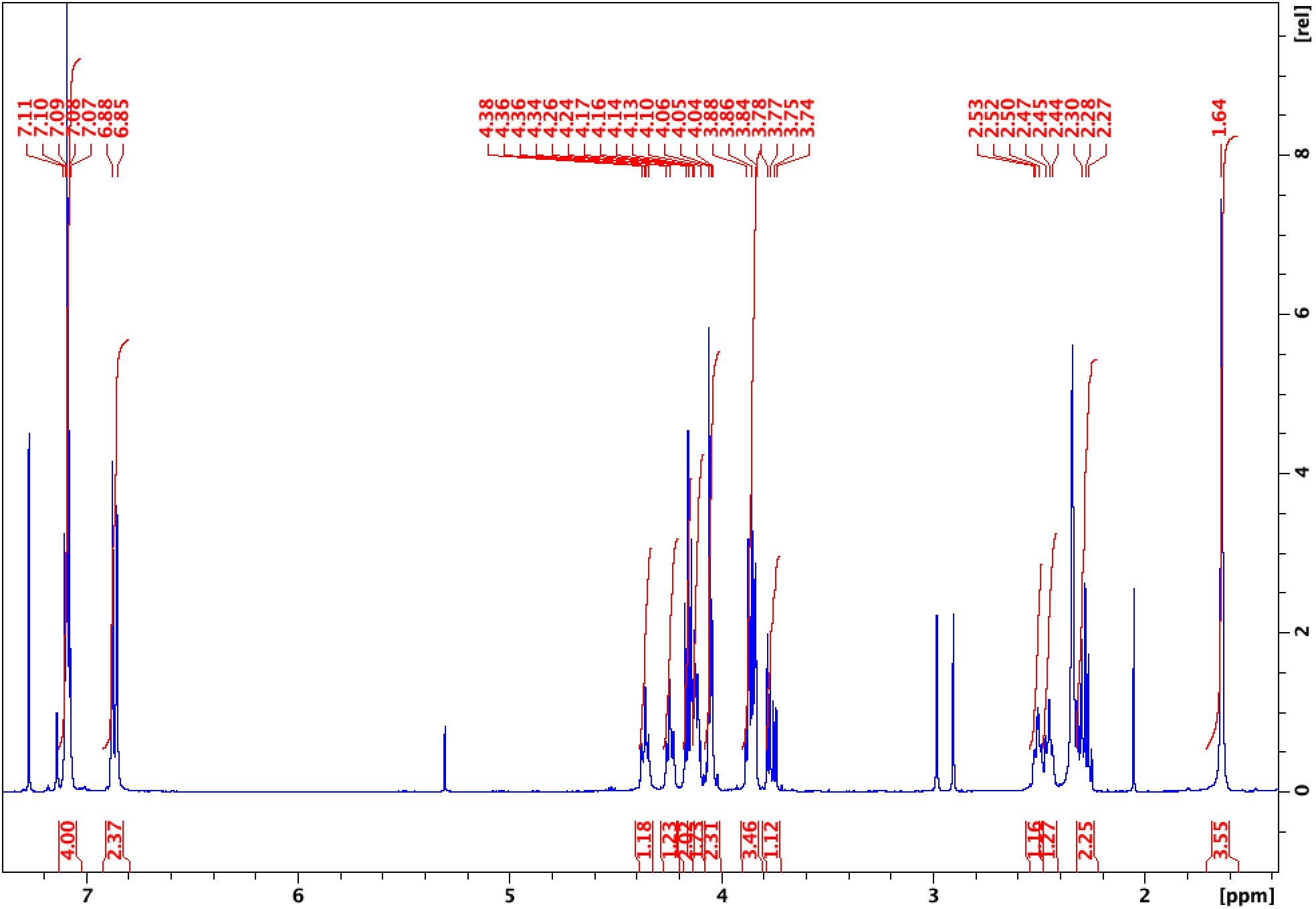


**3.10 BU-85 (10)**

¹H NMR (400 MHz, DMSO-*d_6_*) δ 7.48 (d, J = 2.3 Hz, 1H), 7.15 (dd, J = 8.6, 2.3 Hz, 1H), 7.08 (d, J = 8.8 Hz, 2H), 6.92 (d, J = 8.7 Hz, 1H), 6.84 (d, J = 8.8 Hz, 2H), 5.56 (d, J = 4.6 Hz, 1H), 4.90 (d, J = 5.1 Hz, 1H), 4.63 (t, J = 5.6 Hz, 1H), 4.33 (t, J = 6.7 Hz, 1H), 4.21 (t, J = 6.8 Hz, 1H), 4.04-4.02 (m, 2H), 3.97-3.93 (m, 2H), 3.86-3.79 (m, 2H), 3.77-3.71 (m, 2H), 3.42 (t, J = 5.8 Hz, 2H), 2.46 (t, J = 4.0 Hz, 1H), 2.44 (t, J = 6.8 Hz, 1H), 1.56 (s, 3H) ppm; ^13^C NMR (150 MHz, CDCl_3_) 156.70, 154.75, 144.41, 140.80, 137.82, 128.38, 128.11, 114.26, 111.93, 86.89, 81.75 (d, J = 164.5 Hz), 70.35, 69.75, 69.58, 69.17, 63.68, 45.72, 43.76 (d, J = 6.7 Hz), 41.60 (d, J = 19.2 Hz), 28.41 ppm; ESI-HRMS: *m/z* calculated for C_22_H_27_ClIN_3_NaO_5_ [M + Cl]^—^, 587.0270; found, 587.0267.

C_18_ reversed-phase HPLC trace of **BU-85** (**10**) dissolved in DMSO using a InertSustain 5 µm, 25 x 1 cm column with 3:2 MeCN/H_2_O as eluent at a flow rate of 2 mL/min with UV detection at 197 and 228 nm.


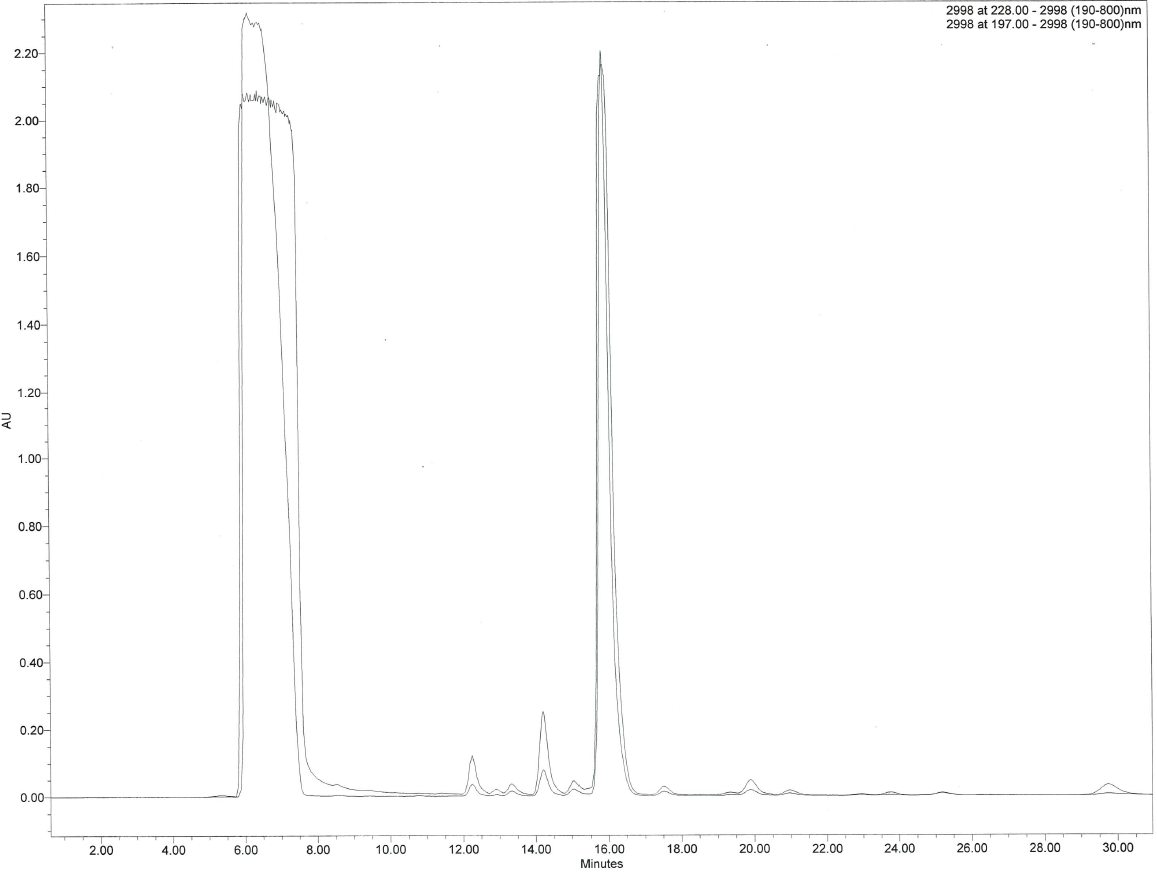


^1^H NMR Spectrum of **BU-85** (**10**) recorded at 400 MHz in DMSO-*d*_6_
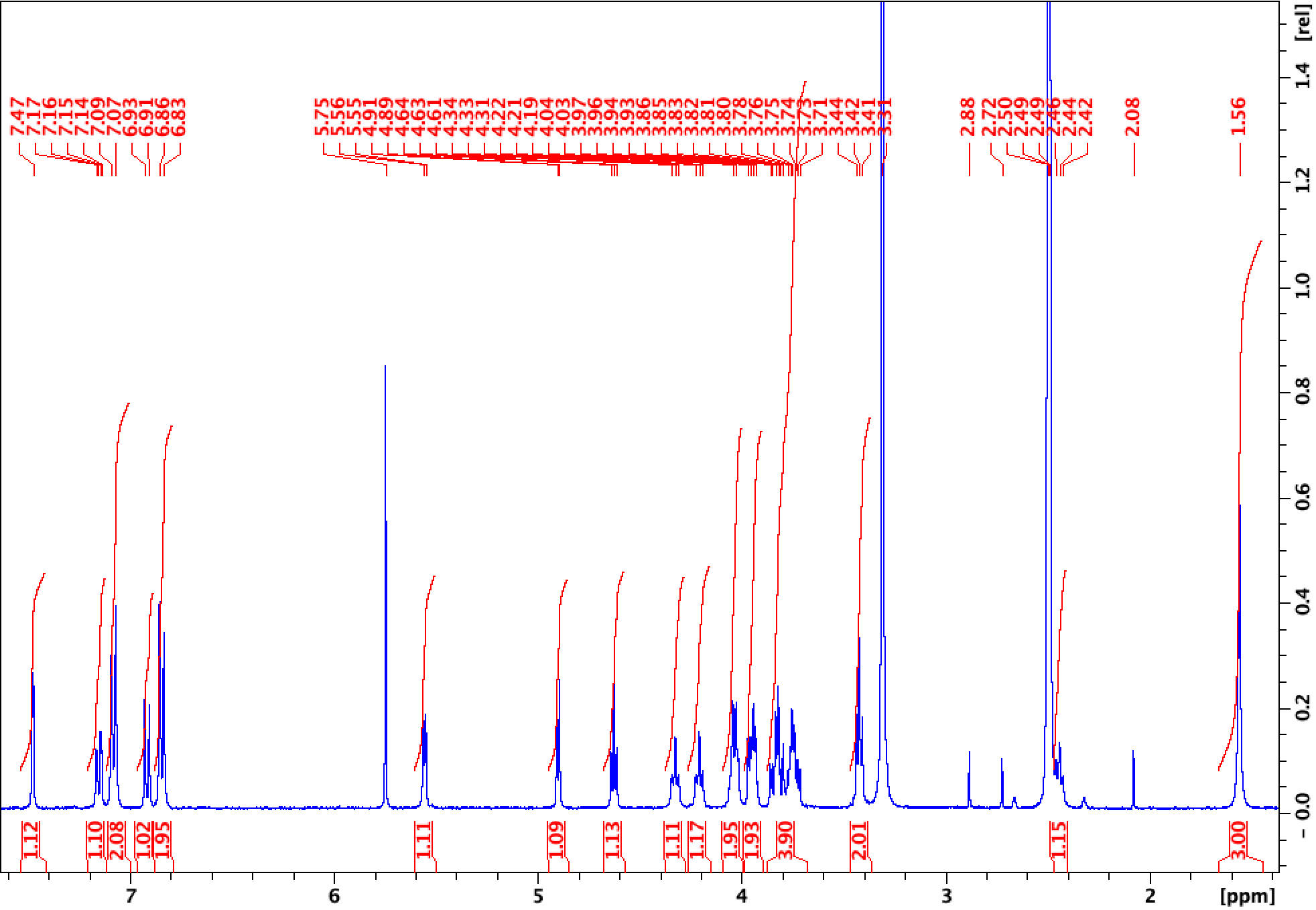


^13^C NMR Spectrum of **BU-85** (**10**) recorded at 150 MHz in CDCl_3_


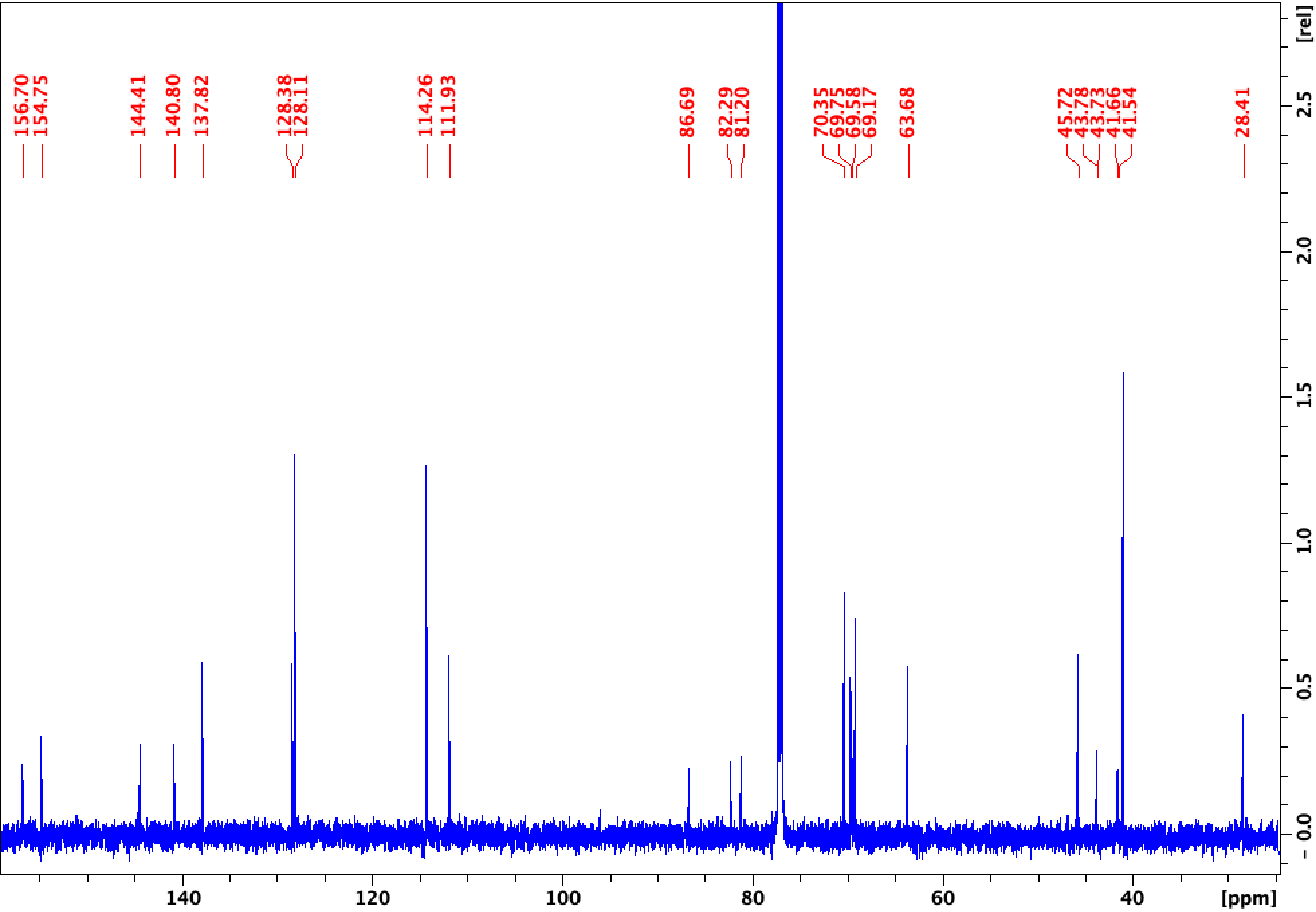


**3.11 BU4-12 (11)**

^1^H NMR (600 MHz, CDCl_3_) δ 7.56 (bs, 1H), 7.16-7.14 (m, 4H), 7.04 (bs, 1H), 7.01 (bs, 1H), 6.85 (d, J = 8.6 Hz, 2H), 4.29-4.23 (m, 3H), 4.22-4.13 (m, 3H), 3.98-3.92 (m, 2H), 3.87 (dd, J = 11.3, 5.6 Hz, 1H), 3.79 (dd, J = 11.0, 5.4 Hz, 1H), 1.65 (s, 6H) ppm; ESI-HRMS: *m/z* calculated for C_24_H_28_Cl_3_N_2_O_4_ [M + H]^+^, 513.1115; found 513.1107.

^1^H NMR Spectrum of **BU4-12** (**11**) recorded at 600 MHz in CDCl_3_


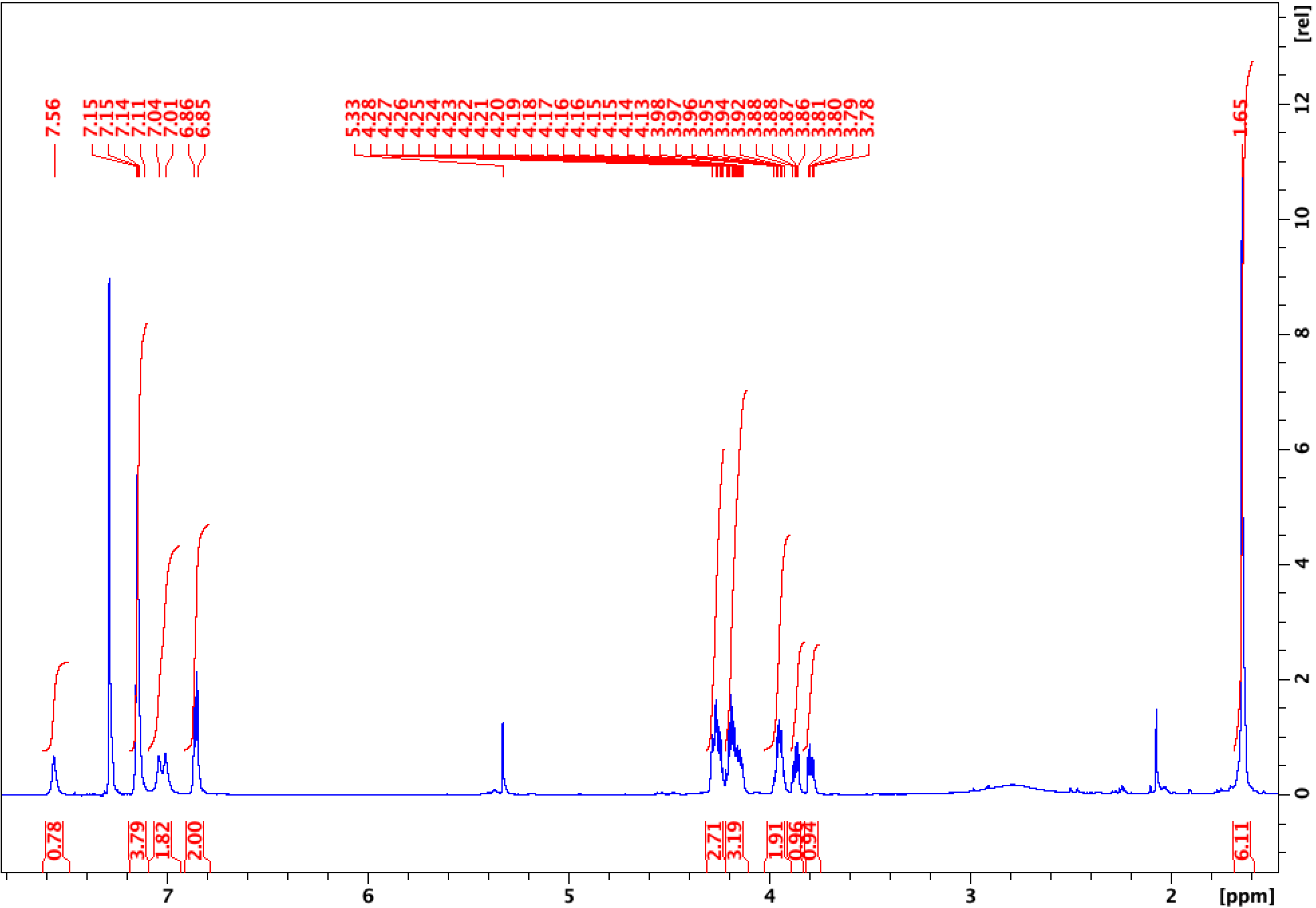


**3.12 BU6-2 (12)**

^1^H NMR (400 MHz, DMSO-*d_6_*) δ 7.17 s, 2H), 7.11 (d, J = 8.8 Hz, 2H), 6.84 (d, J = 8.8 Hz, 2H), 5.64 (d, J = 5.4 Hz, 1H), 4.95 (bs, 1H), 4.04 (m, 1H), 3.96-3.89 (m, 3H), 3.93 (dd, J = 5.6, 1.7 Hz, 1H), 3.82-3.77 (m, 3H), 3.69 (obscured m, 2H), 3.53 (t, J = 4.6 Hz, 4H), 2.41 (dd, J = 12.6, 5.3 Hz, 1H), 2.39 (m, 2H), 2.33 (dd, J = 12.8, 6.7 Hz, 1H), 1.56 (s, 6H) ppm; ^13^C NMR (600 MHz DMSO-*d_6_*) δ 157.10, 149.45, 148.47, 141.04, 127.90, 127.79, 127.55, 114.49, 74.29, 71.10, 69.37, 66.61, 66.46, 61.65, 54.21, 46.92, 42.02, 30.36 ppm; ESI-HRMS: *m*/*z* calculated for C_25_H_33_Cl_3_NO_5_ [M + H]^+^, 532.1424; found, 532.1420.

^1^H NMR Spectrum of **BU6-2** (**12**) recorded at 600 MHz in DMSO-*d*_6_


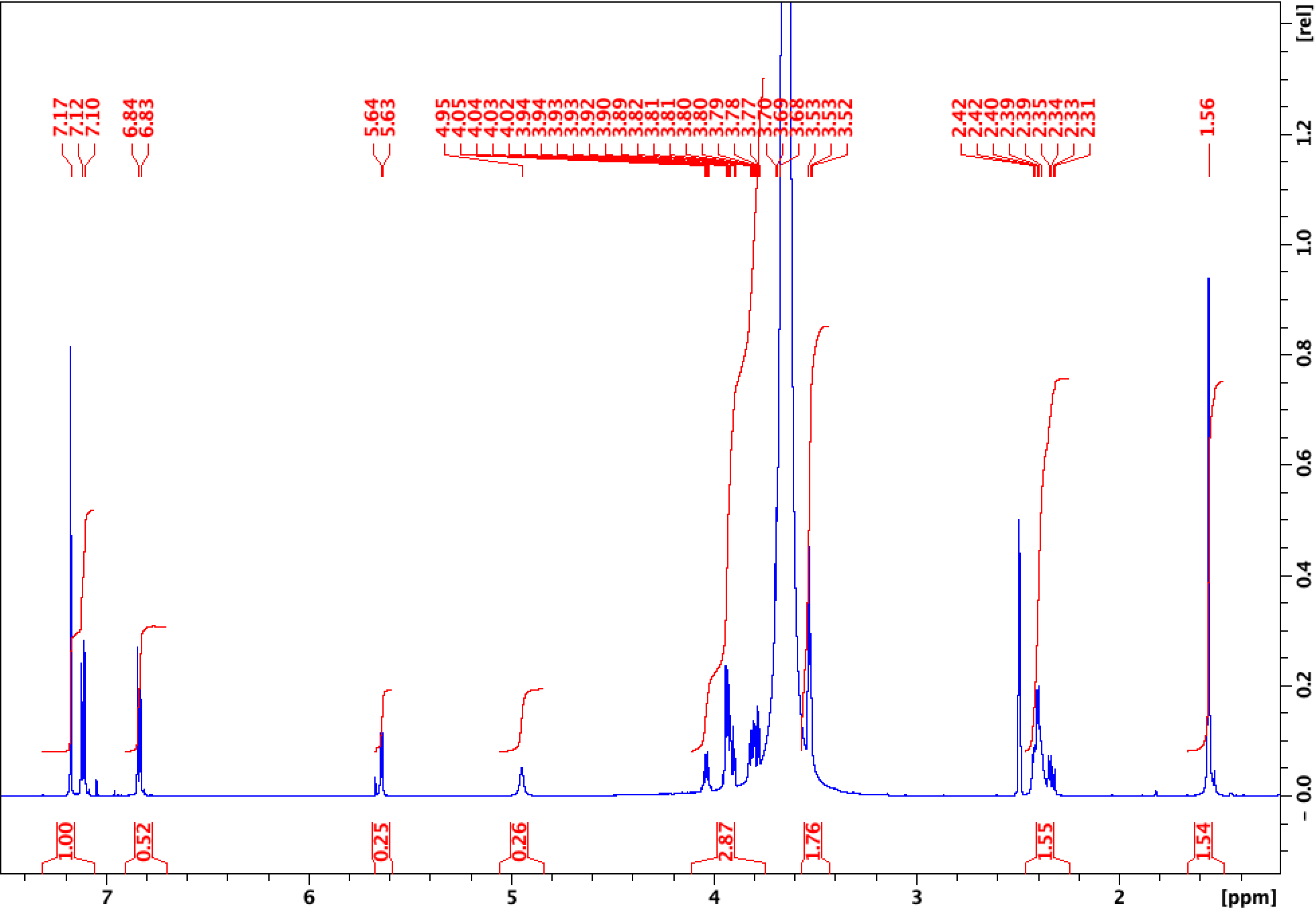


^13^C NMR Spectrum of **BU6-2** (**12**) recorded at 150 MHz in DMSO-*d*_6_


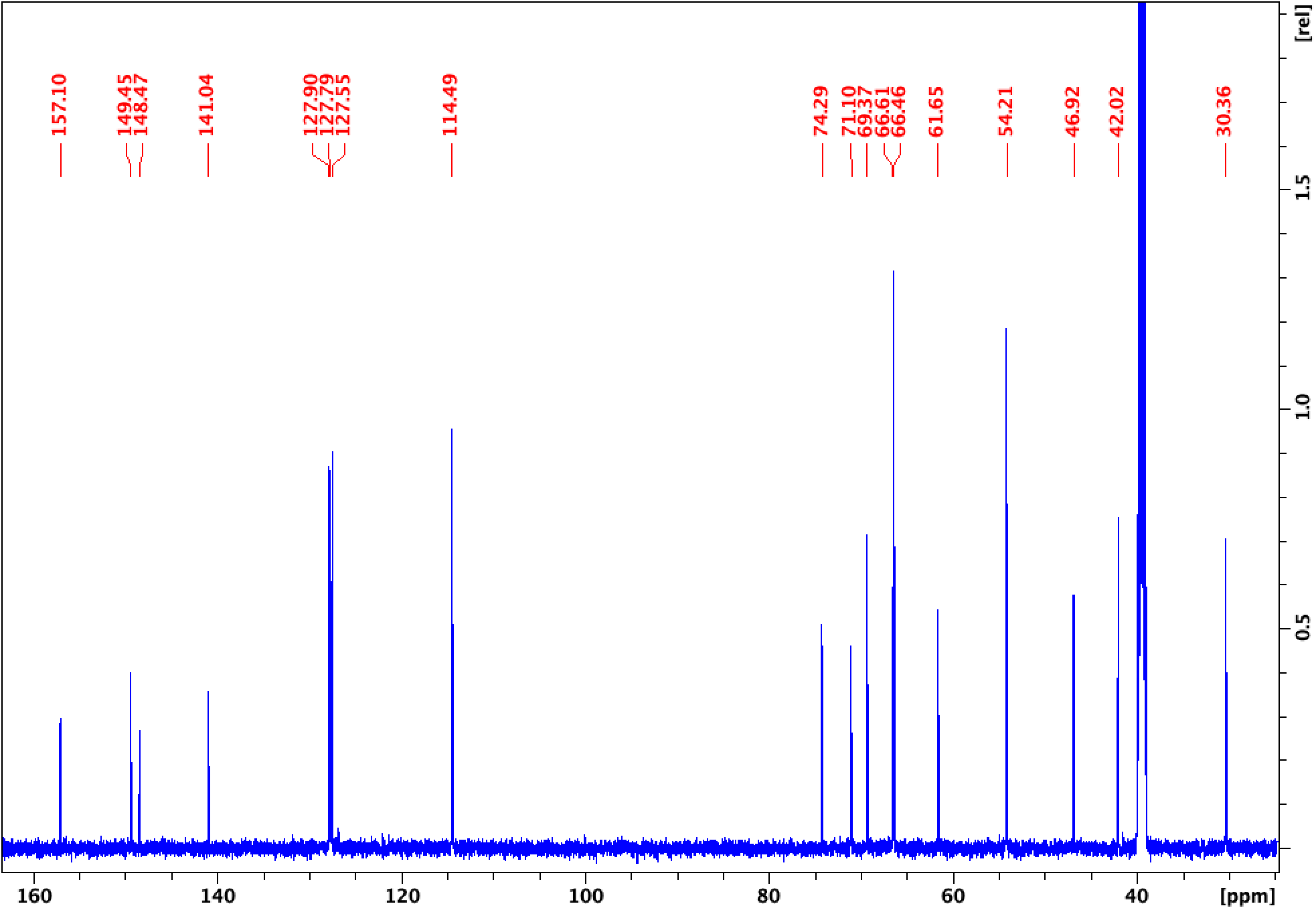


**3.13 BU-121 (13)**

¹H NMR (400 MHz, CDCl_3_) δ 7.12-7.06 (m, 4H), 6.84 (d, J = 8.0 Hz, 2H), 4.69 (s, 1H), 4.64-4.53 (m, 4H), 4.43 (s, 2H), 4.14 (t, J = 8.0 Hz, 2H), 4.10-4.02 (m, 2H), 3.85 (t, J = 8.0 Hz, 2H), 2.27 (m, 2H), 1.60 (s, 6H) ppm; ESI-HRMS: *m*/*z* calculated for C_24_H_28_Cl_3_IN_3_O_4_ [M + H]^+^, 654.0185; found, 654.0181.

C_18_ reversed-phase HPLC trace of **BU121** (**13**) dissolved in DMSO using a InertSustain 5 µm, 25 x 1 cm column with 3:2 MeCN/H_2_O as eluent at a flow rate of 2 mL/min with UV detection at 197 and 228 nm.


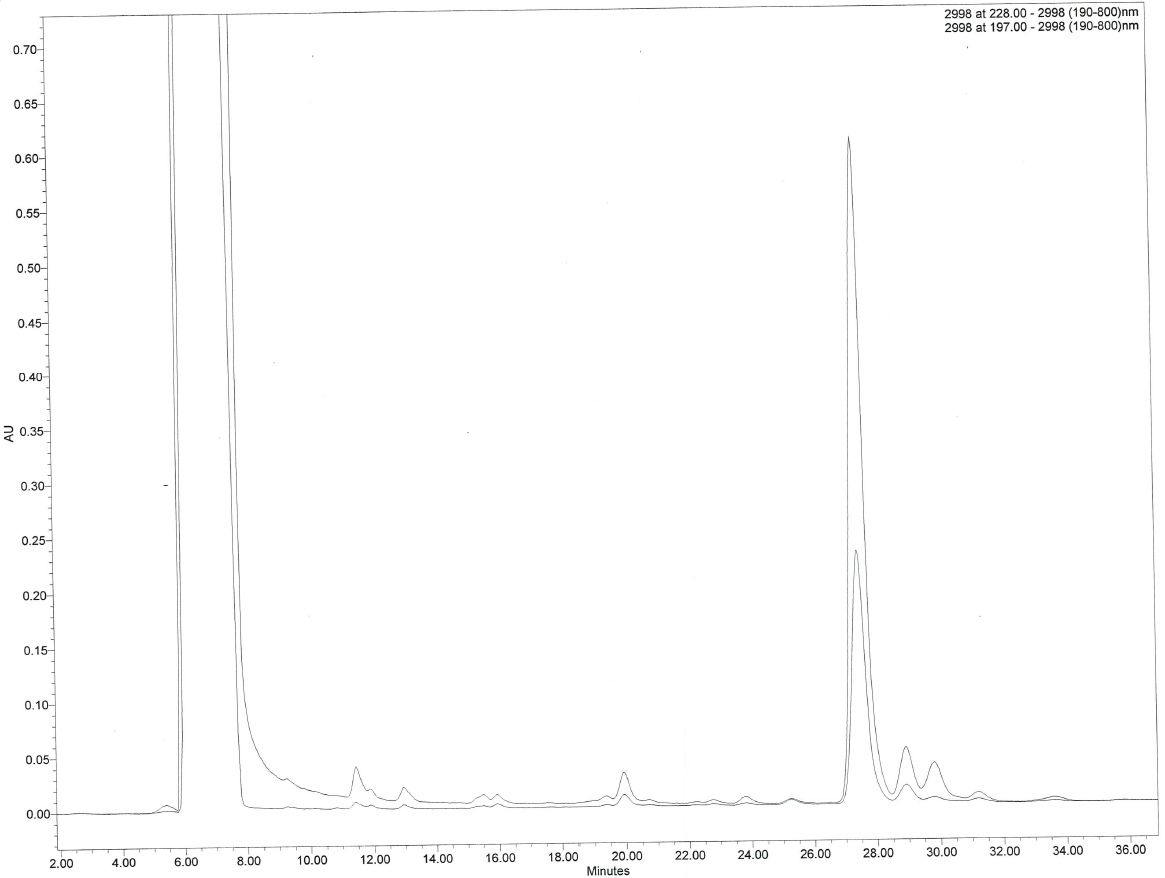


^1^H NMR Spectrum of **BU-121** (**13**) recorded at 400 MHz in CDCl_3_


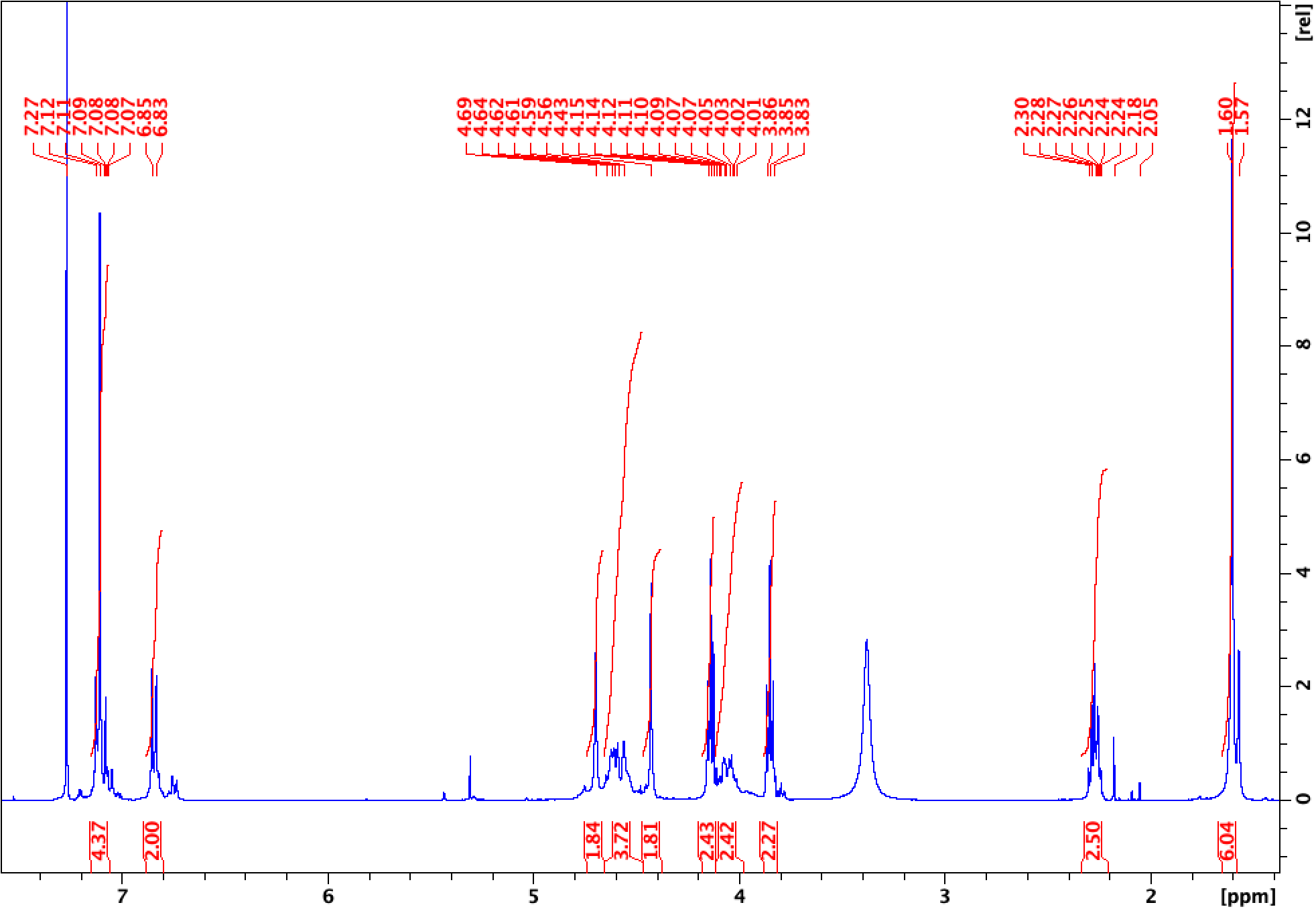


**3.14 BU-100 (14)**

¹H NMR (600 MHz, DMSO-*d_6_*) δ 7.20 (s, 2H), 7.15 (d, J = 8.8 Hz, 2H), 6.86 (d, J = 8.8 Hz, 2H), 5.65 (d, J = 5.4 Hz, 1H), 5.60 (d, J = 5.6 Hz, 1H), 5.26 (t, J = 5.4 Hz, 1H), 4.53 (dd, J = 14.0, 4.7 Hz, 1H), 4.43 (d, J = 5.4 Hz, 2H), 4.41 (dd, J = 14.3, 7.7 Hz, 1H), 4.28 (m, 1H), 4.05 (m, 1H), 3.97-3.94 (m, 3H), 3.92 (dd, J = 10.2, 5.3 Hz, 1H), 3.81 (d, J = 11.2, 4.5 Hz, 1H), 3.71-3.69 (obscured m, 1H), 1.58 (s, 6H) ppm; ^13^C NMR (150 MHz, DMSO-*d_6_*) δ 156.85,150.80, 149.55, 148.64, 141.56, 128.06, 128.01, 127.72, 114.69, 84.74, 74.46, 69.84, 69.53, 68.34, 55.30, 53.13, 47.07, 42.21, 30.51 ppm; ESI-HRMS: *m/z* calculated for C_24_H_27_Cl_3_IN_3_NaO_5_ [M + Na]^+^, 691.9959; found, 691.9951.

C_18_ reversed-phase HPLC trace of **BU-100** (**14**) dissolved in DMSO using a InertSustain 5 µm, 25 x 1 cm column with 3:2 MeCN/H_2_O as eluent at a flow rate of 2 mL/min with UV detection at 197 and 228 nm.


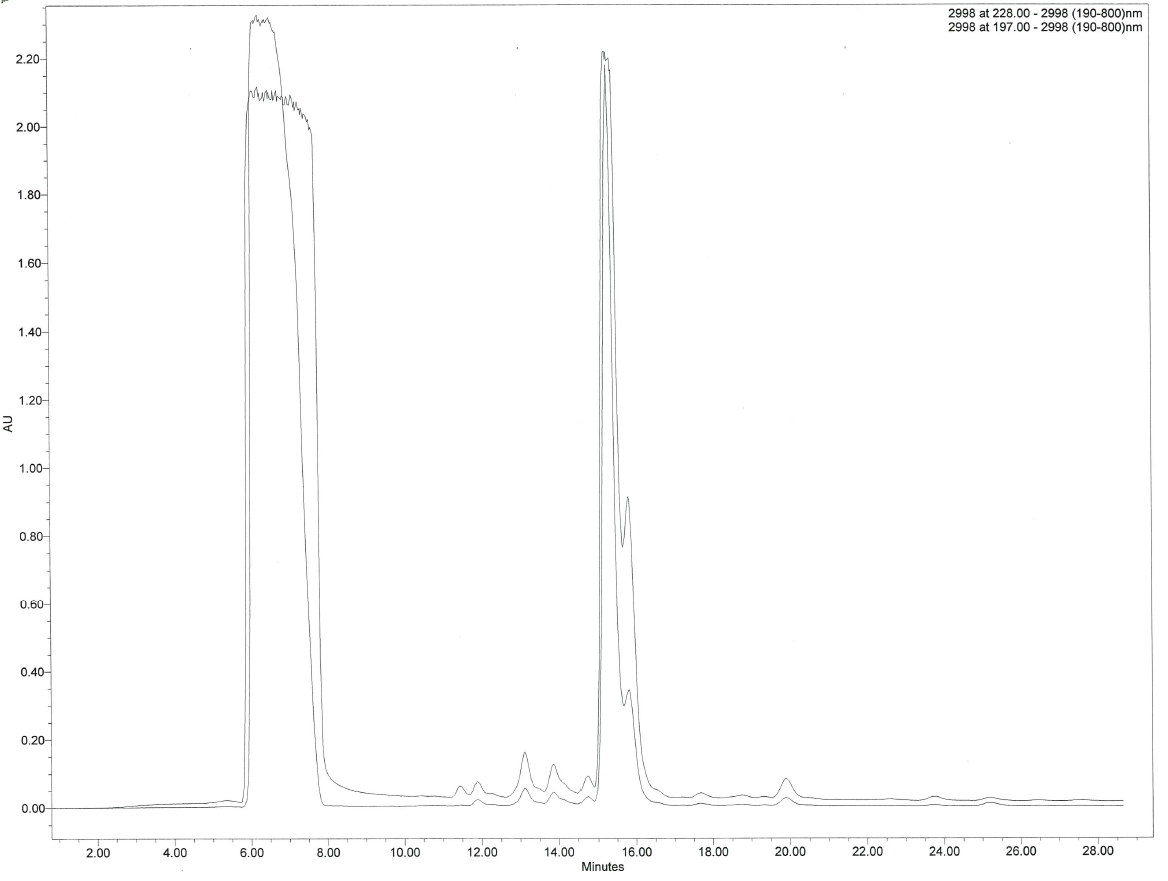


^1^H NMR Spectrum of **BU-100** (**14**) recorded at 600 MHz in DMSO-*d*_6_**
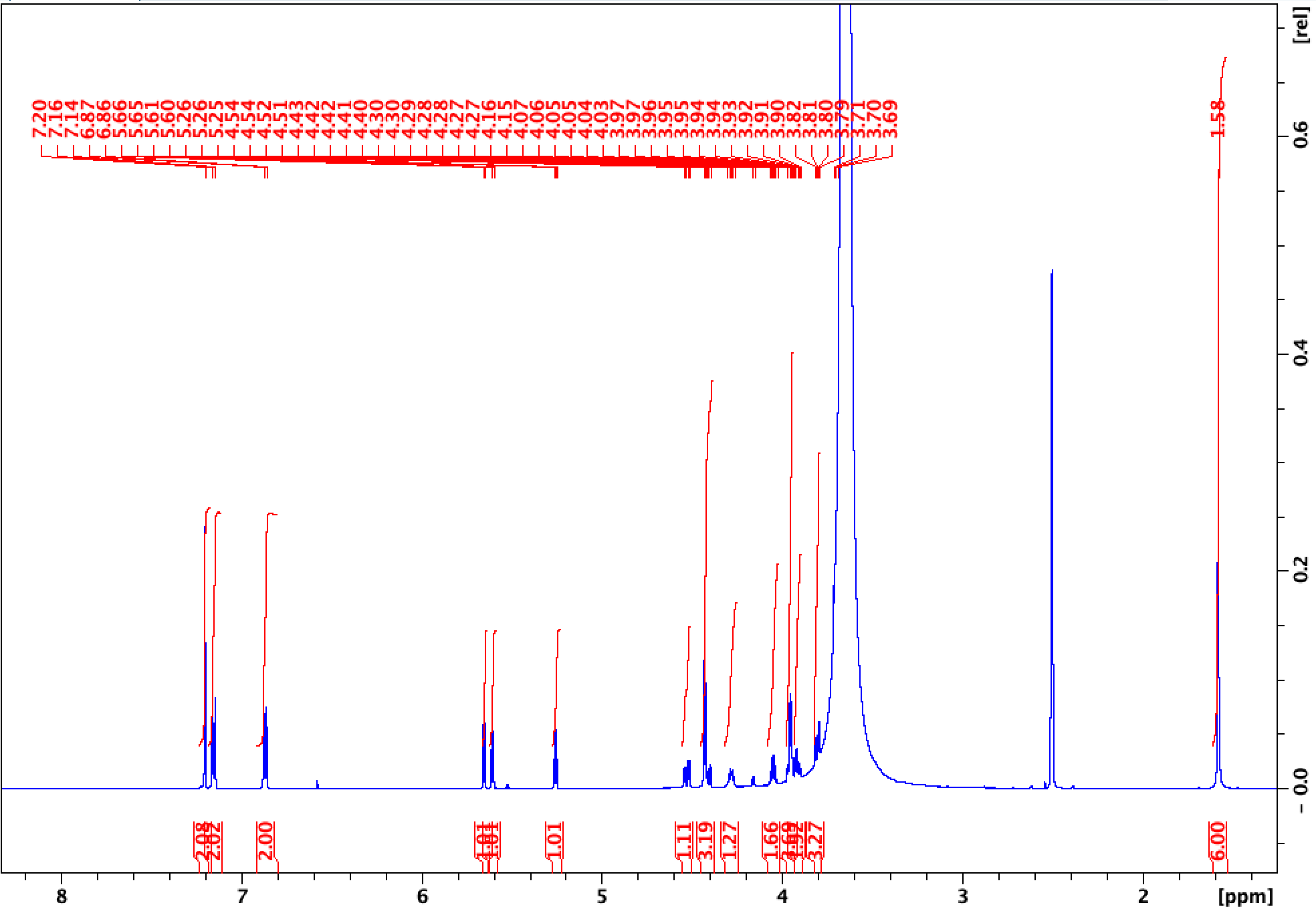
**

^13^C NMR Spectrum of **BU-100** (**14**) recorded at 150 MHz in DMSO-*d*_6_


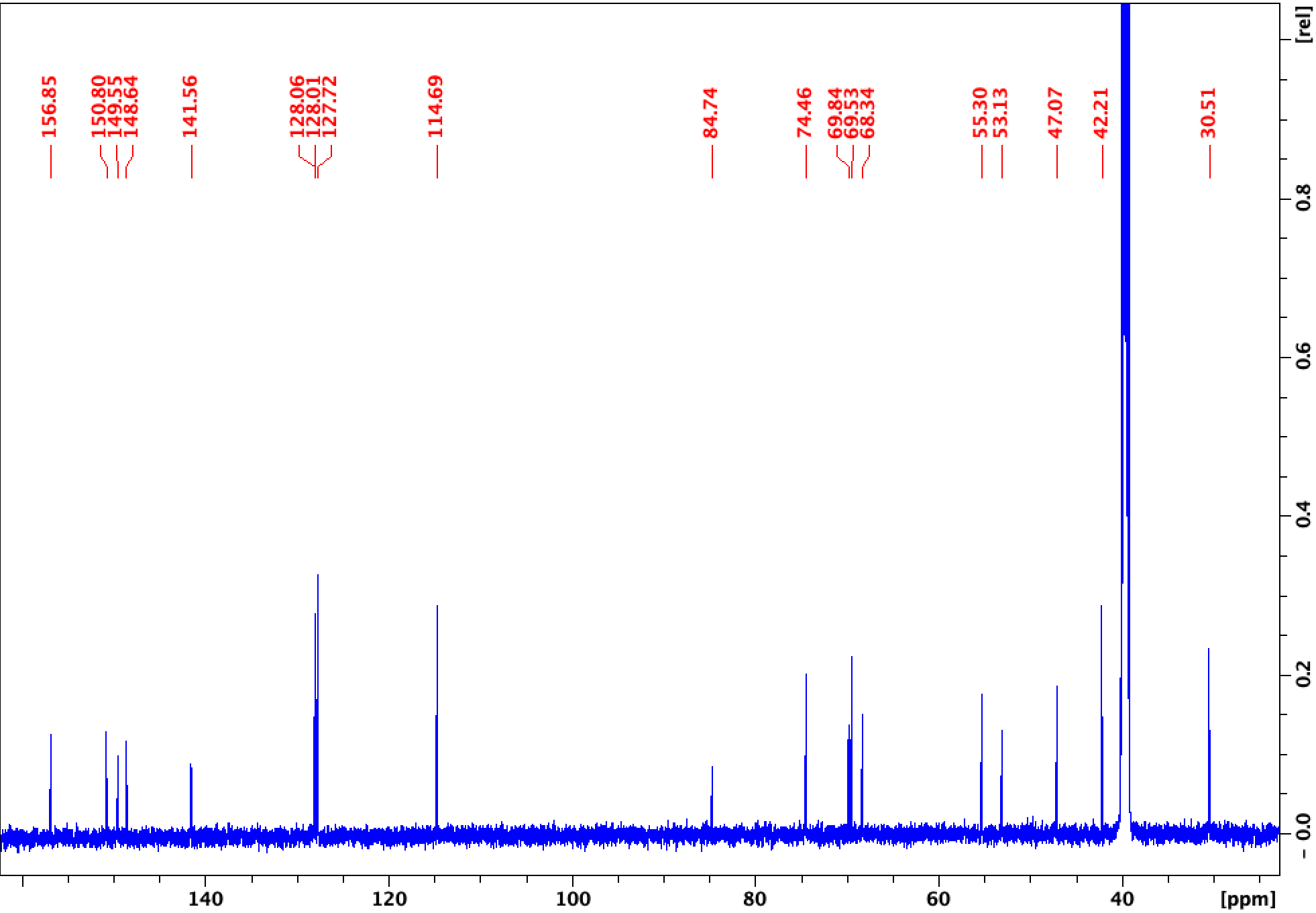


**3.15 BU5-12 (15)**

^1^H NMR (600 MHz, CDCl_3_) δ 7.14 (s, 2H), 7.13 (d, J = 8.8 Hz, 2H), 6.87 (d, J = 8.9 Hz, 2H), 4.24 (m, 1H), 4.21-4.16 (m, 3H), 4.03 (m, 2H), 3.87 (dd, J = 11.2, 5.6 Hz, 1H), 3.79 (dd, J = 11.2, 5.5 Hz, 1H), 3.61 (dd, J = 9.7, 4.3 Hz, 1H), 3.57 (dd, J = 9.7, 6.0 Hz, 1H), 3.44 (s, 3H), 1.64 (s, 6H) ppm; ^13^C NMR (150 MHz, CDCl_3_) δ: 156.37, 148.81, 147.69, 141.04, 127.95, 127.25, 127.05, 113.81, 73.13, 73.00, 69.93, 68.58, 68.44, 58.88, 45.00, 41.78, 30.25 ppm; ESI-HRMS: *m/z* calculated for C_22_H_27_Cl_3_KO_5_ [M + K]^+^, 515.0561; found, 515.0555.

^1^H NMR Spectrum of **BU5-12** (**15**) recorded at 600 MHz in CDCl_3_

**
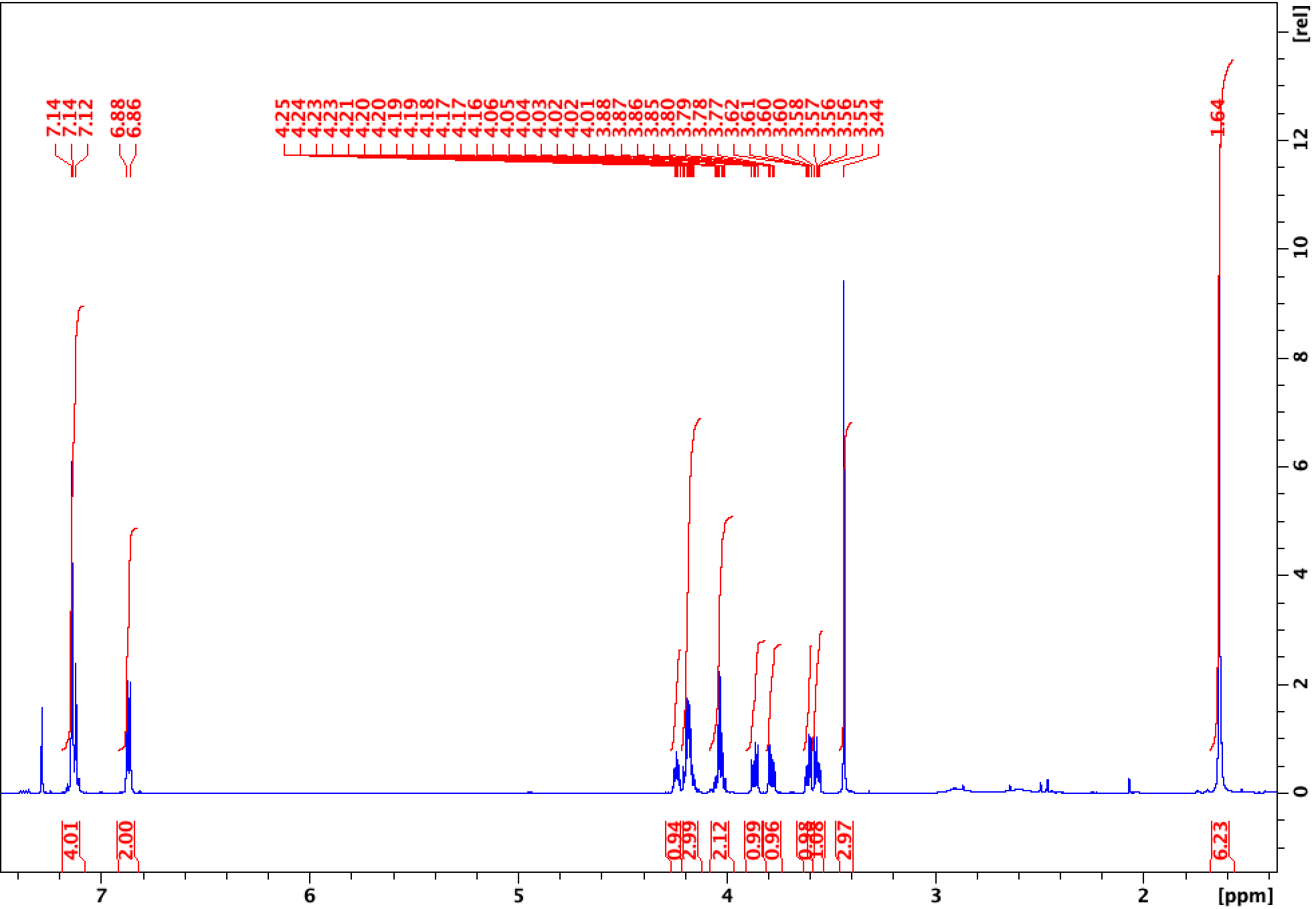
**

^13^C NMR Spectrum of **BU5-12** (**15**) recorded at 150 MHz in CDCl_3_

_
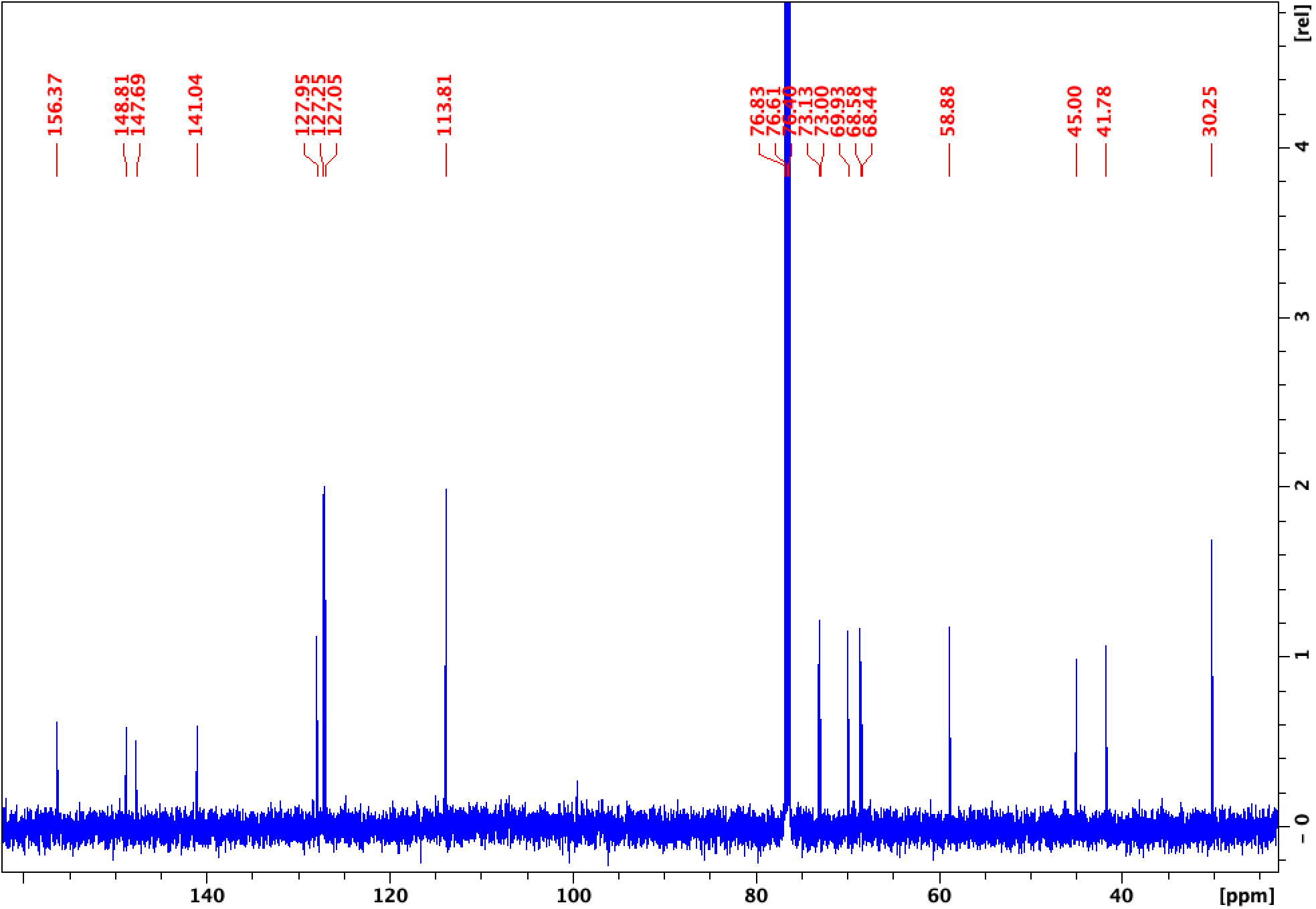
_

**3.16 BU2-12 (16)**

¹H NMR (400 MHz, DMSO-*d_6_*) δ 7.24 (s, 2H), 7.18 (d, J = 8.6 Hz, 2H), 6.89 (d, J = 8.8 Hz, 2H), 5.71 (d, J = 5.4 Hz, 1H), 5.56 (d, J = 5.4 Hz, 1H), 4.29 (m, 1H), 4.07-4.01 (m, 2H), 3.96 (m, 3H), 3.84 (dd, J = 11.2, 4.3 Hz, 1H), 3.72 (dd, J = 11.2, 5.5 Hz, 1H), 3.40 (dd, J = 14.6, 9.0 Hz, 1H), 3.24 (bd, J = 14.6 Hz, 1H), 3.05 (s, 3H), 1.61 (s, 6H) ppm; ESI-HRMS: *m/z* calculated for C_22_H_27_Cl_3_NaO_6_S [M + Na]^+^,547.0492 ; found,547.0486 .

^1^H NMR Spectrum of **BU2-12** (**16**) recorded at 600 MHz in DMSO-*d*_6_

**
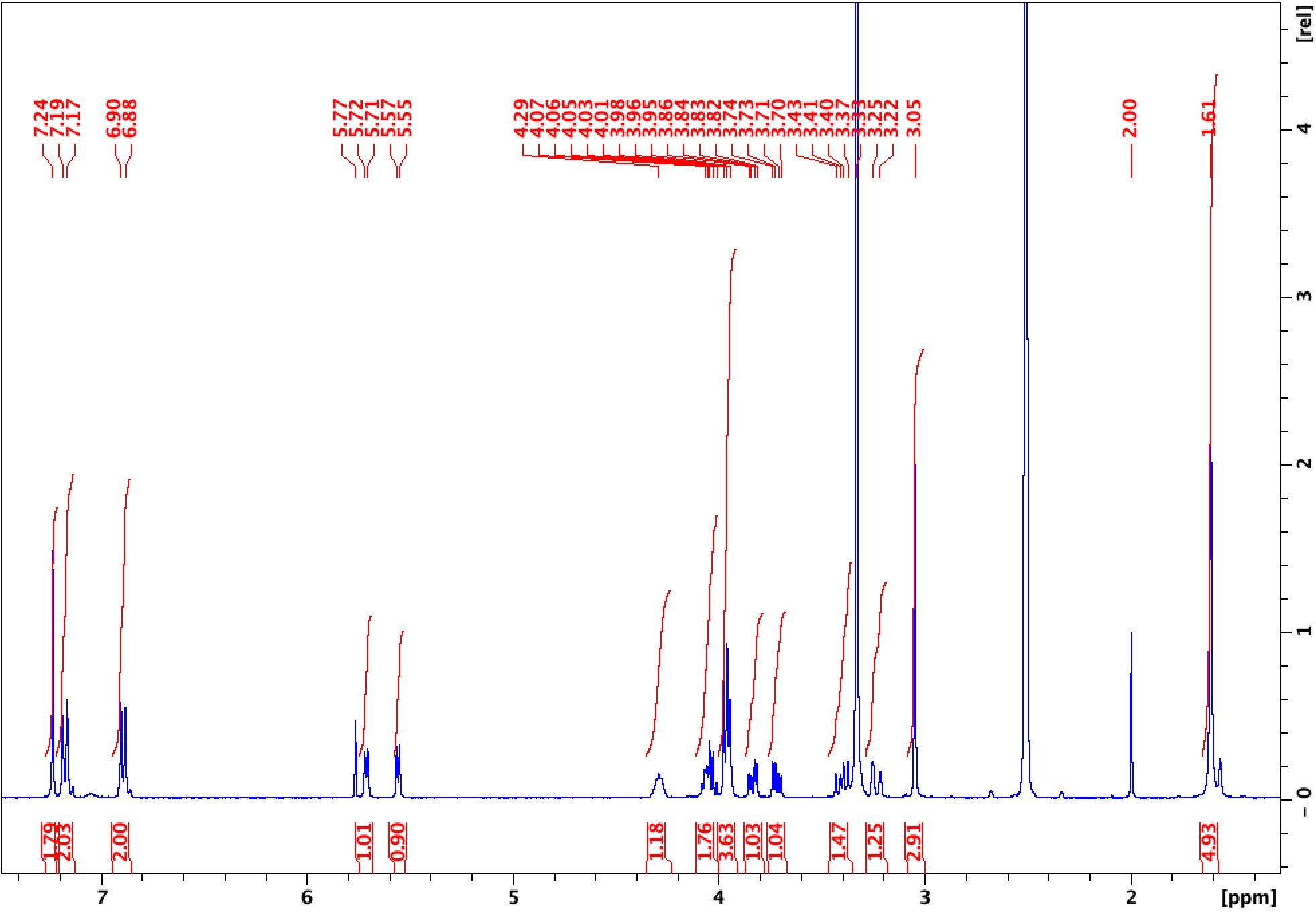
**

**3.17 BU2-22 (17)**

^1^H NMR (600 MHz, DMSO-*d_6_*) δ 7.18 (s, 2H), 7.13 (d, J = 8.9 Hz, 2H), 6.86 (d, J = 8.8 Hz, 2H), 5.75 (d, J = 5.7 Hz, 1H), 5.64 (d, J = 5.4 Hz, 1H), 4.26 (m, 1H), 4.03 (m, 1H), 3.94 (dd, J = 5.6, 2.0 Hz, 1H), 3.92 (d, J = 5.4 Hz, 2H), 3.79 (dd, J = 11.2, 4.5 Hz, 1H), 3.68 (dd, J = 11.2, 5.5 Hz, 1H), 3.33 (dd, J = 14.8, 8.8 Hz, 1H), 3.20-3.14 (m, 2H), 3.10 (m, 1H), 1.56 (s, 6H), 1.21 (t, J = 7.4 Hz, 3H) ppm; ^13^C NMR (150 MHz, DMSO-*d_6_*) δ: 156.57, 149.31, 148.41, 141.36, 127.83, 127.78, 127.49, 114.50, 74.23, 70.92, 69.30, 64.64, 55.11, 48.51, 46.85, 41.98, 30.27, 6.29 ppm; ESI-HRMS: *m/z* calculated for C_23_H_29_Cl_3_KO_6_S [M + K]^+^, 577.0387; , found, 577.0385.

^1^H NMR Spectrum of **BU2-22** (**17**) recorded at 600 MHz in DMSO-*d*_6_

**
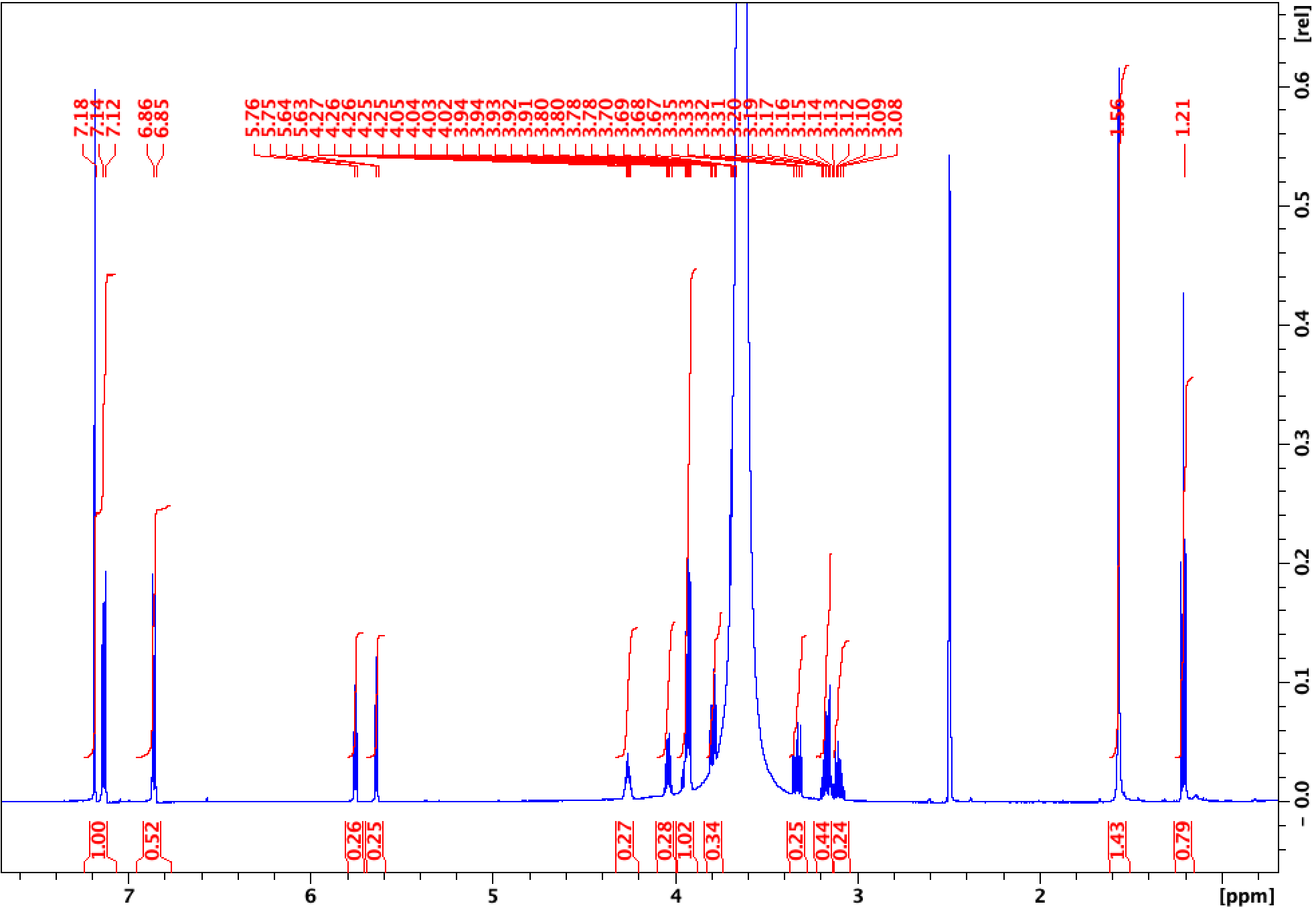
**

^13^C NMR Spectrum of **BU2-22** (**17**) recorded at 150 MHz in DMSO-*d*_6_


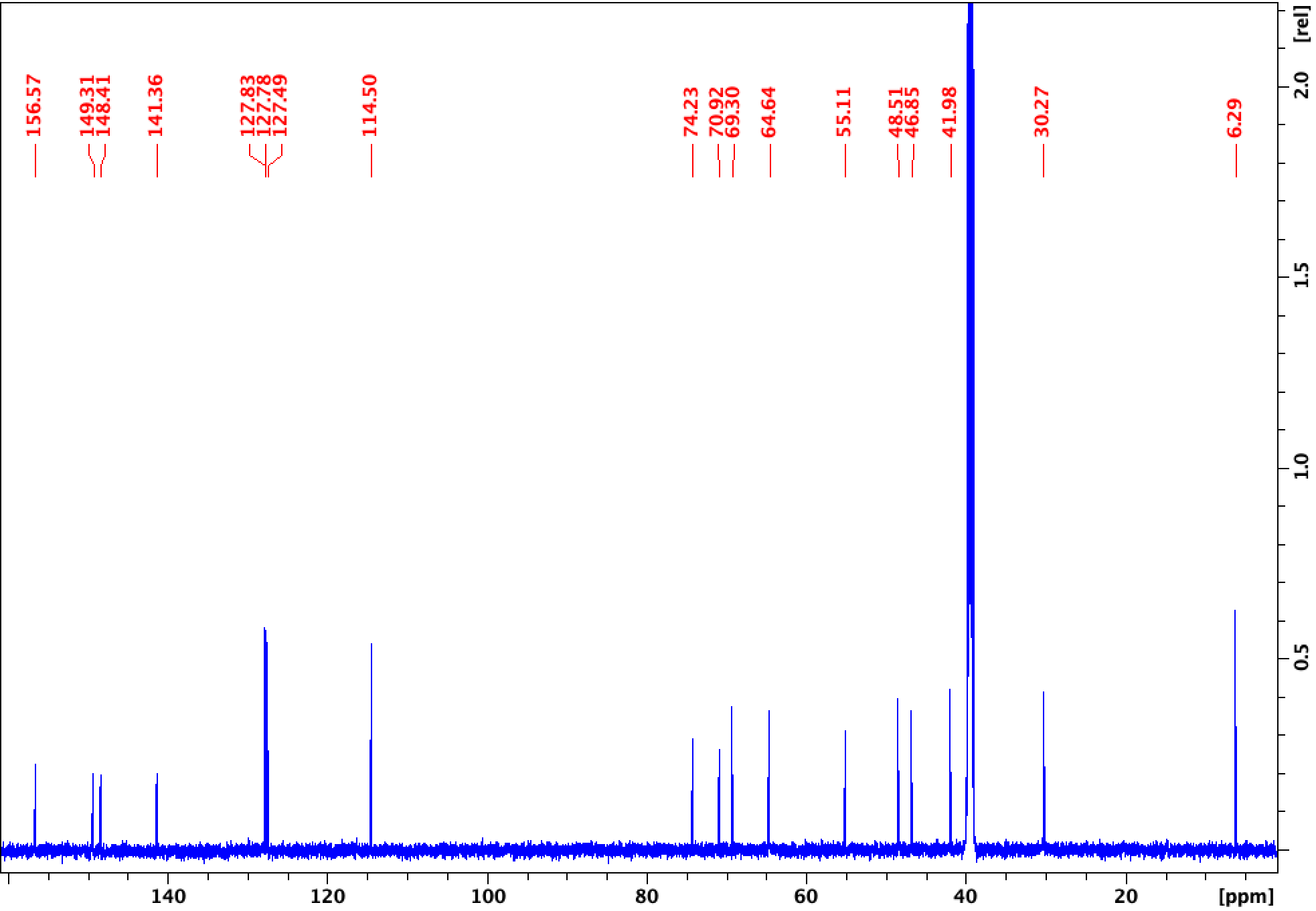


**3.18 BU-27 (18)**

¹H NMR (400 MHz, DMSO-*d_6_*) δ 7.24 (s, 2H), 7.17 (d, J = 8.8 Hz, 2H), 7.03 (t, J = 6.0 Hz, 1H), 6.88 (d, J = 8.6 Hz, 2H), 5.25 (d, J = 4.7 Hz, 1H), 4.08 (t, J = 6.0 Hz, 2H), 3.94-3.91 (m, 1H), 3.89-3.85 (m, 4H), 3.14 (m, 1H), 3.03 (m, 1H), 2.92 (s, 3H), 2.20 (m, 2H), 1.61 (s, 6H) ppm; ^13^C NMR (100 MHz, DMSO-*d_6_*) δ 156.71, 149.15, 148.21, 140.86, 127.79, 127.52, 127.30, 114.19, 70.16, 69.69, 68.21, 45.66, 41.82, 41.80, 35.80, 32.70, 30.12 ppm; ESI-HRMS: *m/z* calculated for C_22_H_28_Cl_3_KNO_5_S [M + K]^+^, 562.0391; found, 562.0387.

C_18_ reversed-phase HPLC trace of **BU-27** (**18**) dissolved in DMSO using a InertSustain 5 µm, 25 x 1 cm column with 7:3 MeCN/H_2_O as eluent at a flow rate of 2 mL/min with UV detection at 197 and 228 nm.


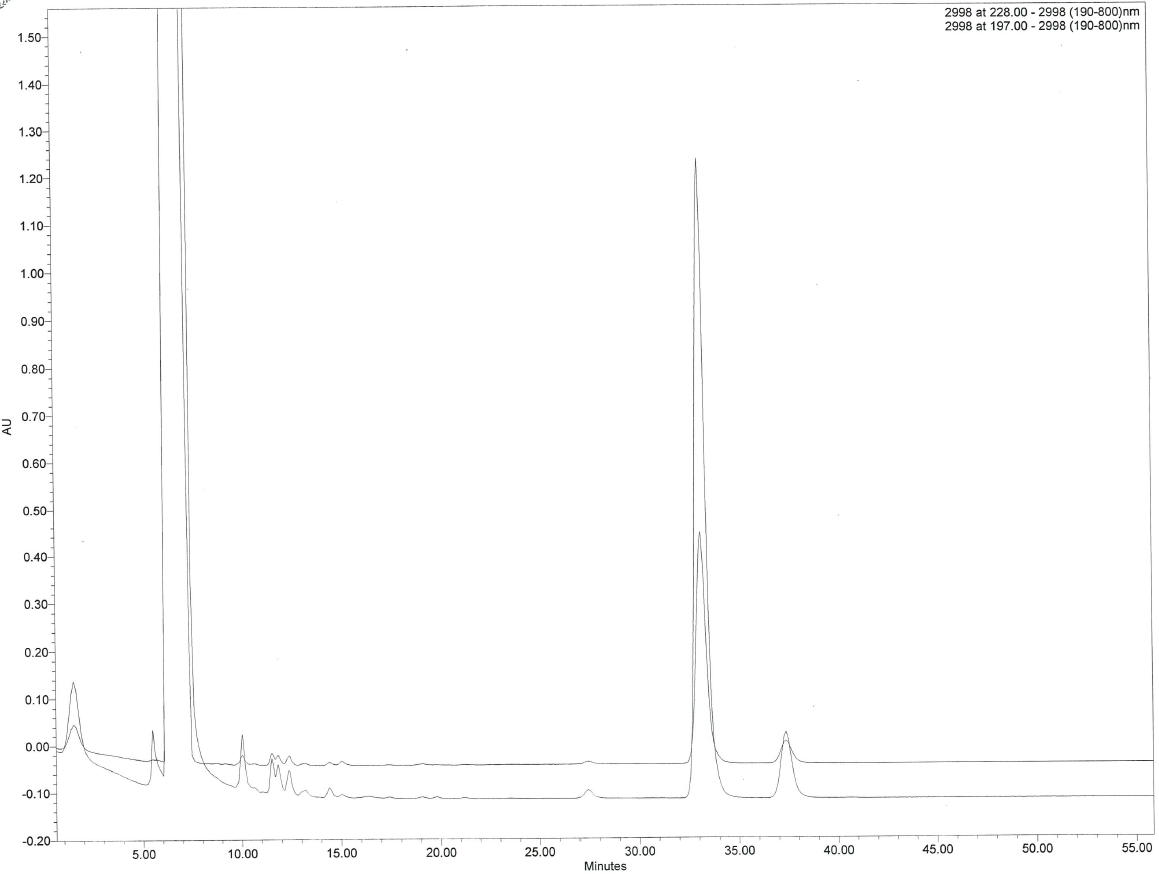


^1^H NMR Spectrum of **BU-27** (**18**) recorded at 400 MHz in DMSO-*d*_6_

_
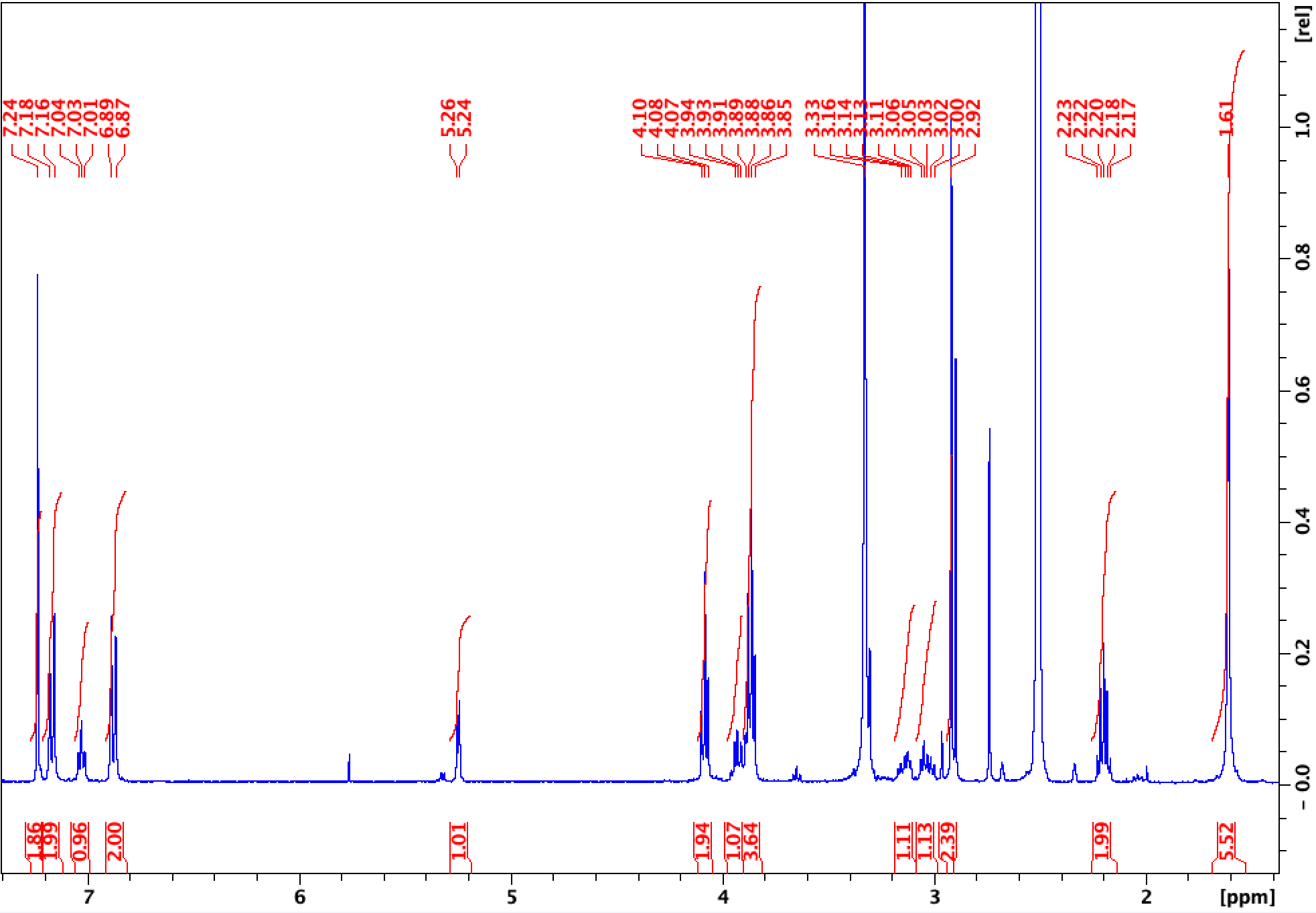
_

^13^C NMR Spectrum of **BU-27** (**18**) recorded at 100 MHz in DMSO-*d*_6_

**
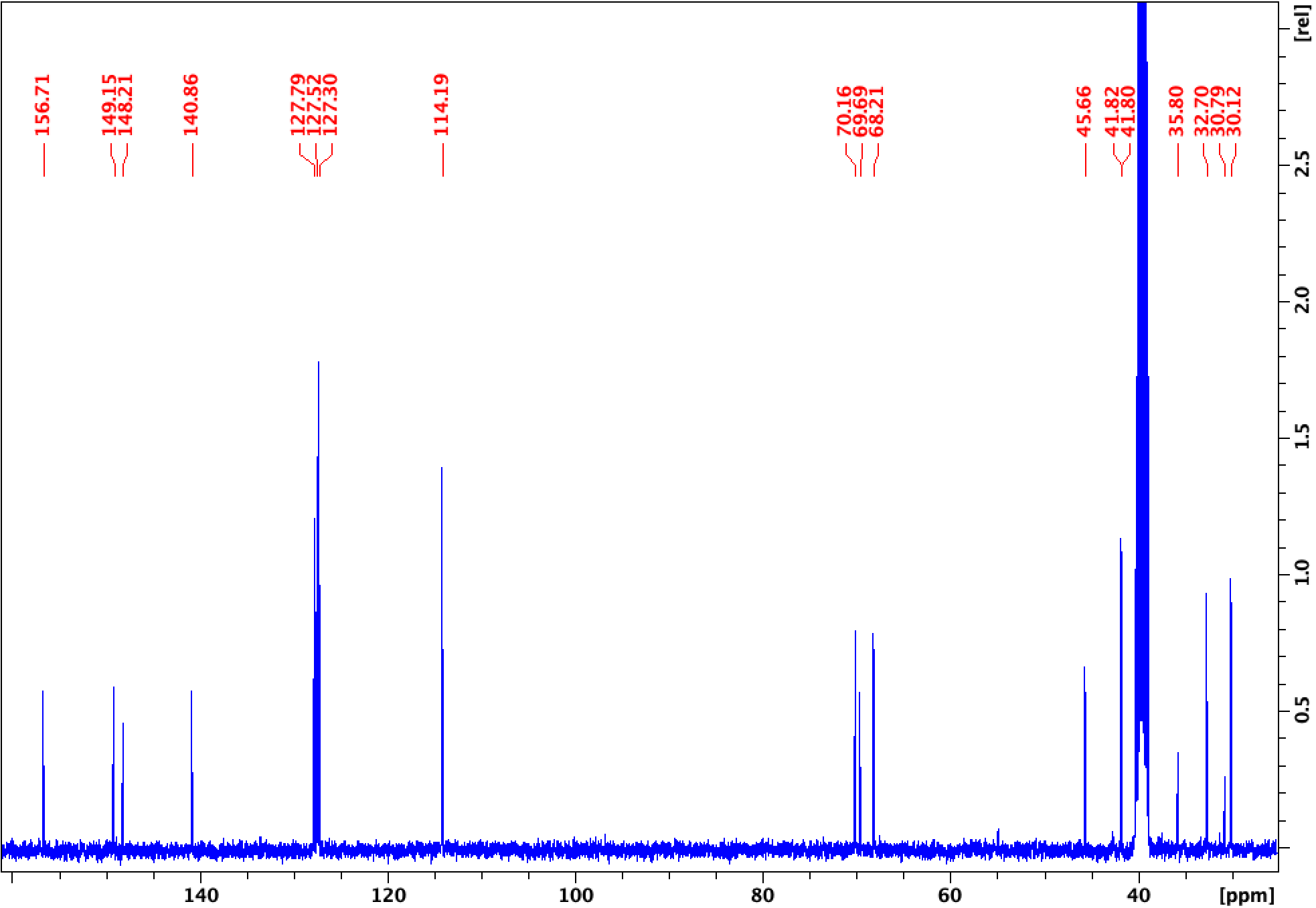
**

**3.19 BU3-12 (19)**

^1^H NMR (400 MHz, DMSO-*d_6_*) δ 7.22 (s, 2H), 7.16 (d, J = 8.6 Hz, 2H), 7.02 (t, J = 6,2 Hz, 1H), 6.87 (d, J = 8.9 Hz, 2H), 5.54 (d, J = 5.3 Hz, 1H), 5.24 (d, J = 4.8 Hz, 1H), 4.05 (m, 1H), 3.97-3.90 (m, 3H), 3.88-3.81 (m, 3H), 3.71 (dd, J = 11.2, 5.5 Hz, 1H), 3.13 (m, 1H), 3.02 (m, 1H), 2.91 (s, 3H), 1.60 (s, 6H) ppm; ^13^C NMR (150 MHz, CDCl_3_) δ 155.94, 148.69, 147.73, 141.57, 127.99, 127.39, 127.05, 113.81, 73.14, 69.92, 68.85, 68.54, 45.19, 44.99, 41.81, 39.98, 30.23 ppm; ESI-HRMS: m/z calculated for C_22_H_28_Cl_3_KNO_6_S [M+K]^+^, 578.0340; found, 578.0332.

C_18_ reversed-phase HPLC trace of **BU3-12** (**19**) dissolved in DMSO using a InertSustain 5 µm, 25 x 1 cm column with 3:2 MeCN/H_2_O as eluent at a flow rate of 2 mL/min with UV detection at 202 and 276 nm.


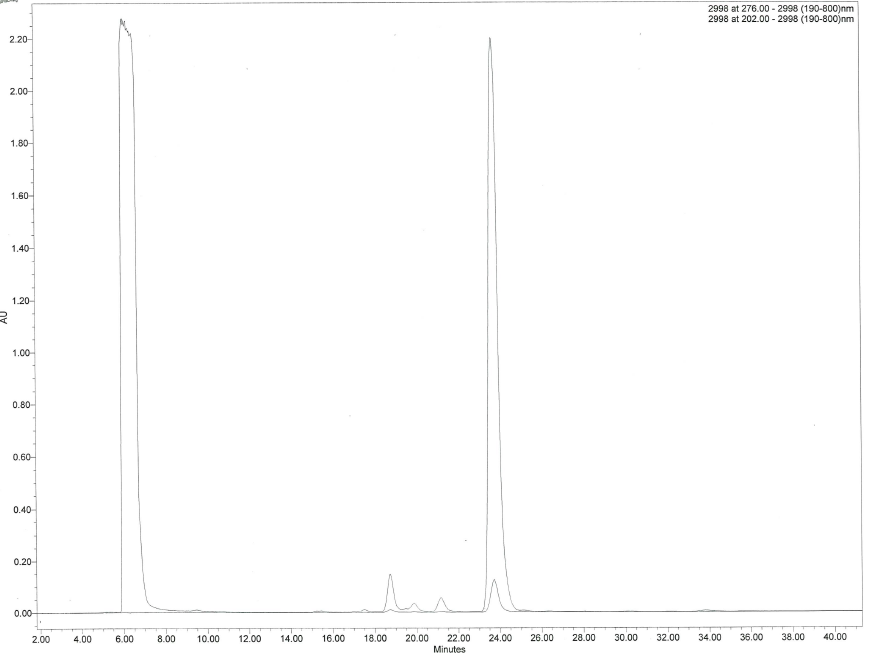


^1^H NMR Spectrum of **BU3-12** (**19**) recorded at 400 MHz in DMSO-*d*_6_


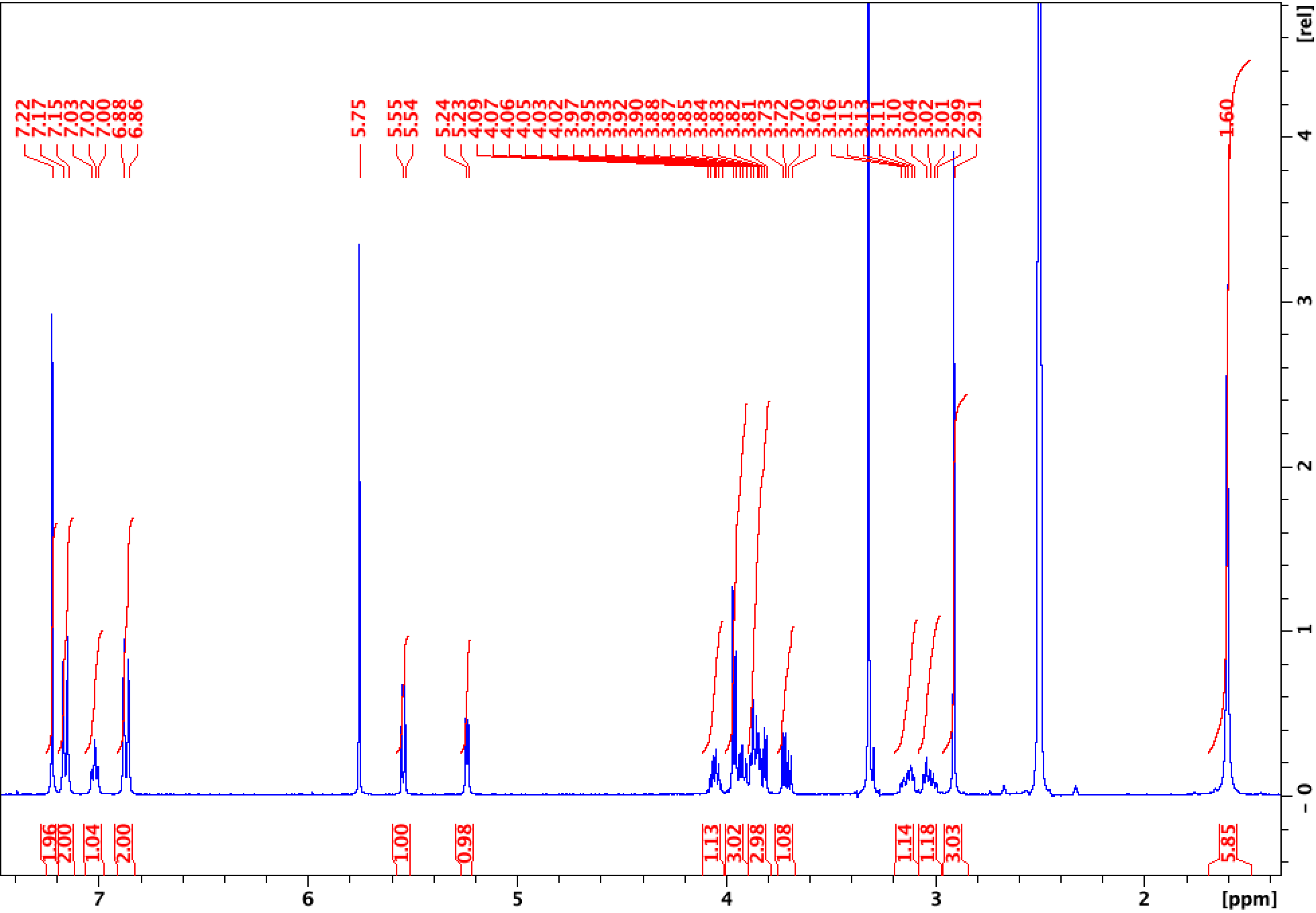


^13^C NMR Spectrum of **BU3-12** (**19**) recorded at 150 MHz in CDCl_3_


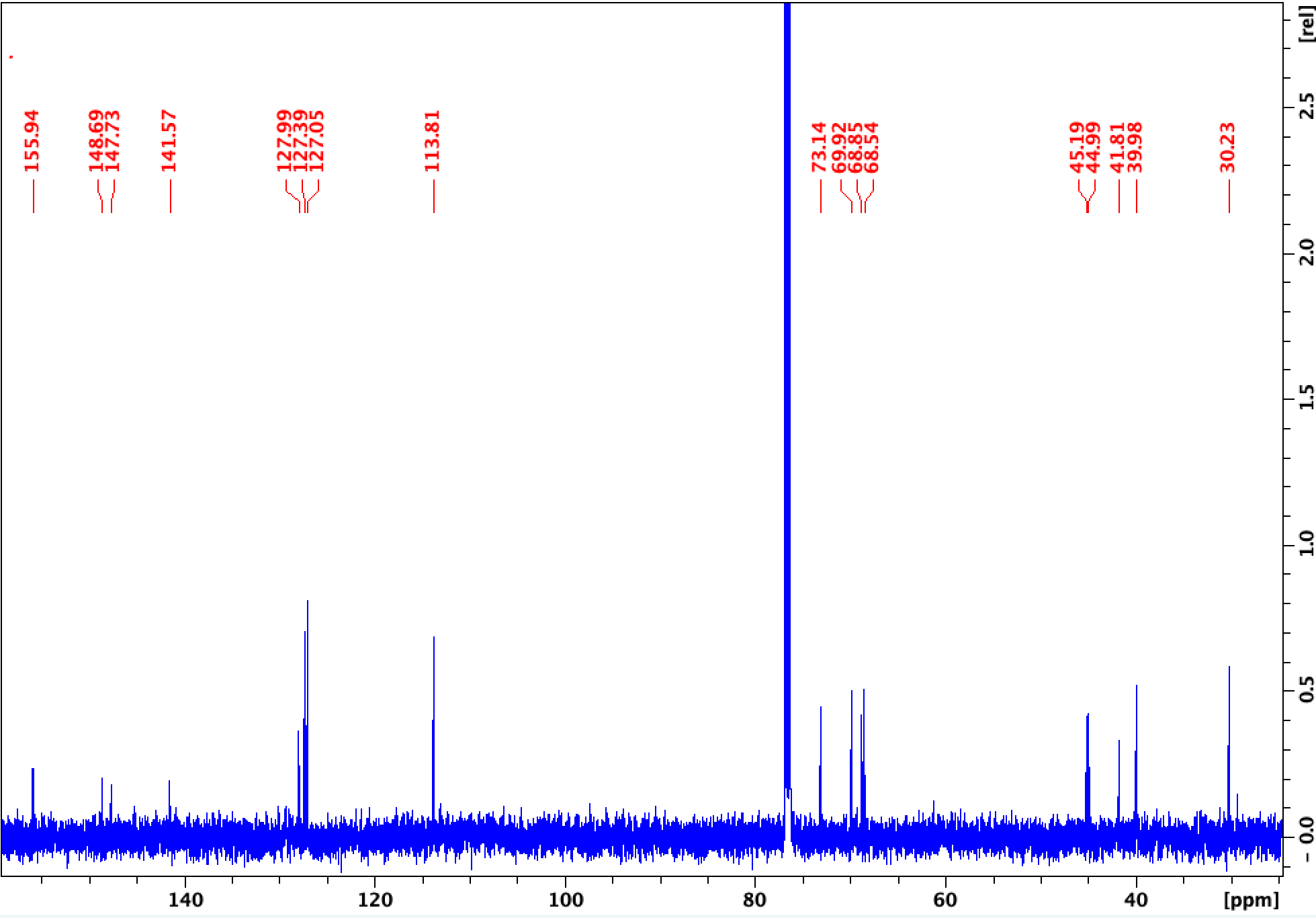


**3.20 BU-81 (20)**

¹H NMR (600 MHz, CDCl_3_) δ 7.12 (s, 2H), 7.11 (d, J = 8.8 Hz, 2H), 6.85 (d, J = 8.8 Hz, 2H), 4.93 (bt, J = 5.8 Hz, 1H), 4.12 (t, J = 6.0 Hz, 2H), 4.11 (d, J = 5.6 Hz, 1H), 4.01 (d, J=10.4 Hz, 1H), 3.95 (d, J = 10.4 Hz, 1H), 3.48 (dd, J = 12.4, 6.2 Hz, 2H), 2.99 (s, 3H), 2.87 (d, J = 4.7 Hz, 1H), 2.74 (d, J = 5.0 Hz, 1H), 2.11 (m, 2H), 1.62 (s, 6H), 1.49 (s, 3H) ppm; ESI-HRMS: *m/z* calculated for C_23_H_29_Cl_3_NO_5_S [M - H]^-^, 536.0832; found, 536.0841.

^1^H NMR Spectrum of **BU-81** (**20**) recorded at 600 MHz in CDCl_3_

**
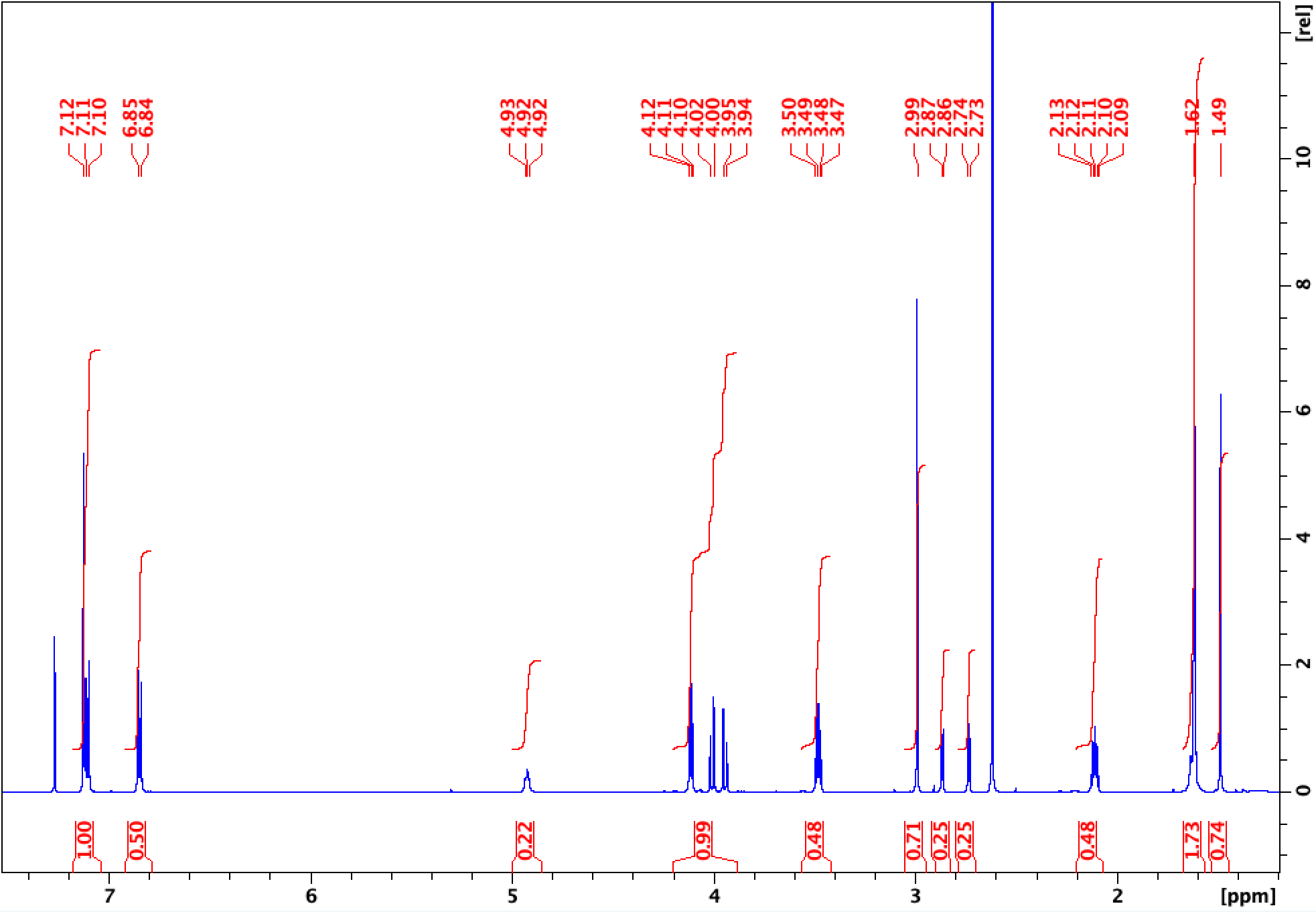
**

**3.21 BU-87 (21)**

¹H NMR (600 MHz, DMSO-*d_6_*) δ 7.32 (s, 2H), 7.15 (d, J = 8.8 Hz, 2H), 7.01 (t, J = 6.1 Hz, 1H), 6.92 (d, J = 8.9 Hz, 2H), 5.24 (d, J = 5.0 Hz, 1H), 5.09 (dd, J = 16.6, 9.7 Hz, 2H), 5.02 (dd, J = 16.2, 9.5 Hz, 2H), 4.10 (t, J = 5.9 Hz, 2H), 3.93 (m, 1H), 3.88 (m, 2H), 3.85 (t, J = 6.5 Hz, 2H), 3.12 (m, 1H), 3.02 (m, 1H), 2.90 (s, 3H), 2.19 (quin, J = 12.4, 6.2 Hz, 2H) ppm; ^13^C NMR (150 MHz, DMSO-*d_6_*) δ 157.7, 149.4, 139.2, 131.3, 128.8, 128.7, 128.2, 114.6, 84.2 (d, J = 170.9 Hz), 70.2, 69.6, 68.1, 51.1 (t, J = 15.0 Hz), 45.5, 41.7, 40.0, 32.6 ppm; ^19^F NMR (300 MHz, DMSO-d_6_) δ -220.0 ppm; ESI-HRMS: *m*/*z* calculated for C_22_H_25_Cl_3_F_2_NO_5_S [M - H]^-^, 558.0493; found, 558.0498.

C_18_ reversed-phase HPLC trace of **BU-87** (**21**) dissolved in DMSO using a InertSustain 5 µm, 25 x 1 cm column with 7:3 MeCN/H_2_O as eluent at a flow rate of 2 mL/min with UV detection at 197 and 228 nm.


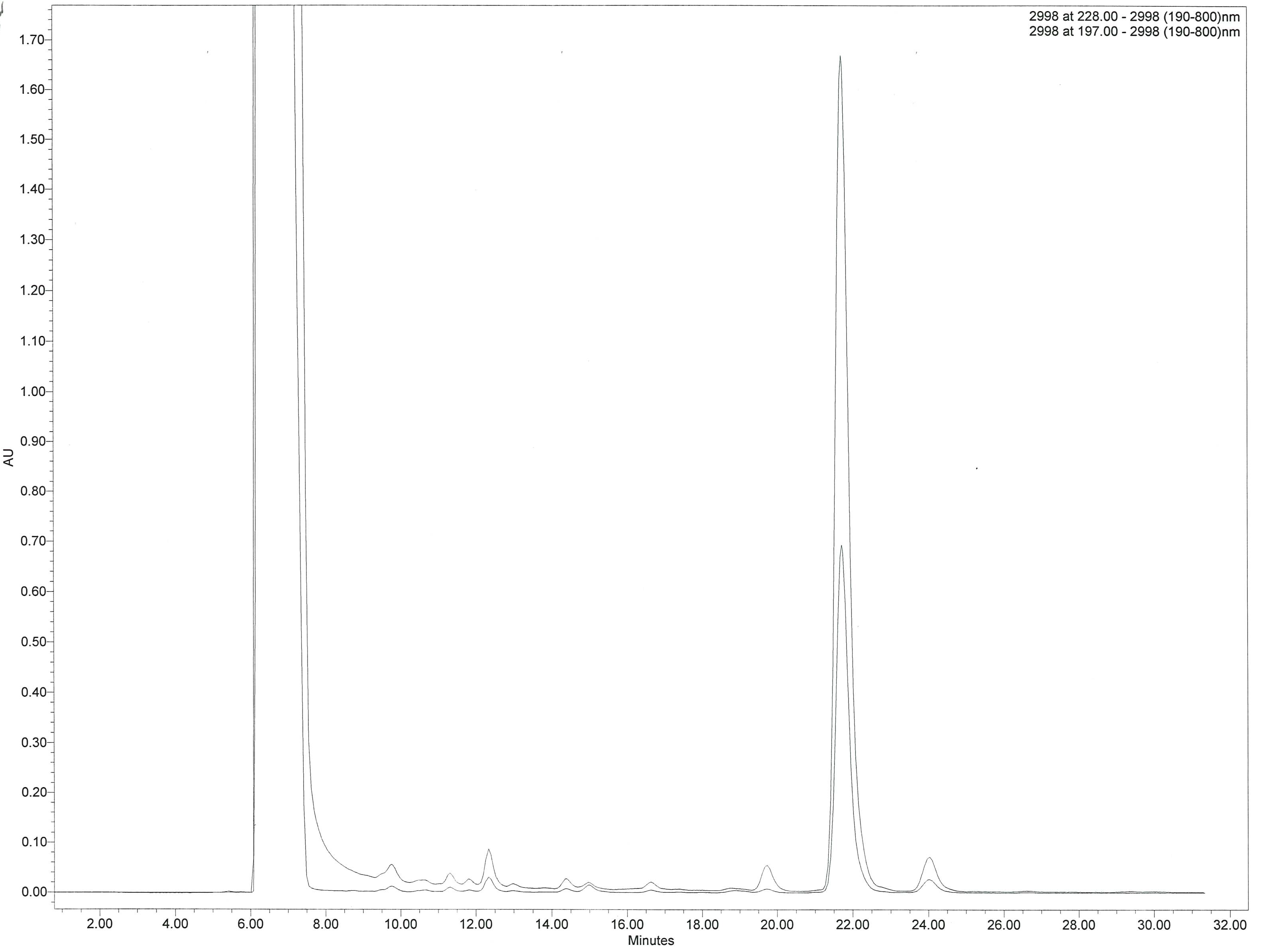


^1^H NMR Spectrum of **BU-87** (**21**) recorded at 600 MHz in DMSO-*d*_6_

^
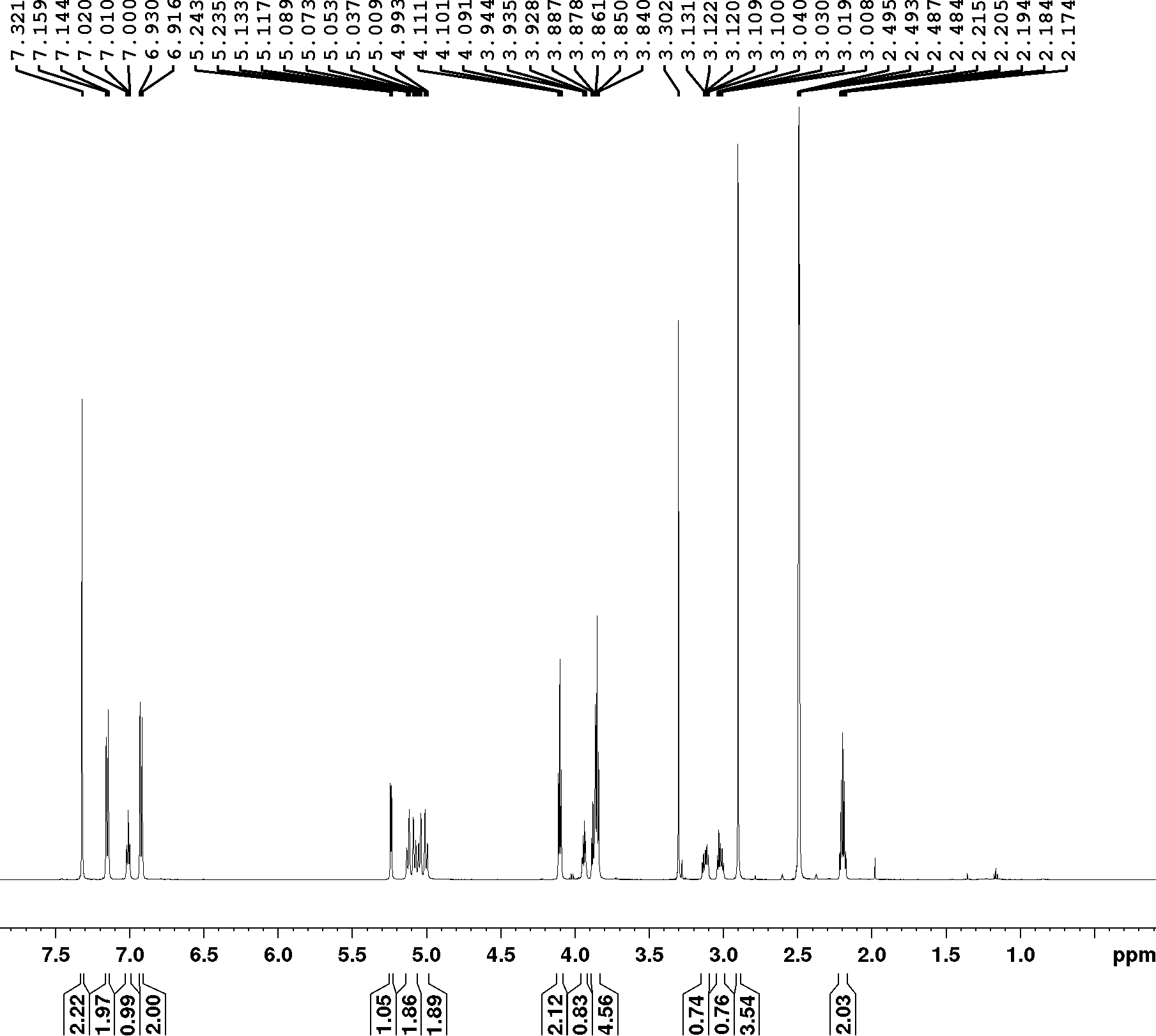
^

^13^C NMR Spectrum of **BU-87** (**21**) recorded at 150 MHz in DMSO-*d*_6_ **
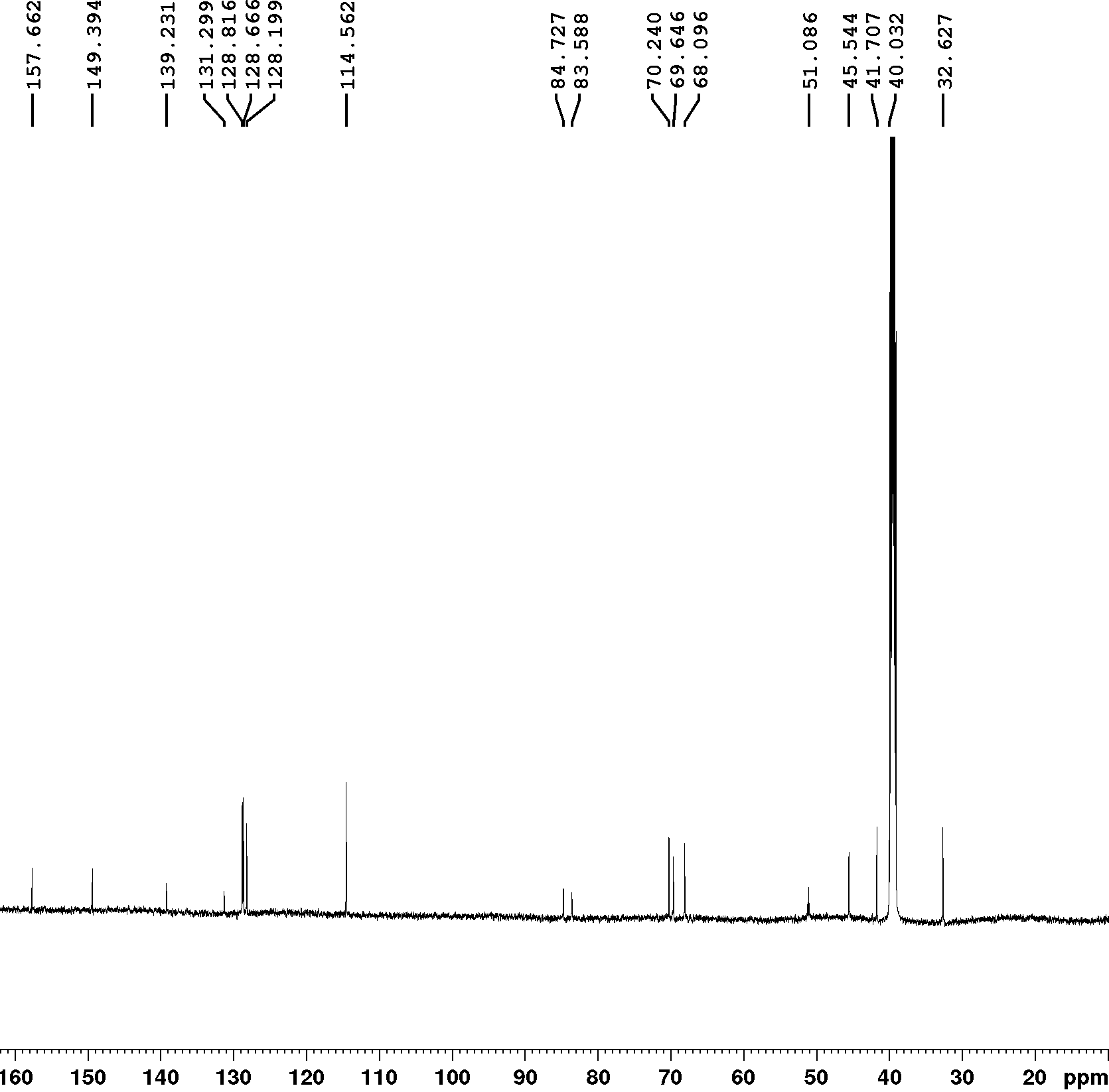
**

**3.22 BU-86 (22)**

¹H NMR (400 MHz, DMSO-*d_6_*) δ 7.34 (s, 2H), 7.17 (d, J = 7.6 Hz, 2H), 7.05 (t, J = 6.1 Hz, 1H), 6.95 (d, 8.5 Hz, 2H), 5.58 (d, 5.2 Hz, 1H), 5.27 (d, 4.4 Hz, 1H), 5.15 (m, 2H), 5.02 (m, 2H), 4.07 (m, 1H), 4.01 (m, 2H), 3.96 (m, 1H), 3.88 (m, 3H), 3.73 (dd, J = 11.2, 5.2 Hz, 1H), 3.14 (m, 1H), 3.04 (m, 1H), 2.93 (s, 3H) ppm; ^13^C NMR (100 MHz, DMSO-*d_6_*) δ 157.7, 128.8, 128.7, 128.0, 122.1, 114.6, 74.2, 69.7, 69.1, 68.1, 46.6, 45.6 ppm; ^19^F NMR (300 MHz, DMSO-d_6_) δ -220.0 ppm; ESI-HRMS: *m*/*z* calculated for C_22_H_25_Cl_3_F_2_NO_6_S [M - H]^-^, 574.0442; found, 574.0450.

^1^H NMR Spectrum of **BU-86** (**22**) recorded at 400 MHz in DMSO-*d*_6_

**
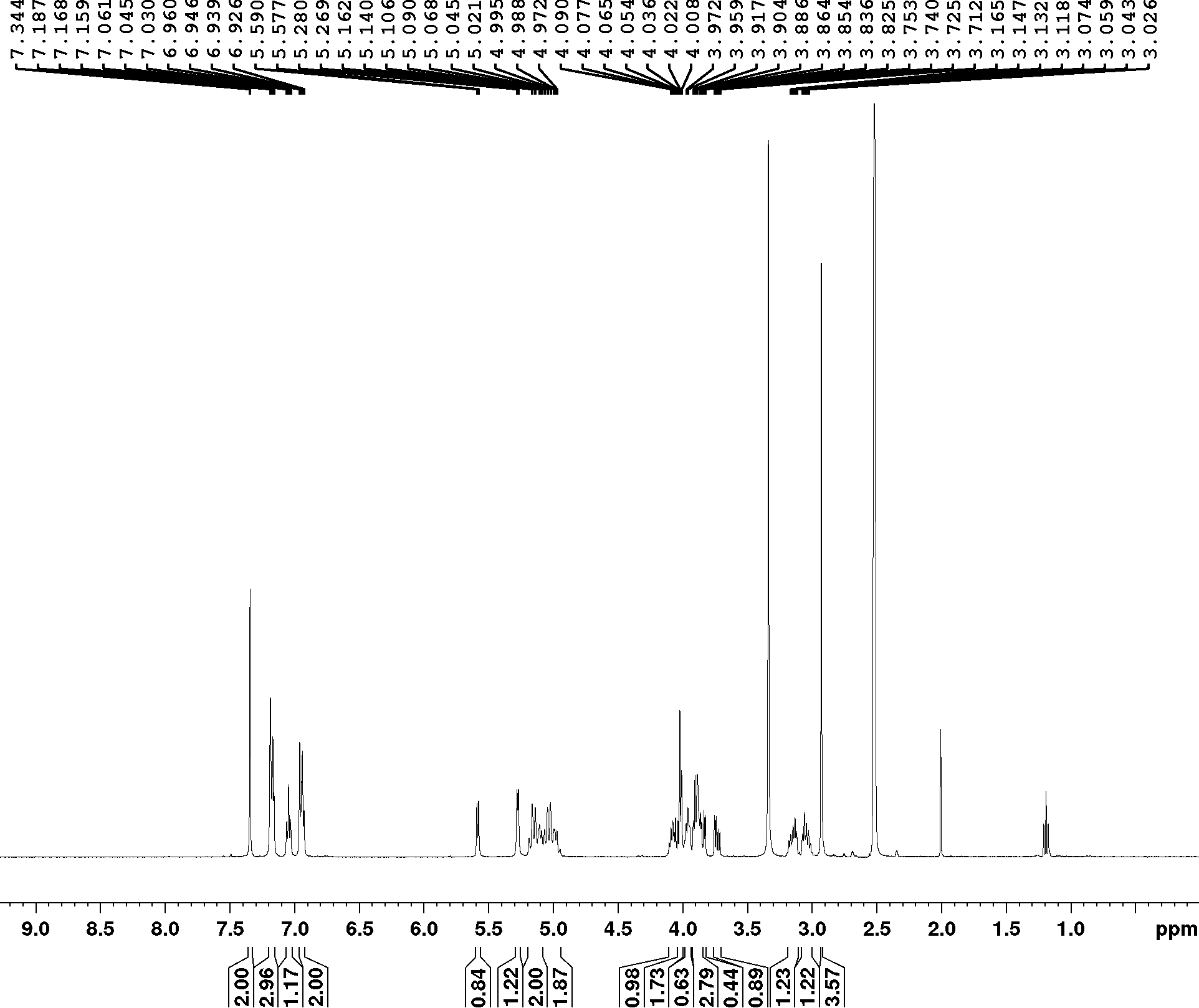
**

^13^C NMR Spectrum of **BU-86** (**22**) recorded at 100 MHz in DMSO-*d*_6_

**
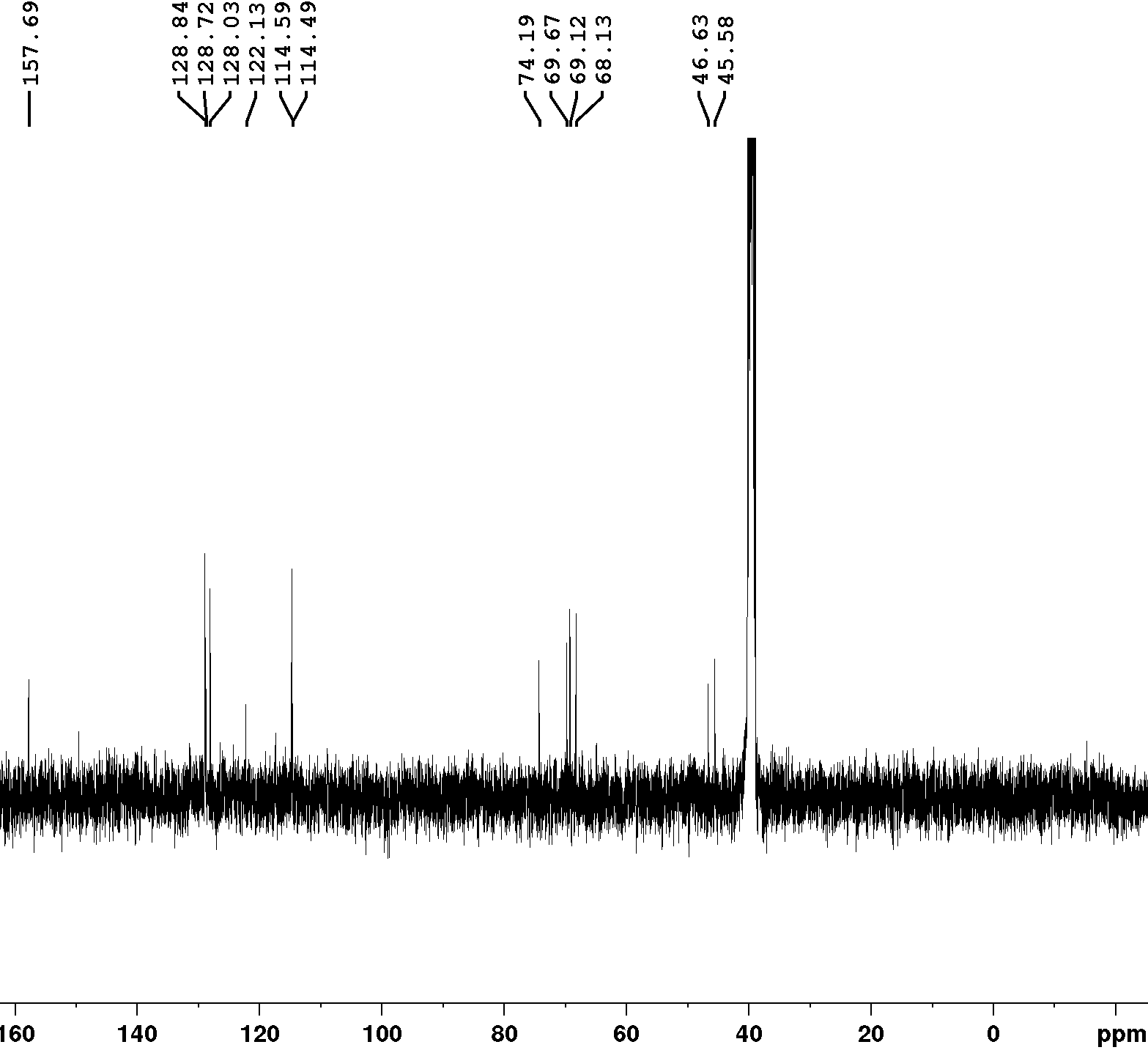
**

**3.23 BU-108 (23)**

^1^H NMR (400 MHz, CDCl_3_) δ 7.18 (s, 2H), 7.13 (d, J = 8.0Hz, 2H), 6.87 (d, J = 8.0 Hz, 2H), 4.97-4.93 (m, 2H), 4.85-4.79 (m, 2H), 4.50 (m, 1H), 4.13 (dd, J = 14.3, 7.2 Hz, 1H), 4.09 (t, J = 5.6 Hz, 1H), 4.04-3.97 (m, 2H), 3.42 (obscured m, 1H), 3.39 (dd, J = 12.8, 6.4 Hz, 1H), 2.97 (s, 3H), 2.89 (d, J = 5.2 Hz, 1H), 2.75 (d, J = 4.8 Hz, 1H), 2.13 (obscured m, 1H), 2.08 (t, J = 5.9 Hz, 1H), 1.59 (s, 3H) ppm.

^1^H NMR Spectrum of **BU-108** (**23**) recorded at 600 MHz in CDCl_3_

**
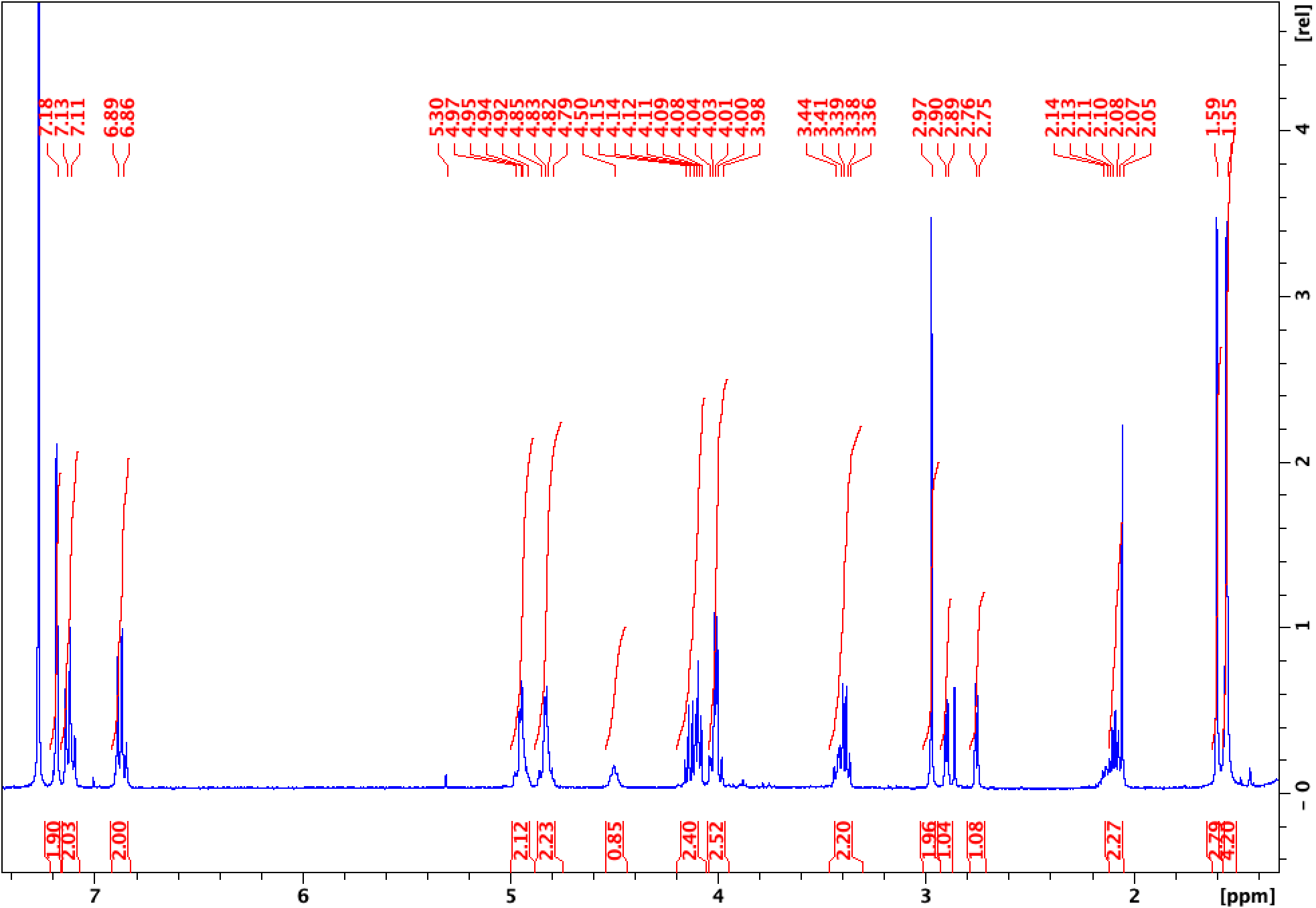
**

**3.24 BU-104 (24)**

^1^H NMR (600 MHz, CDCl_3_) δ 7.18 (s, 2H), 7.13 (d, J = 8.8 Hz, 2H), 6.88 (d, J = 8,9 Hz, 2H), 4.93 (m, 2H), 4.85 (m, 2H), 4.59 (t, J = 6.0 Hz, 1H), 4.18 (t, J = 5.8 Hz, 2H), 4.09 (t, J = 5.7, Hz, 2H), 3.86 (t, J = 6.4 Hz, 2H), 3.38 (m, 2H), 2.97 (s, 3H), 2.29 (tt, J = 6.0, 6.0 Hz, 2H), 2.08 (tt, J = 6.0, 6.0 Hz, 2H) ppm; ^13^C NMR (150 MHz, CDCl_3_) δ 157.87, 150.34, 138.86, 131.95, 129.45, 129.14, 128.79, 114.73, 84.36 (d, J = 186.0 Hz), 69.95, 65.49, 51.87 (t, J = 17.3 Hz), 41.43, 40.90, 40.3, 33.18, 29.58 ppm; ESI-HRMS *m/z* calcd for C_22_H_30_Cl_3_F_2_N_2_O_4_S [M + NH_4_]^+^ 561.0960; found, 561.0954.

^1^H NMR Spectrum of **BU-104** (**24**) recorded at 600 MHz in CDCl_3_


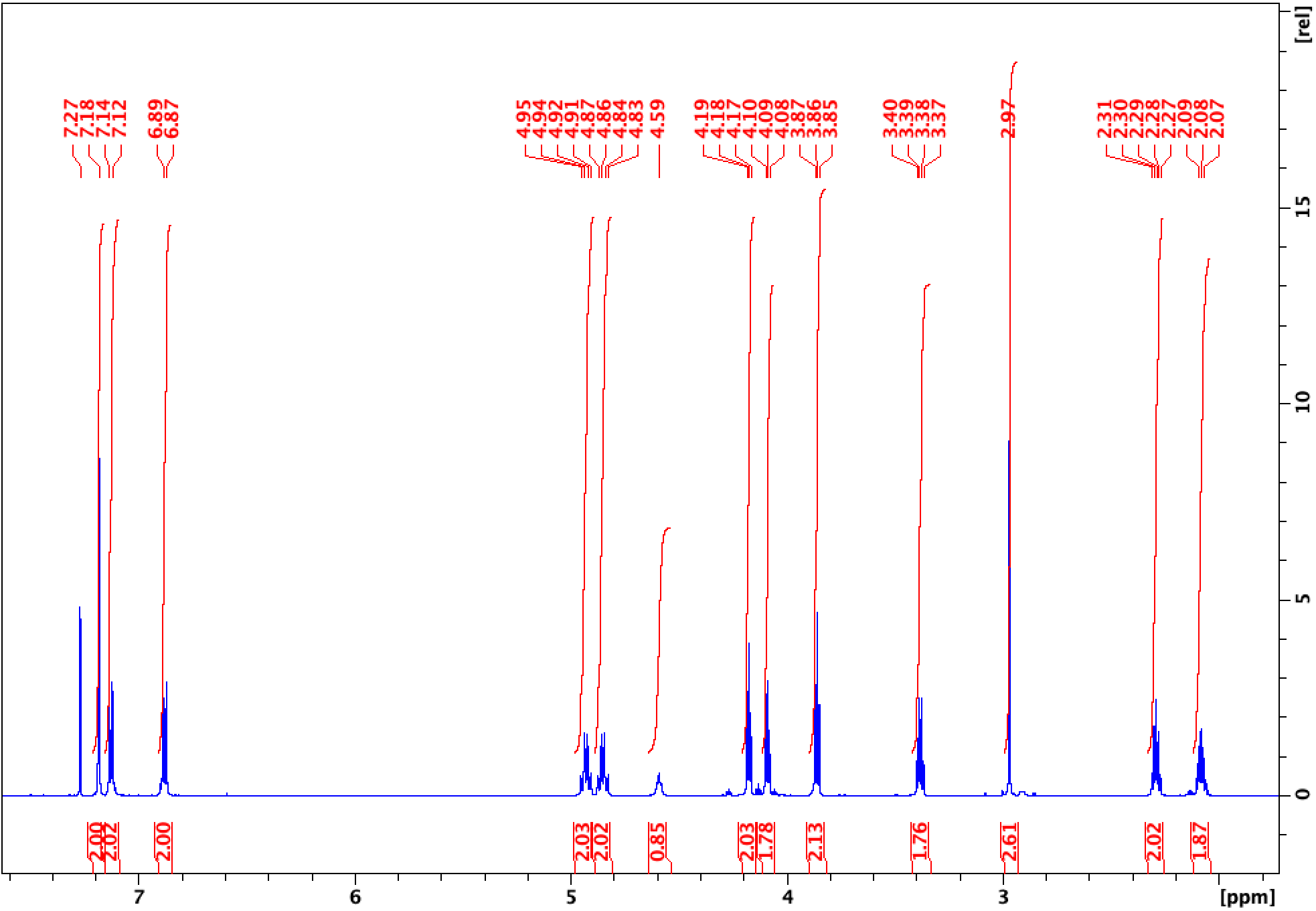


^13^C NMR Spectrum of **BU-104** (**24**) recorded at 150 MHz in CDCl_3_


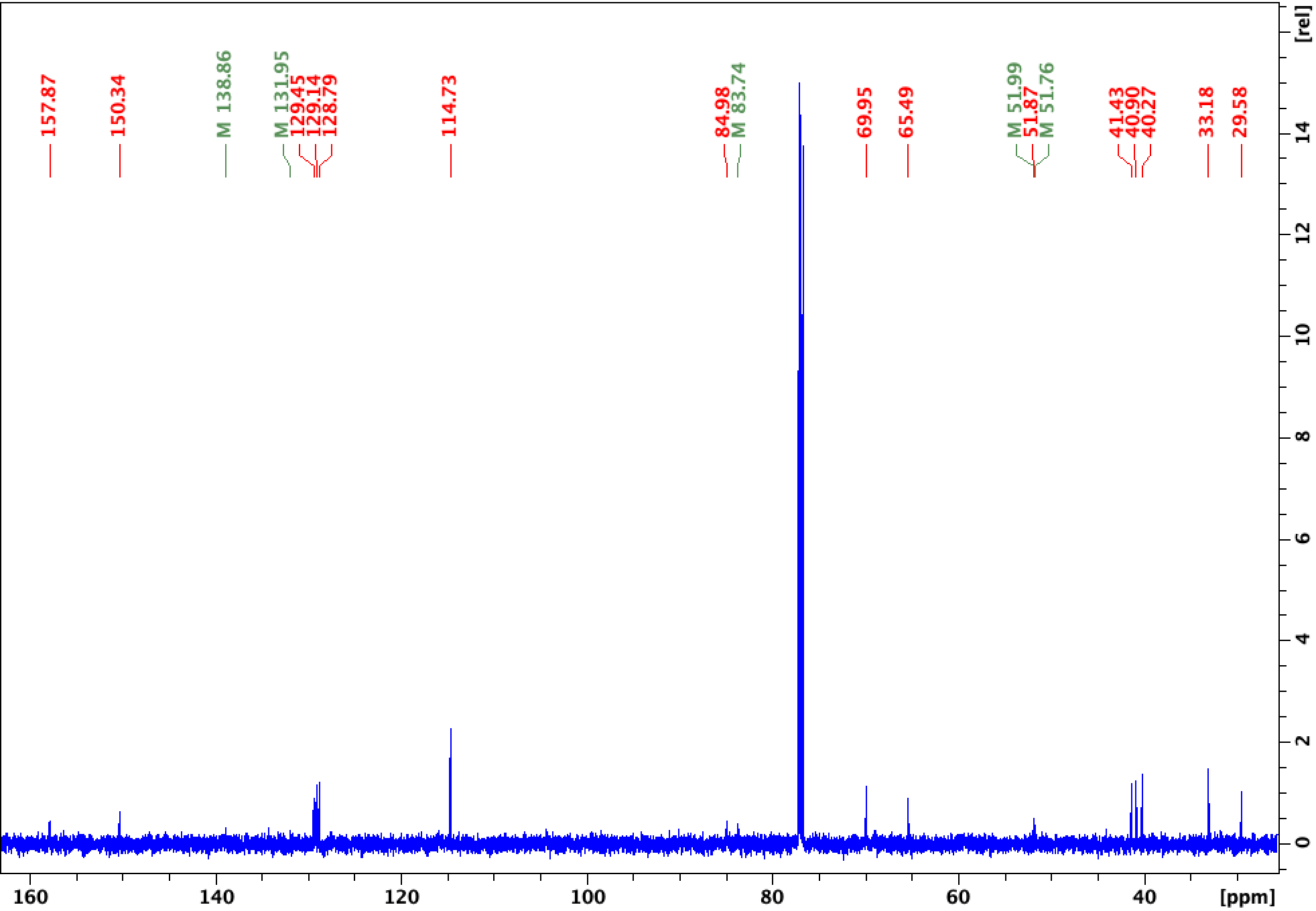


**3.25 BU-130 (25)**

¹H NMR (400 MHz, DMSO-*d_6_*) δ 7.33 (s, 2H), 7.15 (d, J = 8.8 Hz, 2H), 6.92 (d, J = 8.9 Hz, 2H), 6.90 (t, J = 6.5 Hz, 1H), 5.13 (m, 2H) 5.01 (m, 2H), 4.89 (s, 1H), 4.11 (t, J = 6.0 Hz, 2H), 3.86 (t, J = 6.5 Hz, 2H), 3.80 (d, J = 9.3 Hz, 1H), 3.74 (d, J = 9.3 Hz, 1H), 3.04 (m, 2H), 2.89 (s, 3H), 2.20 (tt, J = 6.0, 6.0 Hz, 2H), 1.18 (s, 3H) ppm; ^19^F NMR (300 MHz, DMSO-*d_6_*) δ -222.0 ppm; ESI-HRMS: *m*/*z* calculated for C_23_H_27_Cl_3_F_2_NO_5_S [M - H]-, 572.0649; found, 572.0653.

C_18_ reversed-phase HPLC trace of **BU-130** (**25**) dissolved in DMSO using a InertSustain 5 µm, 25 x 1 cm column with 7:3 MeCN/H_2_O as eluent at a flow rate of 2 mL/min with UV detection at 197 and 228 nm.


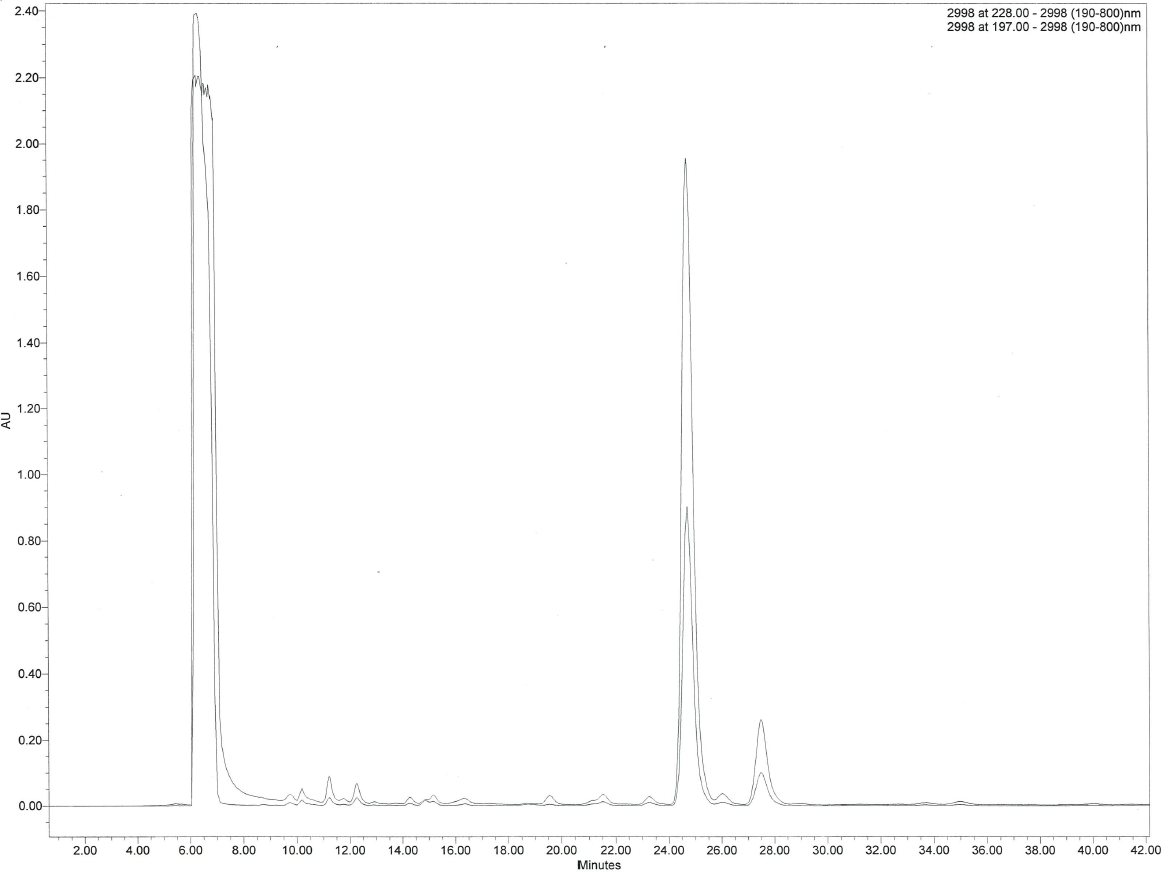


^1^H NMR Spectrum of **BU-130** (**25**) recorded at 400 MHz in DMSO-*d*_6_


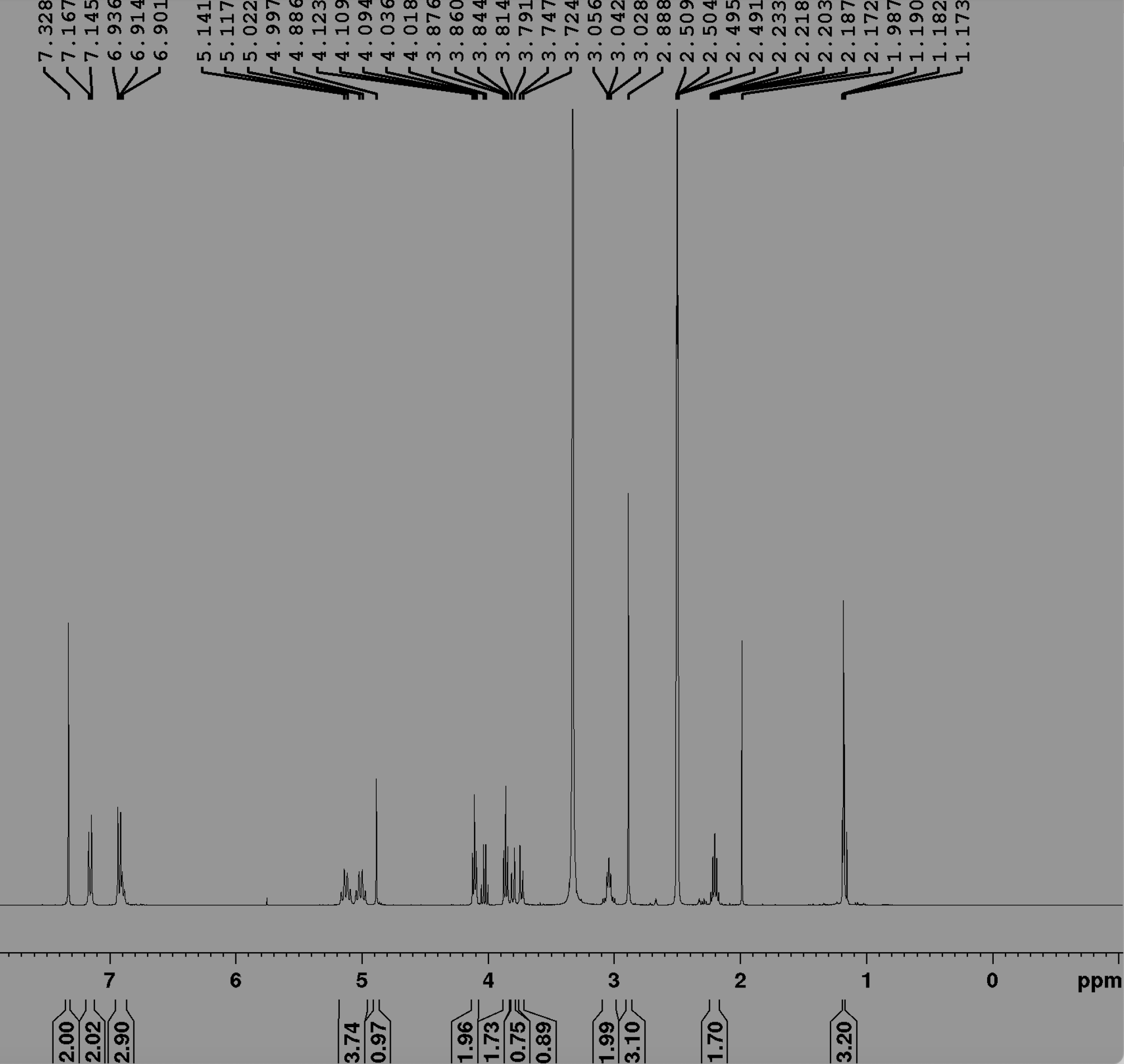


**3.26 BU170 (26)**

¹H NMR (400 MHz, DMSO-*d_6_*) δ 7.32 (s, 2H), 7.15 (d, J = 8.8 Hz, 2H), 6.93 (d, J = 8.9 Hz, 2H), 6.90 (t, J = 6.6 Hz, 1H), 5.57 (d, J = 5.3 Hz, 1H), 5.13 (dd, J = 9.8, 9.8 Hz, 2H), 5.01 (dd, J = 9.8, 9.3 Hz, 2H), 4.89 (s, 1H), 4.06 (m, 1H), 4.00 (d, J = 5 Hz, 2H), 3.81 (m, 2H), 3.72 (m, 2H), 3.04 (m, 2H), 2.89 (s, 3H), 1.17 (s, 3H) ppm; ^13^C NMR (150 MHz, DMSO-*d_6_*) δ 157.8, 149.4, 128.8, 128.7, 128.0, 122.1, 114.6, 114.5, 74.2, 72.4, 70.4, 69.1, 51.1, 49.4, 46.6, 22.6, 20.7 ppm; ^19^F NMR (300 MHz, DMSO-*d_6_*) δ -222.0 ppm; ESI-HRMS: *m/z* calculated for C_23_H_28_Cl_3_F_2_NO_6_S [M - H]^-^, 589.0671; found, 589.0670.

C_18_ reversed-phase HPLC trace of **BU-170** (**26**) dissolved in DMSO using a InertSustain 5 µm, 25 x 1 cm column with 3:2 MeCN/H_2_O as eluent at a flow rate of 2 mL/min with UV detection at 197 and 274 nm.


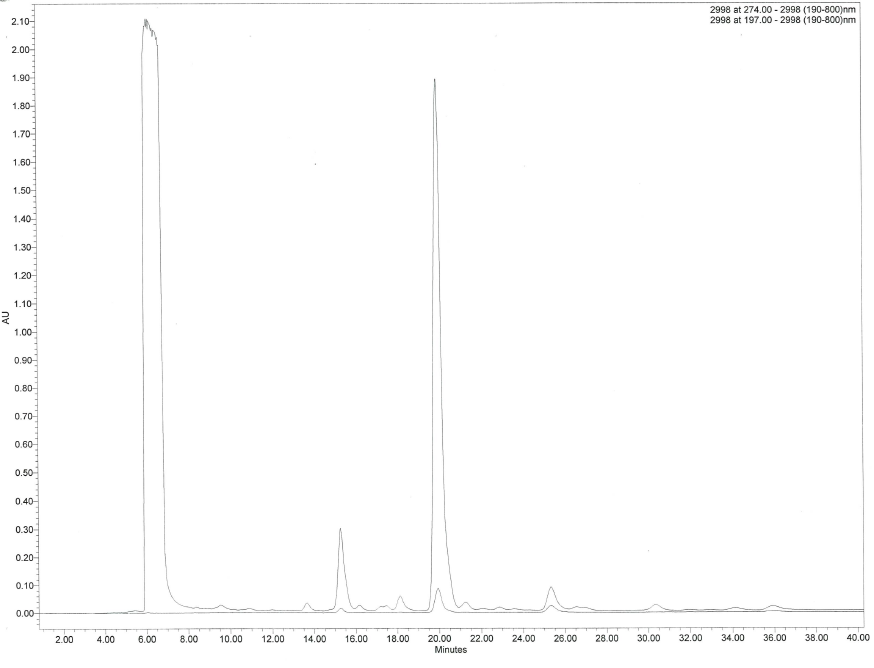


^1^H NMR Spectrum of **BU-170** (**26**) recorded at 400 MHz in DMSO-*d*_6_


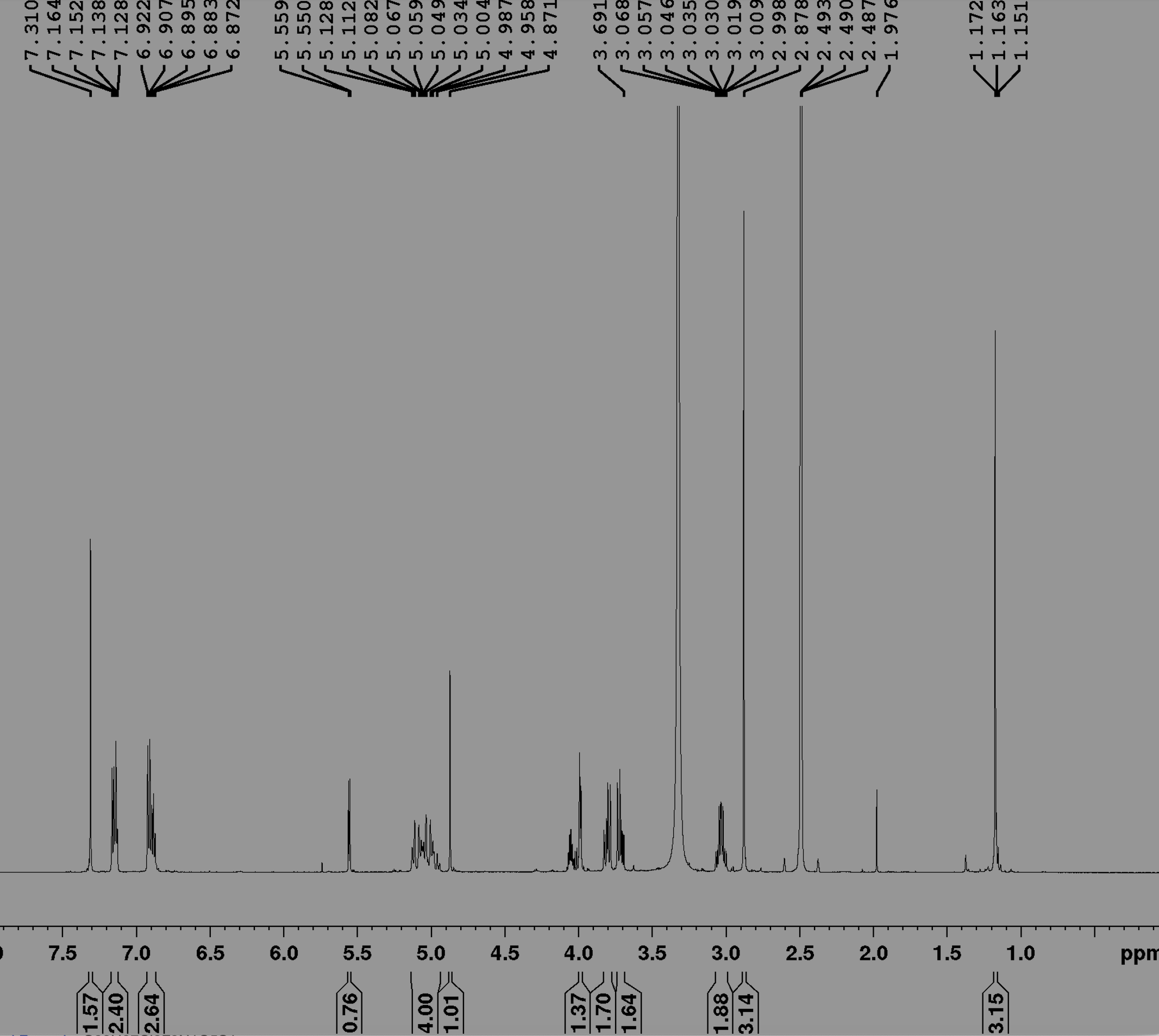


^13^C NMR Spectrum of **BU-170** (**26**) recorded at 150 MHz in DMSO-*d*_6_


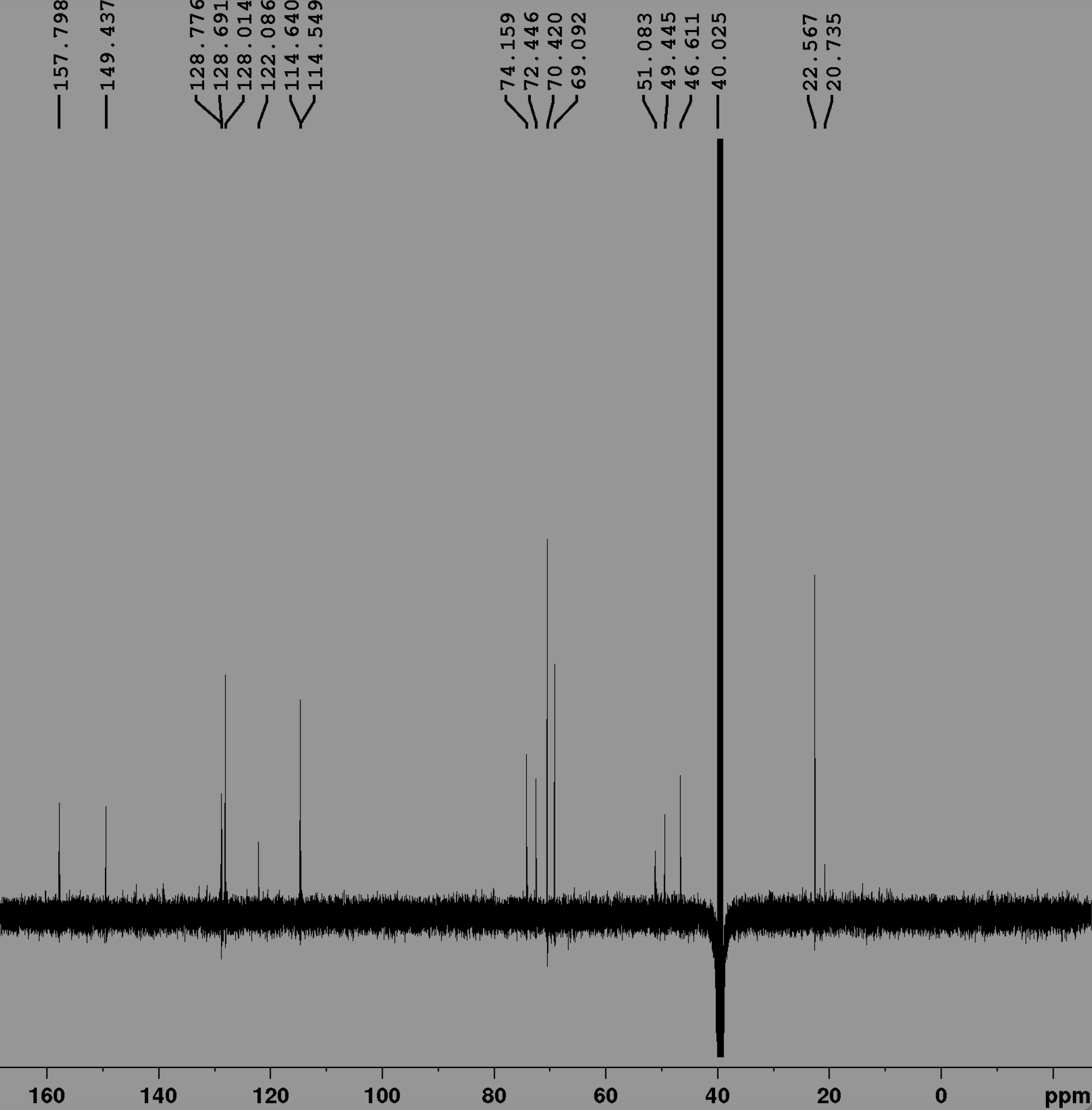

Supplement: Supplementary file 5 — S5 Synthesis and Compound Characterization [file 41392_2026_2642_MOESM5_ESM.docx]
